# The strength of the template effect attracting nucleotides to naked DNA

**DOI:** 10.1093/nar/gku314

**Published:** 2014-05-28

**Authors:** Eric Kervio, Birgit Claasen, Ulrich E. Steiner, Clemens Richert

**Affiliations:** 1 Institute for Organic Chemistry, University of Stuttgart, 70569 Stuttgart, Germany; 2 Fachbereich Chemie, Universität Konstanz, 78457 Konstanz, Germany

## Abstract

The transmission of genetic information relies on Watson–Crick base pairing between nucleoside phosphates and template bases in template–primer complexes. Enzyme-free primer extension is the purest form of the transmission process, without any chaperon-like effect of polymerases. This simple form of copying of sequences is intimately linked to the origin of life and provides new opportunities for reading genetic information. Here, we report the dissociation constants for complexes between (deoxy)nucleotides and template–primer complexes, as determined by nuclear magnetic resonance and the inhibitory effect of unactivated nucleotides on enzyme-free primer extension. Depending on the sequence context, *K*_d_′s range from 280 mM for thymidine monophosphate binding to a terminal adenine of a hairpin to 2 mM for a deoxyguanosine monophosphate binding in the interior of a sequence with a neighboring strand. Combined with rate constants for the chemical step of extension and hydrolytic inactivation, our quantitative theory explains why some enzyme-free copying reactions are incomplete while others are not. For example, for GMP binding to ribonucleic acid, inhibition is a significant factor in low-yielding reactions, whereas for amino-terminal DNA hydrolysis of monomers is critical. Our results thus provide a quantitative basis for enzyme-free copying.

## INTRODUCTION

Step-wise extension of a growing oligonucleotide chain by nucleotides, directed by a template, is the molecular basis of replication and transcription (1,2). This process is found in all kingdoms of life. Its rate and fidelity are critical for the survival of species, both in terms of passing on genetic information, and in terms of allowing for mutations (3). Watson–Crick base pairing is known to be the guiding principle of nucleobase selection during replication, but numerous factors affect the rates of polymerase-catalyzed extension (4–6). Some alternative base pairs are accepted by polymerases (7–10), but it is not easy to dissect the contributions that the active site and the template/primer duplex make to the interactions driving the incorporation of nucleotides. Some dNTP analogs are readily incorporated by polymerases, even though their base pairs destabilize duplexes (11–13).

One field where the strength of the template effect provided by base pairing between nucleotides and templates is particularly important is enzyme-free replication (14). Enzyme-free or ‘chemical’ primer extension, is solely driven by the template effect experienced by an incoming nucleotide binding to primer–template complexes and the intrinsic reactivity of the monomer (15). A quantitative understanding of this reaction is critical for theories on how life might have arisen during an early phase of evolution (16,17). It is currently unclear whether the template effect provided by a template:primer complex is sufficient to allow for enzyme-free copying of sequences long enough to act as polymerase ribozymes (18,19).

Enzyme-free copying was first demonstrated for ribonucleic acid (RNA) (20–24). Detailed studies suggested that only sequences rich in cytidylic acid were able to induce the spontaneous formation of complementary strands (25), and the prospect of replication in systems containing all four nucleotides was called ‘remote’ (26). Later work showed that with oxyazabenzotriazole leaving groups, low temperatures and downstream-binding oligonucleotides acting as ‘helper strands’ rates are accelerated and yields improve (27,28). Further, a competitive inhibition by unactivated nucleotides (produced through hydrolysis in the time course of an assay) can be avoided when template and primer are immobilized and the supernatant containing the monomers is removed periodically (29). A similar approach has recently been implemented for vesicles, using periodic dialysis against solutions of activated monomers (30). In favorable cases, extension by any of the four nucleotides (A/C/G/U) opposite their canonical base pairing partner occurs (29). This approach requires intervention and well defined conditions, though (31), and serious challenges remain for monomer-based self-replicating systems (32).

Fast rates in enzyme-free primer extension can be achieved by using amino-terminal primers, whose extension by reactions with activated monomers produces genetic polymers with phosphoramidate linkages that are isoelectronic to natural phosphodiesters (33–39). With amino-terminal primers, high yields were observed for any of the 64 triplets whose center nucleotide provides the templating base (40). Further, slow extension of primers after a misincorporation has been shown to improve the fidelity of copying of longer sequences, thus avoiding a potential ‘error catastrophe’ in enzyme-free replication (41–43). What has remained unclear, though, is what fraction of the reactivity observed in the extension of amino-terminal primers is due to template-independent chemical reactivity and what fraction is due to the template effect. Overextension of the primer, beyond the length of template, suggests that the contribution of non-templated polymerization can be significant (44). Without a quantitative understanding of the template effect, the question of whether longer sequences can be copied under prebiotically plausible conditions remains difficult to answer. A quantitative understanding of the template effect will also help to understand how much the substrates contribute to the fidelity of polymerase-catalyzed polymerization. Finally, such data will help to develop new methods for reading out genetic information in enzyme-free fashion (45,46).

**Figure 1.**
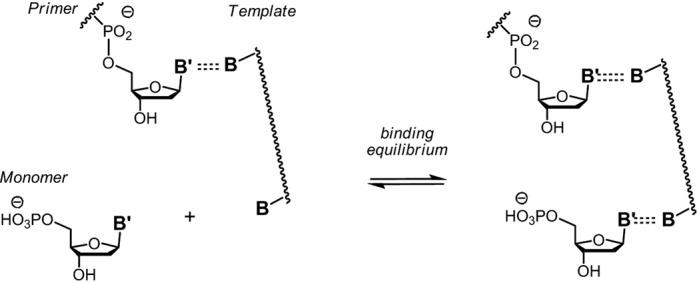
Binding equilibrium between nucleotide and primer–template duplex; **B**, **B′** = nucleobases.

Base pairing between isolated nucleobases has been studied in organic solvents (47,48). But, there appear to be no experimental binding constants for complexes between nucleotides and primer–template duplexes (Figure 1), even though there has been intense theoretical work (49–51). For example, Bickelhaupt and colleagues have calculated binding energies for model complexes of template–primer duplexes and incoming nucleoside phosphates. Their Δ*G*_affinity_ values range from −20.8 kcal/mol for an incoming G pairing with C as templating base, when intrinsic thermal and entropy effects are ignored, and −3.1 kcal/mol for an incoming T pairing with A when a large estimated value for such effects is used.

Here, we report binding constants for the complexes of nucleotides with primer-template duplexes, as determined by two complementary techniques. A quantitative model presented produces time-dependent yields of enzyme-free extension. Our data has allowed us, for the first time, to understand the concentration and sequence dependence of enzyme-free primer extension, as well as the inhibitory effect of hydrolyzed monomers.

## MATERIALS AND METHODS

### Activated monomers and primers

Oxyazabenzotriazolides (OAt esters) of nucleotides (dNMPs or GMP) were prepared via activation of with EDC/HOAt (35). Primers with 3′-terminal 3′-amino-2′,3′-dideoxynucleoside residue were prepared on as described previously (52) and were purified by HPLC. Unmodified DNA strands were purchased from Biomers (Ulm, Germany) in salt-free form and were used without further purification. Unmodified hairpin sequences and the RNA hairpin were purchased from Biospring (Frankfurt, Germany). Further details can be found in the Supplementary material.

### NMR experiments

Nuclear magnetic resonance (NMR) samples (200 μL) were prepared in 3 mm semimicro tubes and were 0.5 mM in the hairpin oligonucleotide. Spectra were recorded on a Bruker Avance 500 spectrometer. Signal assignment was based on a combination of NOESY and TOCSY spectra with presaturation to suppress the solvent signal. Data processing used an exponential function with a line broadening setting of 0.3 Hz. For representative two-dimensional (2D) spectra and more detailed protocols, please see the Supplementary material.

### Primer extension assays

Primer extension assays with MALDI-ToF-based analysis were performed as previously described (40). For inhibitor assays, experiments were typically performed as follows. To the assay solution (10 μL final volume) containing the primer (36 μM), the template (54 μM) and, unless noted otherwise, the downstream-binding oligonucleotide (54 μM) in HEPES buffer (200 mM, 400 mM NaCl, 80 mM, pH 8.9), the unactivated 3′-deoxynucleotide (**1a–t**) was added using an aliquot of a stock solution (72 mM) in assay buffer. Assays were started by addition of an aliquot of the aqueous stock solution of the OAt-esters of 3′-deoxynucleotides (final concentration 3.6 mM for **7a**, **7c** or **7g**, and 7.2 mM for **7t**). Additional details and data can be found in the Supplementary material.

## RESULTS

### Nucleotides binding to terminal template bases

**Figure 2.**
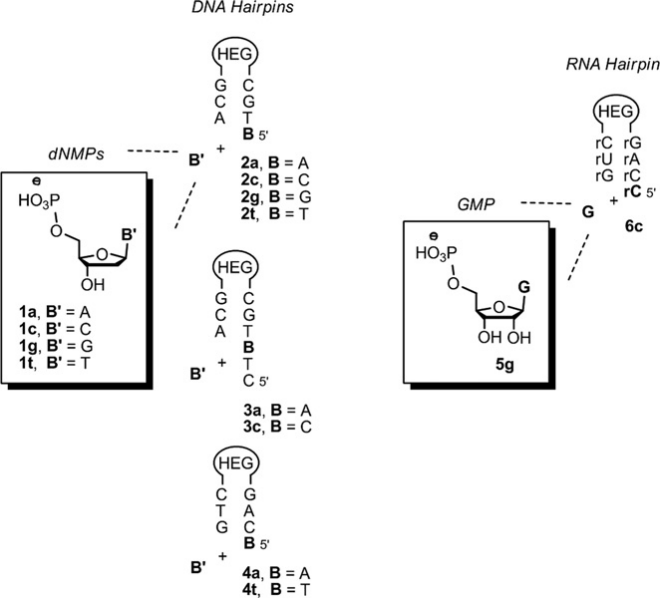
Nucleotides and hairpins used for NMR titration. Loops are hexaethylene glycol linkers (HEG).

Two experimental systems were used to measure the binding of nucleotides to templating bases (Figure 1). The first used NMR spectroscopy as monitoring technique and short hairpins as intramolecular models of primer–template duplexes (Figure 2). The overhang at the 5′-terminus of the hairpin provided the templating base to which deoxynucleoside monophosphates (dNMPs) **1a**–**t** were allowed to bind. The stem of the hairpin oligonucleotides was chosen to be long enough to give a stable helix at room temperature, but short enough to allow for NMR without isotope enrichment. A hexaethylene glycol (HEG) linker loop (53) was chosen to provide stability without complicating assignment.

**Figure 3.**
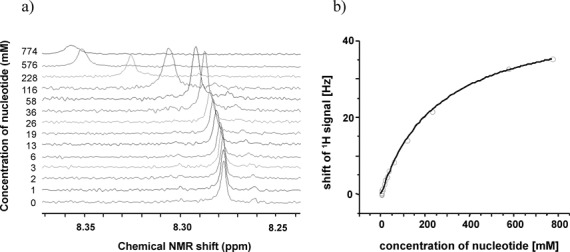
Typical results from NMR titrations. (**a**) Overlay of spectra with ^1^H-NMR signal of H-8 proton of the 5′-terminal A residue of hairpin 5′-ATGC(HEG)GCA (**2a**) (0.5 mM) with increasing concentration of TMP (**1t**) (0 to 774 mM) at 20°C. (**b**) Plot of chemical shift displacement upon addition of TMP (**1t**). See Supplementary Figures S8, S9 and S12–S14 (Supplementary material) for additional spectra and plots of chemical shifts.

A series of ^1^H NMR spectra of hairpins **3a** and **3c** at increasing temperature in solutions with buffer conditions typical for primer extensions (35) confirmed that the melting transition of the helices was above 40°C (see Supplementary Figures S3–S5, Supplementary material). The assignment of the hairpin NMR signals was based on 2D spectra and a literature-known assignment strategy (54). Titrating deoxynucleotides into the NMR solution of hairpins led to downfield or upfield shifts of resonances of the terminal residues of the hairpin (Figure 2 and Supplementary Figures S8–S9 in the Supplementary Material). Curve fittings to plots of shifts against nucleotide concentrations then gave binding constants (see Supplementary material, Supplementary Figures S12–S14 for details) (55,56) (Figure 3).

Binding constants for complementary pairs of nucleotide and templating base were found to range from 10 to 280 mM, depending on the templating base, the length of the overhang, and the base at the 3′-terminus of the primer segment of the hairpin (Table 1). Large changes in *K*_d_ were observed when switching from one templating base to another. The binding of thymidine 5′-monophosphate (TMP, **1t**) to adenine-displaying hairpins **2a**, **3a** and **4a** is ∼20-fold weaker than that that of dGMP (**1g**) to hairpins **2c** and **3c**. The *K*_d_ values for 2′-deoxycytidine 5′-monophosphate (dCMP, **1c**) and dAMP (**1a**) binding to the hairpins displaying their complementary base as templating residue (**2g** and **4t**) are in between those measured for TMP and GMP, with values of 38 and 40 mM, respectively. The base at the terminus of the hairpin helix has a modest effect on the *K*_d_. A mismatch between incoming nucleotide and templating base leads to ∼10-fold drop in *K*_d_ for a G:T wobble pair (**1g** binding to **4t**) and a more than 100-fold drop for a C:T base combination (**1c** and **4t**). An exploratory measurement with an all-RNA system (**5g**:**6c**) gave a *K*_d_ of 14 mM, which is close to that measured for the same G:C pairing in the DNA hairpins (**1g**:**2c** and **1g**:**3c**).

**Table 1.** Dissociation constants for nucleotides binding to hairpin termini, as determined by NMR titration^a^

Nucleotide	Templating base	Hairpin		*K*_d_ (mM) ^b^
d**T**MP (**1t**)	**A**	3′-ACG(HEG)CGT**A**	(**2a**)	260
d**T**MP (**1t**)	**A**	3′-ACG(HEG)CGT**A**TC	(**3a**)	280
d**T**MP (**1t**)	**A**	3′-GTC(HEG)GAC**A**	(**4a**)	240
d**G**MP (**1g**)	**C**	3′-ACG(HEG)CGT**C**	(**2c**)	10
d**G**MP (**1g**)	**C**	3′-ACG(HEG)CGT**C**TC	(**3c**)	16
d**G**MP (**1g**)	**T** (wobble pair)	3′-GTC(HEG)GAC**T**	(**4t**)	100
d**C**MP (**1c**)	**G**	3′-ACG(HEG)CGT**G**	(**2g**)	40
d**C**MP (**1c**)	**T** (mismatch)	3′-GTC(HEG)GAC**T**	(**4t**)	≥2000^d^
d**A**MP (**1a**)	**T**	3′-GTC(HEG)GAC**T**	(**4t**)	38
*r***G**MP (**5g**)	*r***C** (RNA)	3′-*r*(GUC(HEG)GAC**C**)^c^	(**6c**)	14

^a^Conditions: 0.5 mM hairpin in D_2_O and 200 mM phosphate buffer, 400 mM NaCl, 80 mM MgCl_2_, pH 8.9, uncorrected for deuterium effect, 20°C. Bold letters in hairpin sequences are templating bases; HEG, hexaethylene glycol linker.

^b^Determined by fit.

^c^Oligoribonucleotide (2 mM) and pH 7 to avoid hydrolysis of RNA under more basic conditions.

^d^No saturation observed upon addition of up to 2000 eq. dCMP (1 M).

### Binding in the interior of longer sequences

**Figure 4.**
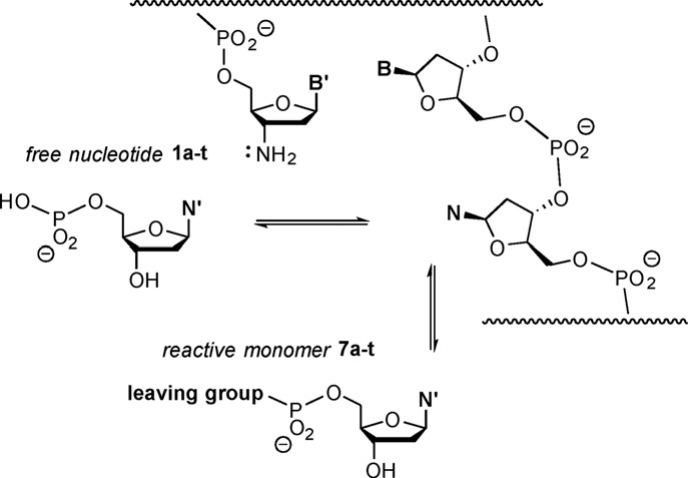
Binding equilibria underlying the inhibitory effect of a free nucleotide on chemical primer extension.

We then developed an approach for measuring binding of deoxynucleotides to the primer extension site of longer DNA templates in the presence or absence of downstream-binding oligodeoxynucleotides. We used the inhibitory effect of free nucleotides added to primer extension mixtures for our measurements. The free nucleotide competes with the activated nucleotide for the primer extension site, thus inhibiting the reaction (Figure 4).

Monoexponential fits to data sets from a series of kinetics runs with increasing concentration of unactivated deoxynucleotide gave inhibitory constants (*K*_Inh_), from which dissociation constants (*K*_d_) were calculated. The kinetic analysis was based on the following model. (A discussion of its implications and a more sophisticated model can be found in the Supplementary material.) The primer extension reaction

(1) \begin{equation*} \text{M} + \text{P}_1 \xrightarrow{{k = k'[\text{M}]}}\text{P}_2 \end{equation*}

with M the monomer, P_1_ the primer–template complex, P_2_ the extended primer, and *k*′ the second order rate constant, is treated as a pseudo-first-order reaction with the effective rate constant *k*′ [M]. This approach is justified because the monomer M was present in large excess. The unactivated nucleotide acting as inhibitor (Inh) and the primer–template complex are in a fast equilibrium with the inhibitor–primer–template complex Inh – P_1_

(2) \begin{equation*} \text{Inh} + \text{P}_1 \underset{{K_\text{d} }}{\overset{{K_{\text{Inh}} }}{\leftrightarrows}}\text{Inh} - {\rm P}_1 \end{equation*}

Therefore, the fraction of the free primer–template complex is reduced to

(3) \begin{equation*} \frac{{[{\rm P}_1 ]_{{\rm free}} }}{{[{\rm P}_1 ]}} = \frac{1}{{1 + K_{{\rm Inh}} [{\rm Inh]}}}\end{equation*}

and the effective first order rate constant for the disappearance of P_1_ is given by

(4) \begin{equation*} k_{{\rm eff}} = \frac{{k'[{\rm M]}}}{{1 + K_{{\rm Inh}} [{\rm Inh]}}} = \frac{k}{{1 + K_{{\rm Inh}} [{\rm Inh]}}}\end{equation*}

Taking the inverse,

(5) \begin{equation*} \frac{1}{{k_{{\rm eff}} }} = \frac{1}{k} + \frac{{K_{{\rm Inh}} }}{k}[{\rm Inh]}\end{equation*}

a linear relation between 1/*k*_eff_ and the inhibitor concentration is obtained. Plotting (1/*k*_eff_) against [Inh] should yield a straight line with a slope *K*_Inh_/*k* and an intercept (1/*k*). Dividing slope by intercept yields *K*_Inh_. For cases with a small number of data points and a strong inhibitory effect (at the highest concentration of the inhibitor), a large relative error in the intercept can result. Therefore, we used the actual experimental values of *k*′ [M] at [Inh] = 0 for such cases (see Supplementary Table S1 and Supplementary Figures S23–S29 in the Supplementary material).

**Figure 5.**
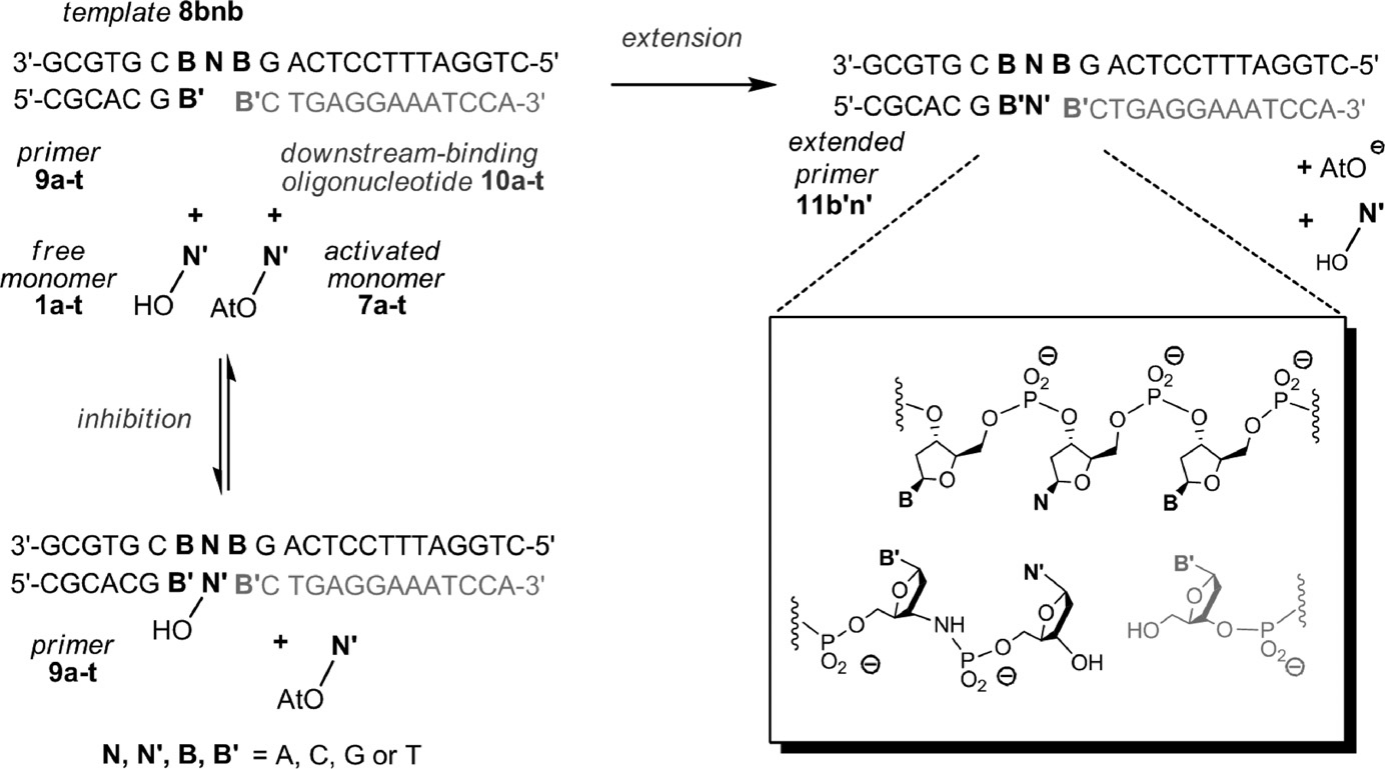
Oligonucleotide sequences and nucleotides for template-directed primer extension reaction in the presence or absence of an unactivated (free) deoxynucleotide as inhibitor. Assays at increasing concentrations of inhibitor were performed in the presence or the absence of a downstream-binding oligonucleotide that provides additional stacking interactions to the incoming nucleotide. Conditions: 3.6 or 7.2 mM monomer, 0–72 mM free nucleotide, primer extension buffer (200 mM HEPES, 80 mM MgCl_2_, 400 mM NaCl, pH 8.9), 20°C.

Figure 5 shows the components of the kinetic assays performed. We used oligodeoxynucleotide sequences and a primer with a 3′-terminal 3′-amino-2′,3′-dideoxynucleoside (40). Monomers were OAt-esters of 3′-deoxynucleotides (35). A total of 16 different sequence motifs were employed, four each for A/A, C/C, G/G, or T/T as neighboring bases to the four templating bases (A, C, G or T).

**Figure 6.**
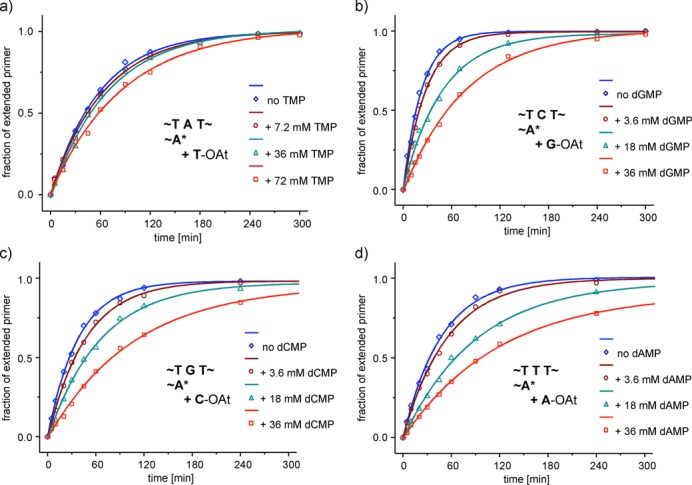
Binding of deoxynucleotides to primer–template complexes reveals itself through inhibition of primer extension. Kinetics of extension of primer 5′-CGCACGA-NH_2_-3′ (**8a**) by OAt-esters of deoxynucleotides as templated by T**N**T-type sequences, where the templating base **N** is A, C, G or T at increasing concentrations of unactivated dNMP (**1a–t**) as inhibitor, in the absence of a downstream-binding oligonucleotide at 20°C. Conditions: 36 μM primer, 3.6 or 7.2 mM dNMP-OAt (**7a–t**), 200 mM HEPES buffer, pH 8.9, 400 mM NaCl, 80 mM MgCl_2_. Symbols are experimental data and lines are monoexponential fits.

The rates of individual extension reactions were determined in assays monitored by MALDI-ToF MS (57), using conditions that allow for quantitative detection of oligonucleotides (58). Figure 6 shows a representative data set for each of the four nucleotides (A, C, G and T). Additional data can be found in the Supplementary data (Supplementary Figures S15–S21). Primer extension was measured at 20°C in the absence or the presence of a downstream-binding ‘helper’ oligonucleotide that provides additional stacking interactions (35), whose effect may be similar to that of nucleotides in template-directed polymerization (59). Five additional data sets were acquired in the absence of the downstream-binding strand at 10°C under conditions known to give near-quantitative conversion (40). The dissociation constants for the complexes of the free deoxynucleotides and the oligonucleotide duplexes are compiled in Table 2.

**Table 2.** Dissociation constants (*K*_d_′s) for nucleotides binding to template:primer complexes, as determined by inhibitor kinetics from global fits to primer extension data at increasing concentrations of free nucleotide inhibitors. The letter in the middle of the template sequence denotes the templating base

Entry	Nucleotides^a^ **7a-t/1a-t**	Template B^1^**N**B^2^	**8nnn**	dbo^b^	Temp. (°C)	*K*_d_^c^ (mM)
1	**T**	T**A**T	**8tat**	+	20	**59**
2	**G**	T**C**T	**8tct**	+	20	**2**
3	**C**	T**G**T	**8tgt**	+	20	**5**
4	**A**	T**T**T	**8ttt**	+	20	**5**
5	**T**	G**A**G	**8gag**	+	20	**83**
6	**G**	G**C**G	**8gcg**	+	20	**10**
7	**C**	G**G**G	**8ggg**	+	20	**18**
8	**A**	G**T**G	**8gtg**	+	20	**5**
9	**T**	C**A**C	**8cac**	+	20	**113**
10	**G**	C**C**C	**8ccc**	+	20	**7**
11	**C**	C**G**C	**8cgc**	+	20	**6**
12	**A**	C**T**C	**8ctc**	+	20	**7**
13	**T**	A**A**A	**8aaa**	+	20	**77**
14	**G**	A**C**A	**8aca**	+	20	**5**
15	**C**	A**G**A	**8aga**	+	20	**15**
16	**A**	A**T**A	**8ata**	+	20	**5**
17	**T**	T**A**T	**8tat**	−	20	**200**
18	**G**	T**C**T	**8tct**	−	20	**15**
19	**C**	T**G**T	**8tgt**	−	20	**19**
20	**A**	T**T**T	**8ttt**	−	20	**17**
21	**T**	C**A**C	**8cac**	−	10	**144**
22	**G**	C**C**C	**8ccc**	−	10	**5**
23	**C**	C**G**C	**8cgc**	−	10	**30**
24	**A**	C**T**C	**8ctc**	−	10	**7**
25	**C**	T**G**T	**8tgt**	−	10	**10**

^a^Monomer concentration: 3.6 mM for dAMP-OAt (7a), dCMP-OAt (7c) or dGMP-OAt (7g), and 7.2 mM for TMP-OAt (7t), and 0, 1, 5 or 10 eq. of unactivated monomer (1a–t).

^b^Downstream-binding oligonucleotide (dbo).

^c^Dissociation constant for deoxynucleotide, calculated from *K*_inh_ values.

The extension rate for the monomer reacting the fastest (dGMP-OAt on the template with TCT as core motif) gave a *t*_1/2_ of 2 min in the absence of any inhibitor. This value decreased by a factor of 20 in the presence of 10 eq. of dGMP (Supplementary Figures S31 and 32, Supplementary material), indicating strong binding. For the extension with dAMP-OAt, directed by the TTT template motif, the *t*_1/2_ increased from 7 to 60 min when adding 10 eq. of dAMP. There was also a significant effect of the neighboring nucleotides. When adenines were the neighbors of the incoming nucleotide (TNT templates, entries 1–4 of Table 2), binding was strongest. The absence of the downstream-binding strand led to an ∼3- to 4-fold increase in *K*_d_ at 20°C (entries 1–4 and 17–20, Table 2), whereas lowering the temperature from 20 to 10°C had a modest and less uniform effect on binding (see also Supplementary Figure S30 in the Supplementary data).

Overall, the dissociation constants found range from 2 mM for dGMP binding to a TCT template in the presence of a helper strand and 200 mM for TMP binding to a TAT template in the absence of a helper. They are thus close to the values found for hairpins by NMR (Table 1). The slowest reaction (incorporation of T on an A-template in the absence of a downstream-binding oligonucleotide) also gave the smallest inhibitory effect for the unactivated nucleotide (Figure 6a). Even in the presence of 10 eq. of unactivated TMP, only a modest slowdown of the extension was observed. In contrast, addition of 10 eq. of dNMP led to a very significant drop in rate for each of the remaining three bases (N = A, C or G; Figure 6b–d), confirming a significant occupation of the extension site by the monomers. Here, unlike in the hairpin case, the binding of dAMP and dCMP was rather similar to that of dGMP (Figure 6 and Table 2). Overall, binding strength decreases in the following order G>A≈C>>T.

**Figure 7.**
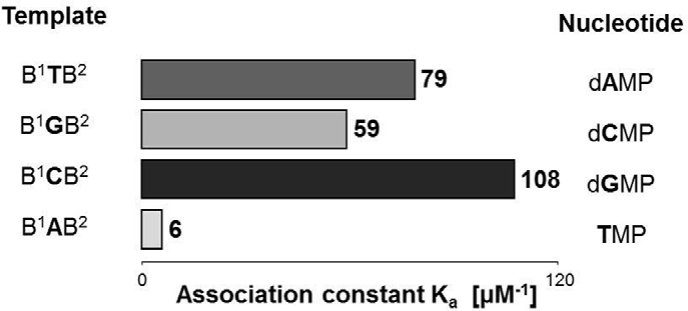
Association constants for dNMPs binding to primer–template duplexes displaying complementary base at 20°C, as obtained by averaging over values of NMR titrations and inhibitory studies (first nine entries of Table 1 and entries 1–20 of Table 2). See Tables 1 and 2 for conditions.

This order is also reflected in the global binding constants for the four different deoxynucleotides shown in Figure 7 that were calculated by averaging over the data at 20°C for each given base (Tables 1 and 2). The purines bind most strongly, followed by dCMP, and TMP, which binds ∼18-fold less strongly than guanine. This suggests that the combination of stacking interactions (strongest for the purines), number of hydrogen bonds and strength of secondary electrostatic interactions (60) governs binding strength, with the former providing a significant portion of the overall free energy of binding.

### Binding isotherms

**Figure 8.**
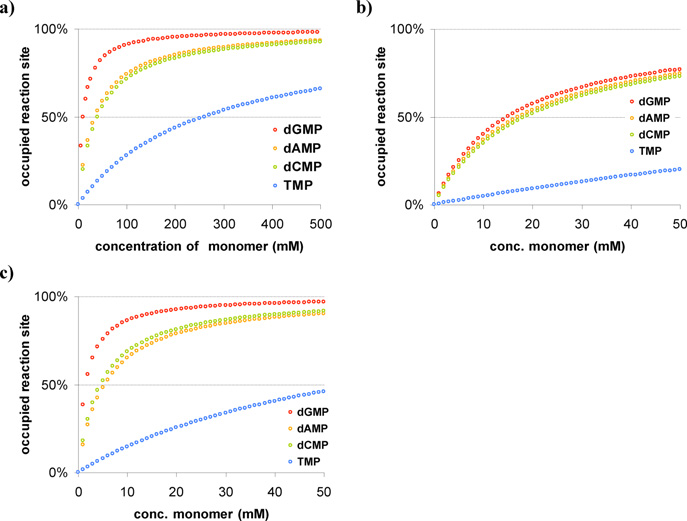
Occupancy of extension site by the deoxynucleotide complementary to the templating base at 20°C, as calculated for different concentrations of 2′-deoxynucleotides **1a–t** using binding constants reported in Table 1 or Table 2. Binding to (**a**) hairpins **2a**, **2c**, **2g** or **4t**, (**b**) template–primer duplexes **8tnt**:**9a–t** and (**c**) template–primer duplexes **8tnt**:**9a–t** in the presence of downstream-binding oligonucleotide **10a–t**. Note the different scales of the x-axes in (a) and (b)/(c).

With the binding constants in hand, we asked to what extent the reaction site of primer extension was occupied by the cognate nucleotide at a given concentration. Figure 8 shows calculated occupancies for different nucleotides and binding scenarios. When the reaction site is occupied, the primer terminus is protected from side reactions (38). An extension site occupied by the correctly paired monomer is also blocked from untemplated misincorporations. Untemplated reactions are common, and over-extension of primers, beyond the length of the template, are frequently observed (37,44). Finally, in the bound state, the activated nucleotide will be at least partially protected from side reactions with other nucleotides (unspecific polymerization, pyrophosphate formation, etc.) (61) and, being sterically less accessible, less prone to hydrolyze.

The binding isotherms shown in Figure 8 show that at 100 mM nucleotide concentration, only dGMP achieves near-quantitative occupancy of the primer extension site. For TMP, reaching a similar occupancy level would require unrealistically high concentrations. At the low millimolar concentrations typical for dNTPs in the cell, only basal binding occurs at the extension site in the absence of a helper oligonucleotide (or a polymerase). A neighboring nucleotide may provide a modest helper-like effect (59). Comparison of Figure 8b and c shows how a strongly binding neighbor can help with the incorporation of a weakly pairing monomer. It is known that one weakly pairing base in a sequence can become a ‘block’ for enzyme-free copying (62).

### Binding and rate of extension

**Figure 9.**
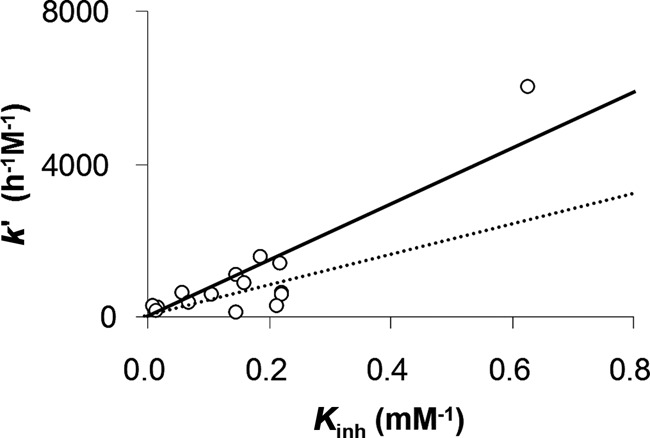
Correlation between dissociation constants and rates of extension (determined for 3.6 mM concentration of dAMP-OAt **7a**, dCMP-OAt **7c** and dGMP-OAt **7g**, or 7.2 mM concentration of TMP-OAt **7t** in the presence of downstream-binding oligonucleotides **10a–t** at 20°C) for the templating sequences listed in Table 2. The lines are linear functions obtained using regression analysis; solid black line, all 16 values (*r*^2^ = 0.775), broken line: highest data point excluded (*r*^2^ = 0.209).

Next, we asked how well binding correlates with rate. A strong correlation would suggest that the strength of the template effect is the dominant factor that determines whether enzyme-free primer extension occurs or not. Figure 9 shows a plot of rates versus binding constants for the 16 different template sequences studied (Table 2). It can be discerned that a loose correlation exists. Still the correlation is weak enough to suggest that other factors also play a role. Probably, the second step of the two-step mechanism proposed earlier for extension of amino-terminal primers (pseudorotation of a pentavalent intermediate; expulsion of the leaving group) (40) has a slightly different sequence dependence than the non-covalent binding equilibrium.

### Inhibition by spent monomers

**Figure 10.**
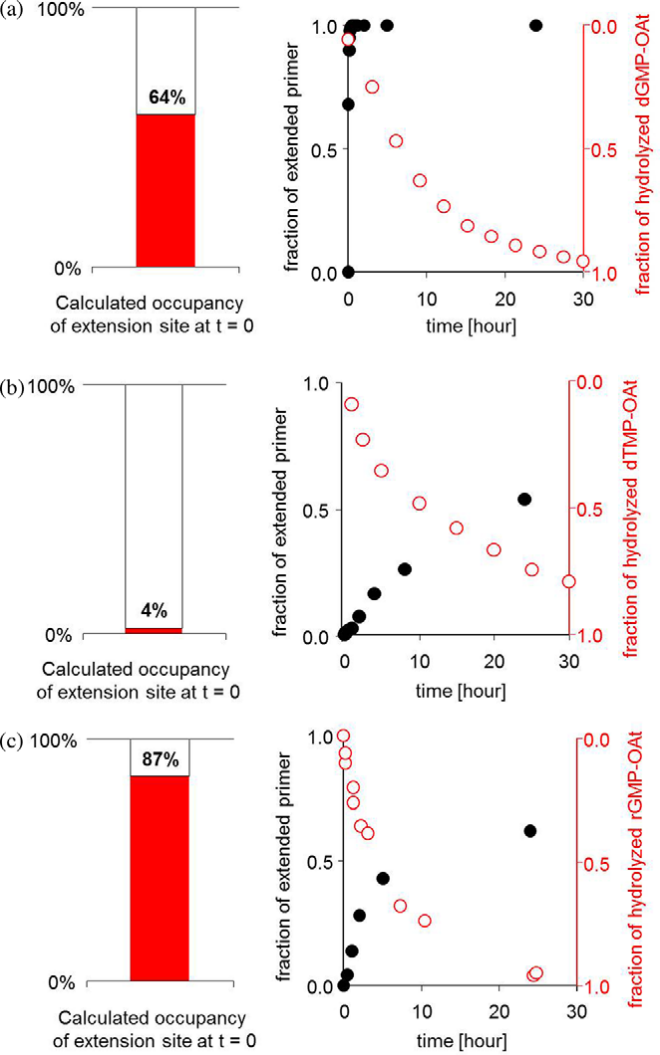
The role of inhibition in different chemical primer extension scenarios. On the right of each part, kinetics of primer extension (black dots) and monomer hydrolysis (open circles) are shown, and on the left, the calculated occupancy of the extension site by monomer or hydrolyzed monomer (inhibitor) at 0 min is shown as a bar graph. (**a**) Amino-terminal primer **9a** (36 μM), template **8tct**, helper strand **10a** and 3.6 mM dGMP-OAt (**7g**); (**b**) 3′-amino-terminal hairpin **2a** (36 μM) and 3.6 mM dTMP-OAt (**7t**); (**c**) RNA primer **13g** (100 μM), template **12ccg**, helper strand **14c** and 20 mM rGMP-OAt (**15g**). The *K*_d_ values used for calculating occupancies are 2 mM for dGMP, 260 mM for dTMP and 14 mM for GMP (Tables 1 and 2). See also the Supplementary Material (Supplementary Figures S15b, S22 and S33). Kinetics and hydrolysis data for the RNA case are from reference (29).

Since the binding constants for unactivated nucleotides are also inhibitory constants, the binding data provide a quantitative answer to the question of how important the inhibitory effect of spent monomers (nucleotides formed through hydrolysis of activate nucleotides) is for enzyme-free copying in different experimental scenarios. Figure 10 shows representative kinetics of primer extension and hydrolysis, together with the calculated occupancy of the extension site, assuming that monomer and spent monomer bind with the same affinity.

Figure 10a shows that for a highly reactive, amino-terminal primer and a monomer binding strongly, extension is so fast that the reaction is complete before hydrolysis produces a significant concentration of inhibitor. Figure 10b shows the case of a highly reactive, amino-terminus but a weakly pairing monomer (TMP-OAt **7t** binding to hairpin **2a**). Here, the extension is so slow that the formation of a significant concentration of spent monomer occurs before the reaction is over. But, the extent of binding of the inhibitor is minimal, so that its formation is inconsequential. Finally, Figure 10c shows the case of a less reactive RNA primer, combined with strongly binding GMP-OAt as monomer. Here, extension is so slow that hydrolysis can catch up with the desired copying process, so that the inhibitor formed does block extension significantly. (Perhaps, such an inhibitory effect can help to smooth out the differences in reaction rates between strongly and weakly pairing nucleotides, making it more likely that the weakly binding nucleotides compete successfully with the more strongly pairing ones.)

### Yield of primer extension

**Figure 11.**
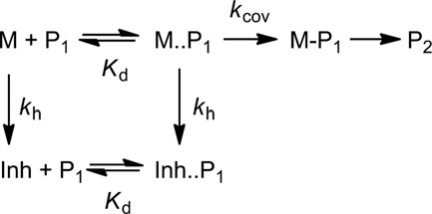
Binding equilibria and reactions for extension of a primer (P_1_) by an activated nucleotide as monomer (M). Hydrolysis of the monomer produces a free nucleotide that acts as an inhibitor (Inh). The non-covalent binding of both activated and free nucleotide to the primer are governed by the dissociation constant (*K*_d_), while the rates of extension (*k*_cov_) and hydrolysis (*k*_h_) govern the fate of the monomer. It is assumed that the primer is stably bound to the template.

Incomplete conversion is a key obstacle to enzyme-free replication of oligonucleotides (26). Therefore, we asked to what extent the yield of enzyme-free copying (and thus the perhaps most critical step of spontaneous replication, other than strand separation) can now be predicted, based on the binding constants and rates for the extension and the hydrolysis of monomers. First, we focused on an RNA-based system that is mechanistically simpler than those involving amino-terminal primers (40). All-RNA systems are at the focus of studies on prebiotic evolution (16,63). Figure 11 shows the model used for the simulations.

As shown in detail in the Supplementary material, the yield of product P_2_ can be calculated by Equation (6):

(6) \begin{equation*} {\rm Y}_{P\rm 2} = 1 - \exp \left[ {\frac{{k_{{\mathop{\rm cov}} } [{\rm M}]_0 (\exp [ - k_{\rm h} t] - 1)}}{{k_{\rm h} (K_{\rm d} + [{\rm M}]_0 + [{\rm Inh}])}}} \right] \end{equation*}

**Figure 12.**
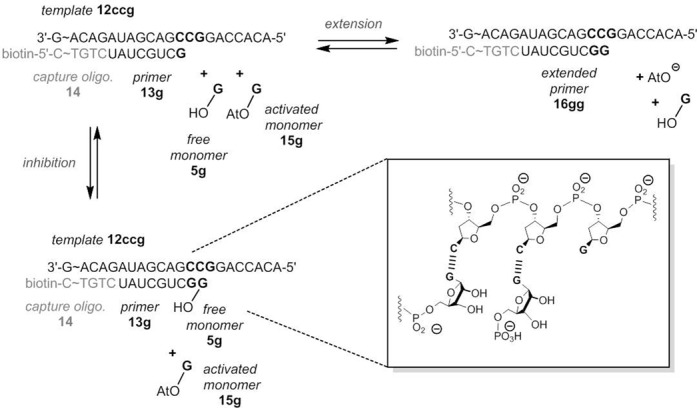
Oligonucleotide sequences and ribonucleotides for template-directed RNA primer extension reaction in the presence or absence of an unactivated (free) ribonucleotide as inhibitor, as described (29).

Yields of extension of RNA primers were calculated using the binding constant determined for GMP and RNA hairpin **5g** (14 mM, Table 1) and the data for extension and hydrolysis recently reported by Deck *et al.* for the sequence system shown in Figure 12 (29). Assuming that activated and unactivated nucleotide have the same affinity for the template and that the monomer binding equilibrium is reached rapidly, the occupation number is given by *α* = [M]_0_/(*K*_d_ + [M]_0_). Using the experimental *K*_d_ value of 14 mM (Table 1), *α* = 0.59 for assays performed at 20 mM monomer concentration.

**Figure 13.**
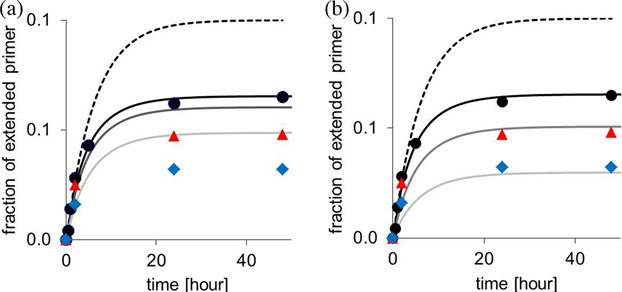
Simulated time-dependent yield of extended RNA primer (Figure 12) with and without hydrolysis and inhibition at initial concentrations of 20 mM monomer (GMP-OAt) and 268 μM primer. (**a**) Symbols represent published experimental values (29) for assays with different concentrations of inhibitor (GMP) added; circles: none, triangles 5 mM, diamonds 20 mM GMP. Solid lines are calculated using Equation (6). Values used for the simulation: *K*_d_ = 14 mM; *k_cov_* = 0.27 h^−1^, *k_h_* = 0.147 h^−1^. The dashed black line shows hypothetical kinetics without formation of inhibitor through hydrolysis. (**b**) Same as (a) but with a *K*_Inh_ value increased by a factor of 3, as expected for this system containing a downstream-binding oligonucleotide.

At early time points, the kinetics are not yet affected by hydrolysis of the monomer. In this case, the effective first order rate of the reaction can be determined using *k* = *αk*_cov_. A mono-exponential fit to the first four experimental data points shown in Figure 13a yields *k* = 0.16 h^−1^ leading to *k*_cov_ = 0.27 h^−1^. This value is similar to the rate of the extension with GMP-OAt on a slightly different template motif (UCU, where C is the templating base), which occurs with 0.38 h^−1^ under the same experimental conditions (29). Either value is close to the rate of hydrolysis under assay conditions (*k*_h_ = 0.15 h^−1^) (29), as expected for the ‘third scenario’ of Figure 10c.

Figure 13a shows plots of the simulated progress of primer extension during the first two days reaction time of the RNA extension and four different regimes. The first regime is that of a hypothetical extension without any inhibitory effect of hydrolyzed monomer (dashed black line). The second (solid black line) was calculated with the full model that takes concomitant formation of the inhibitor through hydrolysis into account. The third and the fourth case (dark gray and pale gray line, respectively) were calculated for extensions in the presence of 5 or 20 mM GMP (**5g**) (29).

For extension without addition of inhibitor, the calculated curves are close to the available experimental data (black circles in Figure 13), but the drop in yield induced by addition of the inhibitor is represented in a qualitative sense only. For the simulation of Figure 13a, the binding constant from the hairpin system (Figure 2) was used, even though the experimental data was from a full extension system with downstream-binding helper oligonucleotide (Figure 12). Helper oligonucleotides typically give a 3-fold decrease in *K*_d_, at least in the better studied DNA case (compare the first four entries of Table 2 with those in the lower part of the table where the same sequence motifs were assayed without helper). When the unactivated GMP was assigned a 3-fold lower *K*_d_ value than the activated monomer, a near-perfect agreement of calculated and experimental data was obtained (Figure 13b).

**Figure 14.**
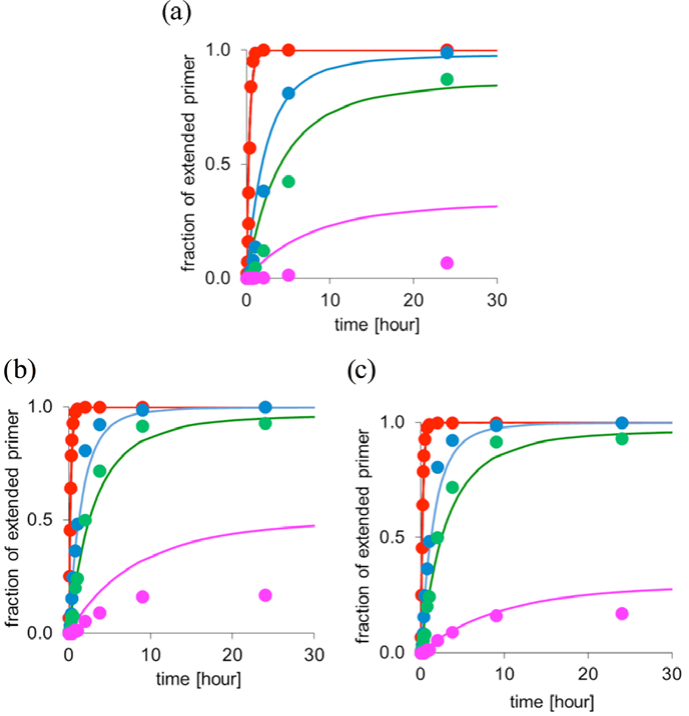
Simulated time-dependent yield of extension of amino-terminal DNA primer **9g** at decreasing concentration of monomer in the presence of downstream-binding oligonucleotide **10g**. Lines are calculated using Equation (6) with *k_cov_* values obtained from rate constants (assuming monoexpoential kinetics) and occupation numbers based on dissociation constants for nucleotides. Filled circles are experimental data for monomer concentrations of 3.6 mM (red), 0.36 mM (blue), 0.18 mM (green) or 0.036 mM (pink). (**a**) Monomer is A (dAMP-OAt) and template is **8ctc**; values of the simulation: *K*_d_ = 6.9 mM; *k_h_* = 0.109 h^−1^, *k_cov_ =* 8.3 h^−1^. (**b**) Monomer is G (dGMP-OAt) and template is **8ccc**; with values of *K*_d_ = 6.8 mM; *k_h_* = 0.093 h^−1^ and *k_cov_ =* 12.2 h^−1^. (**c**) Same as (b), except that a 2-fold lower monomer concentration was assumed for the pink data at the end of the dilution series (0.018 mM).

Finally, for assays with amino-terminal primers and DNA templates, the mechanistic situation is more complicated, and inhibition does not play a significant role. To test the limits of our theory, we simulated assays with decreasing concentration of the monomer, down to the micromolar range (10 eq. or even 1 eq. of activated monomer). This case is more challenging, as the *k*_cov_ values are more difficult to extract for biphasic kinetics, and because at so low a monomer concentration, side reactions from trace impurities, such as residual acetate, become more prevalent (38). Figure 14 shows the results for A and G as monomers.

It can be discerned that Equation (6) predicts the time- and concentration-dependent yields well for all but the very lowest concentrations of the monomers. Figure 14c shows that assuming a loss of reactive species, so that the effective concentration of the monomer is half of what is assumed in the ideal case, suffices to get a satisfactory agreement between theoretical and experimental data, even at the very lowest monomer concentration. Apparently, binding constants, global rate constants for the covalent step(s), and rate constants for hydrolysis of monomers largely suffice to explain incomplete conversion, even in this case.

## DISCUSSION

**Figure 15.**
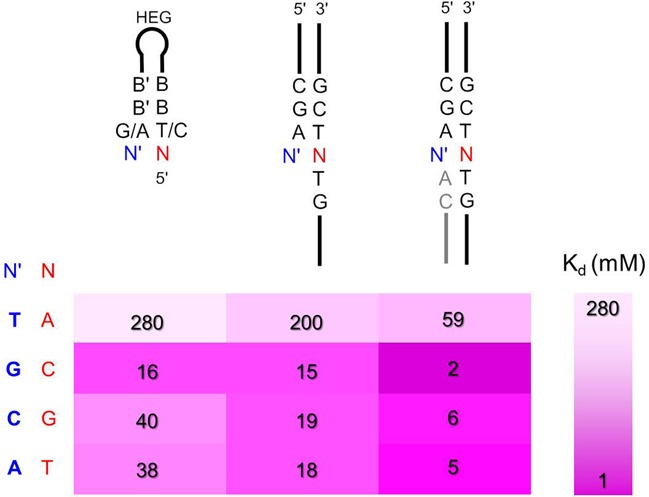
Effect of nucleobase and sequence context on binding of nucleotides to templating bases: heat map representation of representative dissociation constants for complexes between deoxynucleotides and termini of hairpins, primer-template complexes or primer–template complexes with downstream-binding oligonucleotide at 20°C. The color bar on the right-hand side is a graphical definition of how color intensity codes for binding strength. Data are from Tables 1 and 2.

Our results show that the dissociation constants for nucleotide–primer/template complexes are in the millimolar range. They are generally weaker than previously thought (51). The sequence dependence shows similarities to that found in the study on the rates of chemical primer extension (40). A large difference in binding strength is found between thymine and the other three bases, amounting to approximately one order of magnitude difference in dissociation or binding constants. Thymidine monophosphate is the only base for which near-saturation of the extension site is unrealistic at room temperature, even at concentrations approaching the physical limit. Lowering the temperature to 10°C does not eliminate this problem, nor does the presence of a downstream-binding oligonucleotide, though it does have a significant effect (Figure 15). Lowering the temperature further can be expected to tighten binding, and thus to improve yields (28).

Even in the best of all templating environments, with two large, hydrophobic adenine bases as neighbors (at the primer terminus and the 5′-end of the helper strand), the *K*_d_ value for TMP remains as high as 59 mM (first entry, Table 2). Having purines as stacking partners at the neighboring positions helps all incoming deoxynucleotides, but the overall effect of binding in a ‘TNT’ template region or binding to a less favorable ‘GNG’ sequence, with two cytosines as stacking partners, changes the stability of the complex by a factor of five at best, and as little as a factor of 1.4 in the case of dAMP. Being without any downstream interactions, as in the case of binding a base at the very terminus of a template/hairpin, leads to a decrease in binding that can be as large of 7-fold, as in the case of dCMP binding to hairpin **2g** (Table 1) when compared to the long template **8tgt** in the presence of a helper strand (Table 2). For the most tightly binding deoxynucleotide, dGMP, the dissociation constants found range from 2 mM on template **8tct** with helper and 16 mM for hairpin **3c**. (This concentration range is close to that of the intracellular concentration of nucleoside triphosphates (NTPs) found in present-day cells (64).)

The results of the exploratory experiments on mismatched binding provide a glimpse of the energetic consequence of mismatches. A G:T wobble base pair, most probably the most problematic of all mismatches (42,43) leads to binding that is just 10-fold weaker than that of the corresponding G:C combination (templates **4t** and **2c**, Table 1). Its *K*_d_ value is lower than that of the canonical T:A pairing (**1t**:**2a**), emphasizing how difficult it is to suppress this binding mode with natural T as templating base (65) at equimolar concentrations of all four monomers. On the other hand, no binding was detectable in the NMR titration for mismatched deoxynucleotide **1c** and hairpin **4t**, demonstrating that not all mismatches are problematic. Also, the pairing between GMP (**5g**) and hairpin **6c** consisting of RNA gave a *K*_d_ value of 14 mM, which is close to the values found for DNA hairpins **2c** and **3c**. This suggests that backbone structure has a minor effect on the strength of base pairing in this sequence context.

The nature of the primer (amino-terminal DNA versus RNA) does make a significant difference in terms of the importance of inhibition, though. As Figures 10 and 13 show, extension of an RNA primer, with slow-reacting natural backbone, suffers significantly from competitive inhibition by unactivated monomer, whereas the more reactive amino-terminal primer forming phosphoramidate linkages does not. This insight had eluded us and others, when kinetic data was available only.

Figure 8 allows one to gauge whether further increases in monomer concentration can be expected to improve yields for a given setting or whether this will be futile because near-saturation of the extension site has already been achieved. The data also helps to understand why submillimolar monomer concentrations require re-activation of spent monomers to achieve high yields in chemical primer extension (38).

Finally, the present data also allows a first glimpse at how the active site of polymerases improves binding of nucleoside phosphates. For example, the complex of dGTP and the phi29 DNAP polymerase was recently reported to form with a *K*_d_ of 1.4 μM (66), a value that is just three orders of magnitude smaller than some of the *K*_d_′s measured here for dGMP (and part of that increase in affinity is probably due to the interactions between the additional pyrophosphate and the Mg^2+^ ions in the active site).

## CONCLUSIONS

Our manuscript reports methodologies for measuring binding of nucleotides to templating bases, binding data and a model for calculating yields of chemical primer extensions. The model has been validated by simulating incomplete copying reactions for which experimental data are available. As Figures 13 and 14 show, we have also successfully separated binding from intrinsic reactivity in the active extension complex (the *k*_cov_ values show that amino-terminal primers are ∼30-fold more reactive than RNA primers). Our approach should also allow the quantitative prediction of the inhibitory effect of unactivated or spent monomers on enzyme-free primer extension for other chemistries and assay conditions, provided that binding constants and kinetic constants for extension and monomer hydrolysis are available. All three types of constants are accessible by straightforward experiments, using NMR (binding constant and hydrolysis) and extension assays monitored by gel electrophoresis or mass spectrometry.

Significant issues remain before enzyme-free replication of nucleic acids may be shown experimentally (67). Quantitative simulations of replication scenarios probably should include a matrix of fidelity values and quantitative data on the stalling after misincorporations (41). Further, it is interesting to ask how scenarios involving several monomers pairing simultaneously with a longer stretch of template affect reactivity, or how combinations of monomer extension and ligation, compared to the purely monomer-based regime known from present-day replication and transcription, perform. Efforts to tackle such systems are under way in our laboratories.

## SUPPLEMENTARY DATA

Supplementary Data are available at NAR Online, including protocols, NMR data, primer extension data, a more elaborate treatment of inhibitor kinetics, kinetics of the hydrolysis of monomers, and description of the model for predicting yields.

SUPPLEMENTARY DATA
