# Supplementary material for: The strength of the template effect attracting nucleotides to naked DNA
Source: Nucleic Acids Res. 2014 May 28;42(11):7409–20. doi: 10.1093/nar/gku314 (PMC4066754; doi:10.1093/nar/gku314)
Supplement: SUPPLEMENTARY DATA [file supp_42_11_7409__index.html]

SUPPLEMENTARY DATA 

# The strength of the template effect attracting nucleotides to naked DNA

## SUPPLEMENTARY DATA

**Files in this Data Supplement:**

- Supplementary Data
