# Supplementary material for: The strength of the template effect attracting nucleotides to naked DNA
Source: Nucleic Acids Res. 2014 May 28;42(11):7409–20. doi: 10.1093/nar/gku314 (PMC4066754; doi:10.1093/nar/gku314)
Supplement: SUPPLEMENTARY DATA [file supp_gku314_nar-00131-f-2014-File003.pdf]

Supplementary Data

for manuscript entitled

The Strength of the Template Effect Attracting Nucleotides to Naked DNA

by

Eric Kervio, Birgit Claasen, Ulrich Steiner, and Clemens Richert

Contents

1. General Information
2. Synthesis of Aminoterminal DNA Strands
3. NMR Experiments
4. Fitting NMR Data
5. Representative Data from NMR Titrations
6. Primer Extension Assays
7. Model for More Elaborate Treatment of Inhibitor Kinetics
8. Kinetic of the Hydrolysis of Activated Monomers
9. Model for Primer Extension Including Monomer Hydrolysis and Inhibition
10. References for Supporting Information

1. General Information

Reagents and Instrumentation. Sodium phosphate (dibasic and monobasic form), $MgCl_2$, NaCl, dAMP, dCMP, dGMP, rGMP and Dowex 50 WX8-200 cation exchange resin were purchased from Acros (Geel, Belgium), TMP and EDC·HCl were from Fluka (Deisenhofen, Germany). HOAt was purchased from TCI (Zwijndrecht, Belgium). Hairpin oligonucleotides featuring a 3'-terminal 3'-amino-2',3'-dideoxynucleoside were synthesized on controlled pore glass loaded with the 3'-terminal residues that had been prepared as previously described.[S 1] Phosphoramidites were from Chemgenes (Wilmington, MA), and all other reagents for DNA synthesis were from Proligo (Hamburg, Germany). Deuterated water (99.9%) was purchased from Euriso-Top (Saclay Gif/Yvette, France). The MALDI TOF mass spectra were acquired on Bruker REFLEX IV spectrometer, using software packages XACQ 4.0.4 and XTof 5.1. Oligonucleotides were measured in linear negative mode. A mixture of 2,4,6-trihydroxyacetophenone (0.3 M in EtOH) and diammoniumcitrate (0.1 M in H_2O) at a ratio of 2:1 (*v*:*v*) was used as MALDI matrix. Concentrations of stock solutions were determined by UV-Vis spectroscopy. NMR spectra were recorded on a Bruker Avance 500 spectrometer using a 5 mm BBO probe.

Activated Monomers. The oxyazabenzotriazolides (OAt esters) were synthesized via activation of dNMPs with EDC/HOAt, as previously described[S2] and were used without further modification.

2. Synthesis of Aminoterminal DNA Strands

Primers. Aminoterminal primers (**9a-t**)[S3] were prepared as follows. Oligonucleotides with 3'-terminal 3'-amino-2',3'-dideoxynucleoside were synthesized on 2 µmol scale via automated DNA synthesis, as described previously.[S1] The 3'-aminoterminal oligonucleotides were cleaved from the controlled pore glass and deprotected by treating the support with ammonium hydroxide (30%, 1 mL) at 55° C for 16 h. Solutions of crudes were purified by HPLC on C4 columns (250 x 10 mm, Nucleosil 120-5), using a gradient of CH_3CN in triethylammonium acetate (0.05 M, pH 7.0) at 50°C. Product-containing fractions were combined and lyophilized twice after redissolving in deionized water.

Analytical Data

5'-CGCACGA-3'-NH$_2$ (**9a**) MALDI-ToF MS calc. for $C_{67}H_{86}N_{30}O_{37}P_6$ 2089.4, found 2090.0;

5'-CGCACGC-3'-NH$_2$ (9c) MALDI-ToF MS calc. for $C_{66}H_{86}N_{28}O_{38}P_6$, 2065.4 found 2066.3;

5'-CGCACGG-3'-NH$_2$ (9g) MALDI-ToF MS calc. for $C_{67}H_{86}N_{30}O_{38}P_6$ 2105.4, found 2105.9;

5'-CGCACGT-3'-NH$_2$ (9t) MALDI-ToF MS calc. for $C_{67}H_{87}N_{27}O_{39}P_6$ 2080.4, found 2080.6.

Synthesis of Aminoterminal Hairpins

The synthesis of aminoterminal hairpins followed the same approach as the synthesis of the primers described above. The chain assembly used 1-*O*-dimethoxytrityl-hexaethylene glycol-12-*O*-(β-cyanoethyl-*N,N'*-diisopropyl) phosphoramidite, as shown in Figure S1, below.

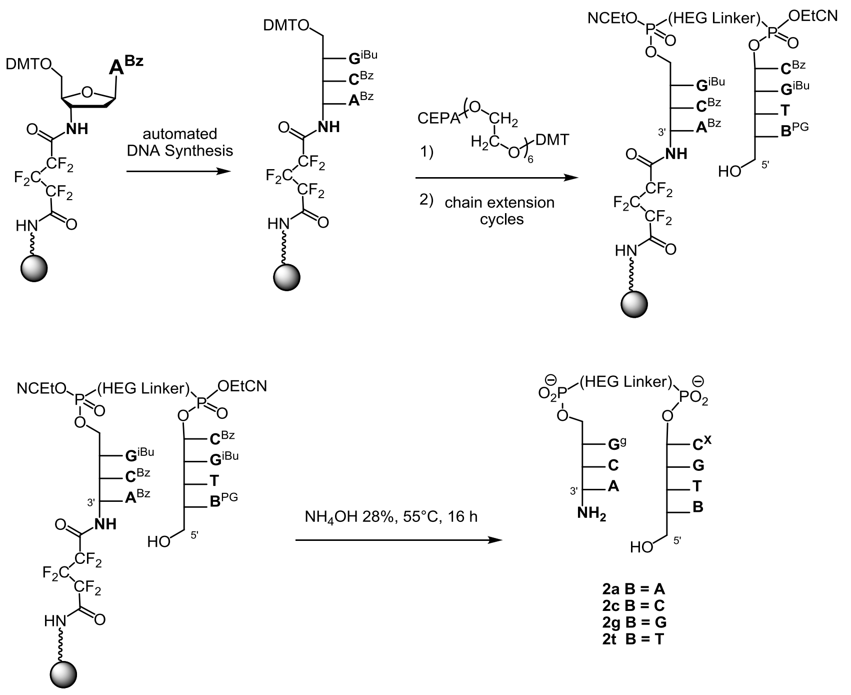

Figure S1. Syntheses of hairpin oligonucleotides, starting from a solid support featuring a 3'-amino-2',3'-dedeoxynucleoside and commercial cyanoethoxyphosphoramidates (CEPA) of deoxynucleosides or hexayethylene glycol.

Analytical Data for Hairpins

Yields are those obtained after HPLC purification.

5'-ATGC(HEG)GCA-3'-NH$_2$ (2a), Yield: 22%

MALDI-ToF MS calc. for $C_{80}H_{112}N_{29}O_{47}P_7$ 2448.7, found 2449.7;

5'-CTGC(HEG)GCA-3'-NH$_2$ (2c), Yield: 28%

MALDI-ToF MS calc. for $C_{79}H_{112}N_{27}O_{48}P_7$ 2423.7, found 2422.7;

5'-GTGC(HEG)GCA-3'-NH$_2$ (2g), Yield: 23%

MALDI-ToF MS calc. for $C_{80}H_{112}N_{29}O_{48}P_7$ 2464.7, found 2462.6;

5'-TTGC(HEG)GCA-3'-NH$_2$ (2t), Yield: 20%

MALDI-ToF MS calc. for $C_{80}H_{113}N_{26}O_{49}P_7$ 2439.7, found 2437.6;

5'-ACAG(HEG)CTG-3'-NH$_2$ (4a), Yield: 21%

MALDI-TOF MS calc. for $C_{80}H_{112}N_{29}O_{47}P_7$ 2448.7, found 2449.1;

5'-TCAG(HEG)CTG -3'-NH$_2$ (4t), Yield: 32%

MALDI-TOF MS calc. for $C_{80}H_{113}N_{26}O_{49}P_7$ 2439.7, found 2438.1.

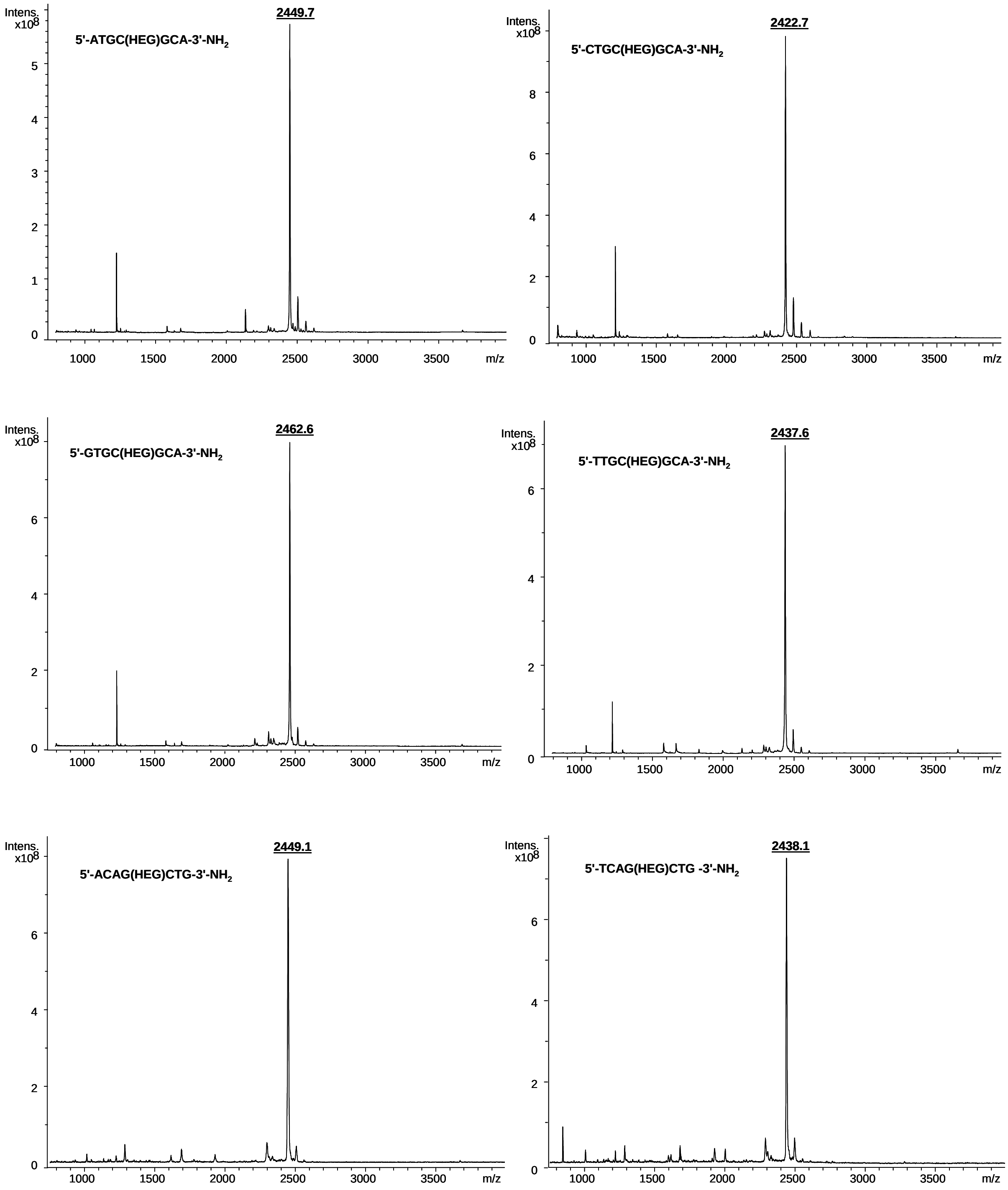

Figure S2. MALDI-TOF mass spectra of aminoprimers **2a**, **2c**, **2g**, **2t**, **4a**, and **4t**, after HPLC-purification. Labeled peaks are pseudomolecular ions ([M-H]$^-$, maxima of unresolved isotope patterns).

3. NMR Experiments

General procedure for the preparation of samples for NMR experiments. The purified fractions of each oligonucleotide were dissolved in an aqueous ammonium hydroxide solution (30%), excess ammonia was removed with a gentle flow of air, and the solution was frozen, followed by lyophilization. This was repeated at least twice. Then, the oligonucleotides were dissolved in 99.9% deuterated water, frozen and lyophilized at thrice. A buffer salt mixture (200 mM phosphate, 400 mM NaCl, 80 mM $MgCl_2$) was prepared to a pH of 8.9 in 99.9% deuterated water (pH uncorrected from deuterium effect), lyophilized twice, stored at -20 °C and used for preparing solutions prior to each experiment. A 1 to 1.5 mM solution of hairpin oligonucleotides was used for the temperature-dependent ^{1}H NMR and the 2D NMR experiments for peak assignment, and 0.5 mM solutions (2 mM for RNA) were used for the titration experiments.

General protocol for NMR experiments. NMR samples were prepared in 3 mm NMR tubes for Bruker MATCH™ holder (Bruker, Hilgenberg, Germany) to a final volume of 200 µL and a final oligonucleotide concentration of 0.5-2 mM in deuterated phosphate buffer, as described above. Two-dimensional spectra for signal assignment were acquired with 4k data points in f2 and 512 increments in f1 and a repetition delay of 2 sec. NOESY spectra were recorded with a mixing time of 250 ms and TOCSY-spectra were run with a spin-lock time of 60 ms. Suppression of the excess solvent peak was achieved by the application of a presaturation pulse during the recycle delay. Spectra processing was performed using Topspin 3.0 software (Bruker Biospin). Processing parameters: $\pi/2$ shifted sine^2 (QSINE, SSB 2) apodization was applied in both dimensions prior to Fourier transformation unless otherwise specified.

One-dimensional ^{1}H-NMR experiments were recorded with 32k data points and a repetition delay of 2 s. The data were processed using an exponential window function with a line broadening of 0.3 Hz prior to Fourier transformation. All spectra were calibrated to triethylamine at 1.21 ppm. For the temperature-dependent NMR experiments, the sample temperature was calibrated using either an ethylene glycol (high temperature) or methanol sample (low temperature).

3. NMR Experiments

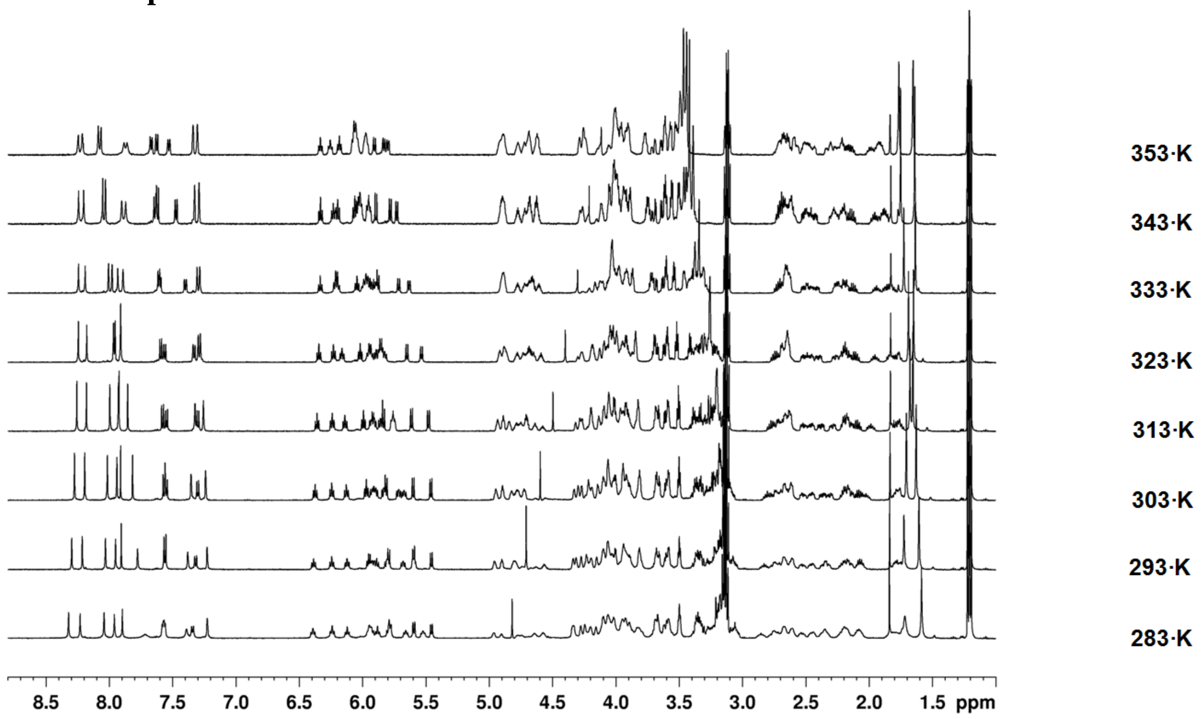

Figure S3. Temperature dependence of ^{1}H NMR spectrum (500 MHz) of hairpin oligonucleotide 5'-CT**A**TGC(HEG)GCA (**3a**) (1 mM) in deuterated phosphate buffer (200 mM, pH 8.9, 400 mM NaCl, 80 mM $MgCl_2$).

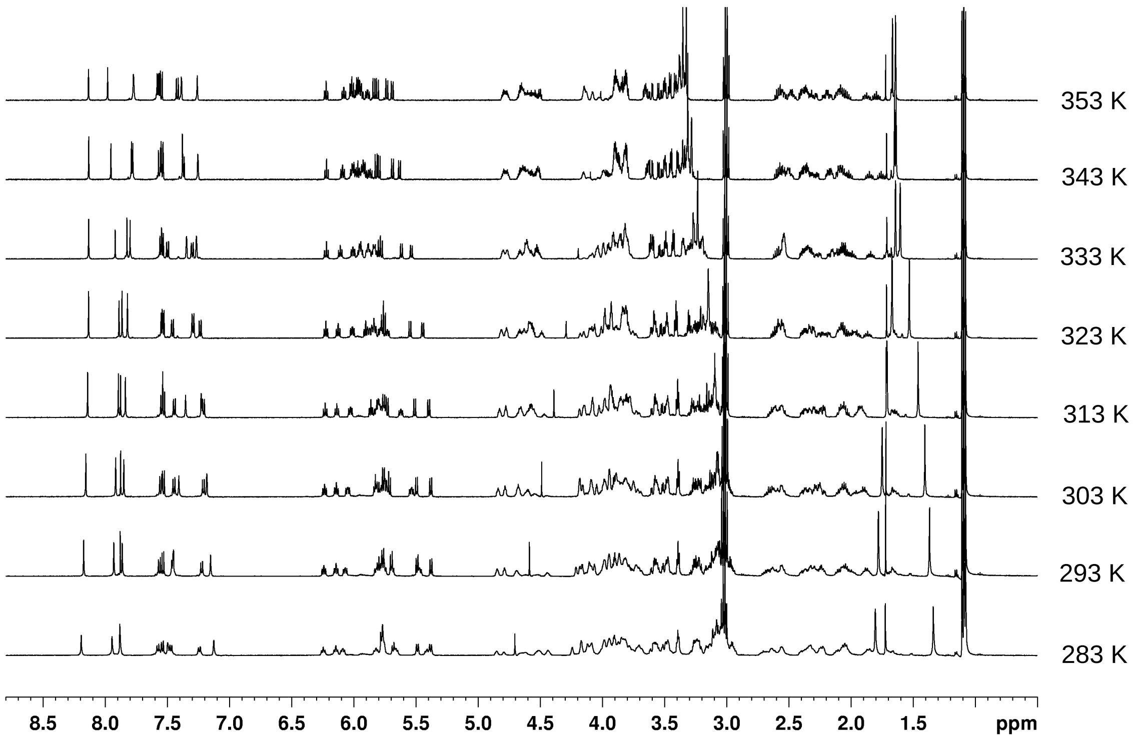

Figure S4. Temperature dependence of the ^{1}H NMR spectrum (500 MHz) of hairpin oligonucleotide 5'-CT**C**TGC(HEG)GCA (**3c**) (1 mM) in deuterated phosphate buffer (200 mM, pH 8.9, 400 mM NaCl, 80 mM $MgCl_2$).

a)

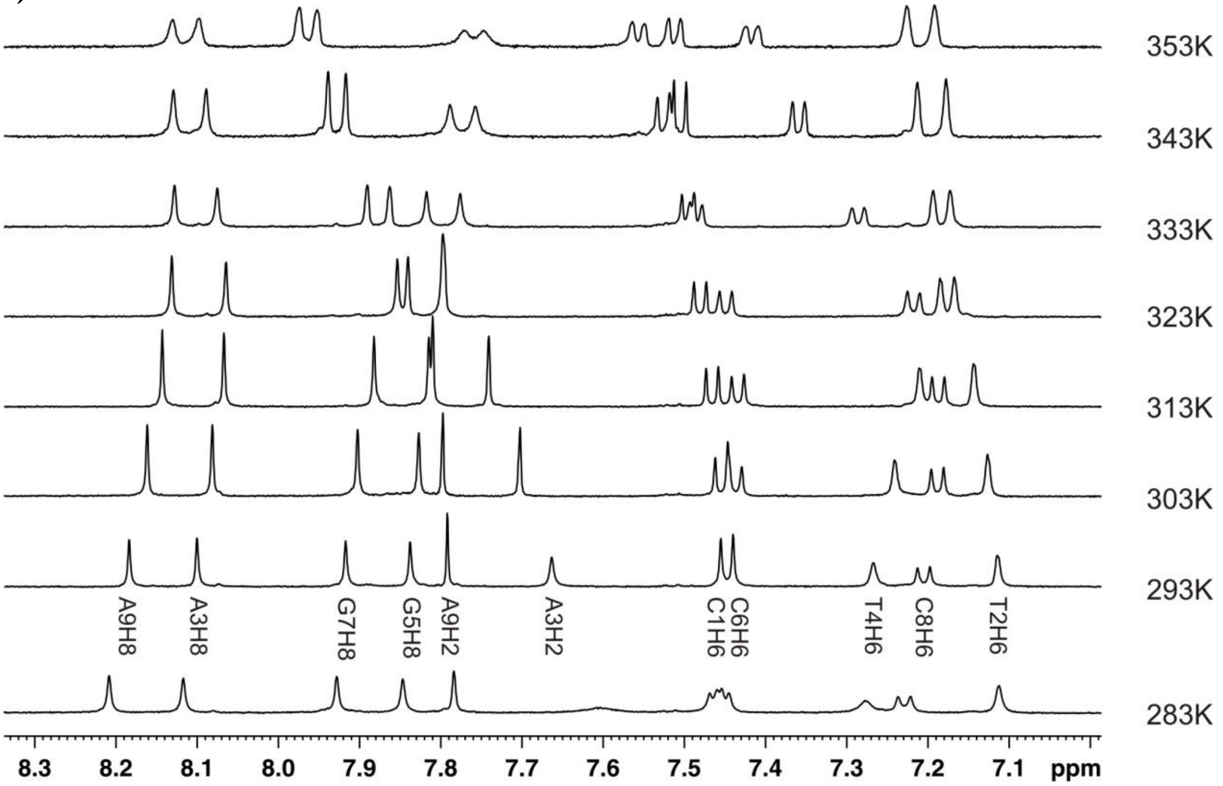

b)

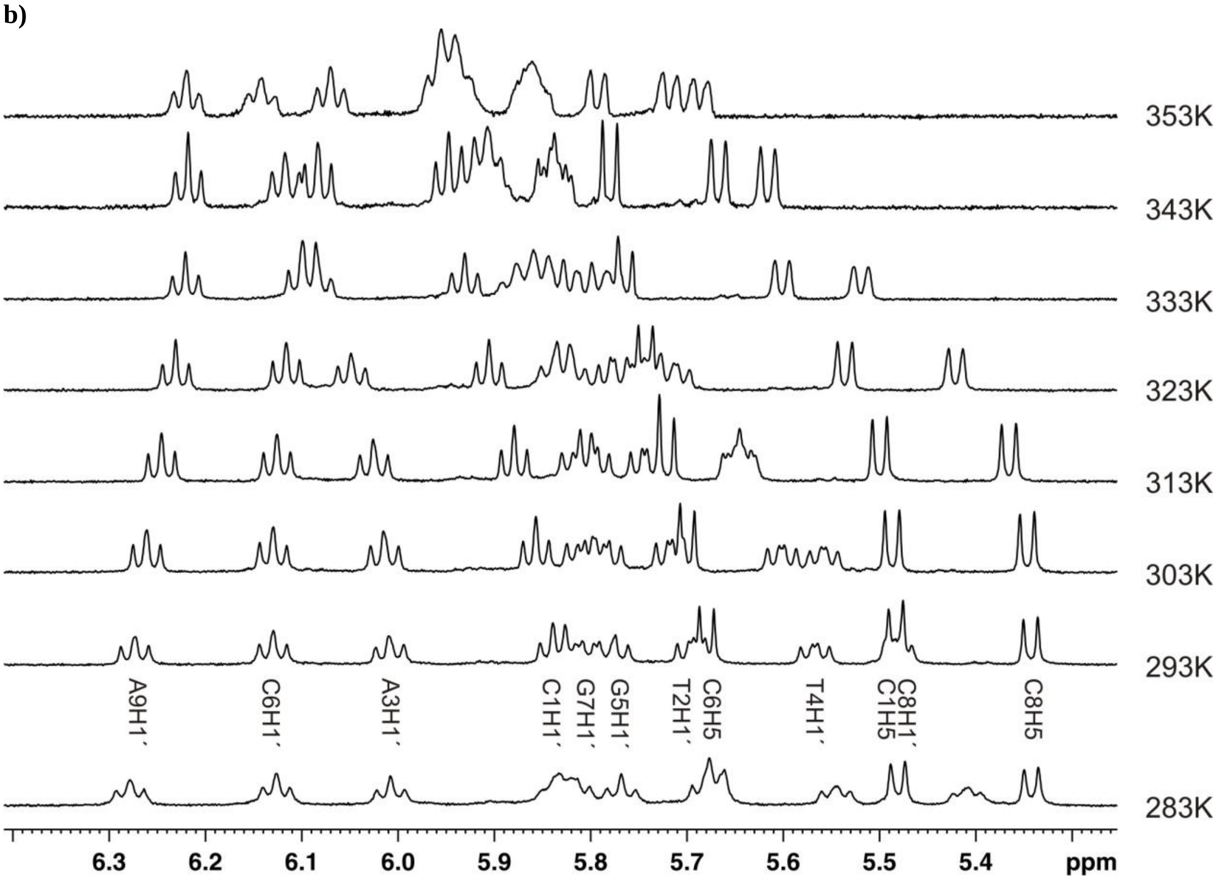

Figure S5. Expansion of the ^{1}H NMR spectrum (500 MHz) of hairpin 5'-CT**A**TGC(HEG)GCA (**3a**) at different temperatures, showing nucleobase resonances and resonances of H1' protons; same conditions as Figure S3/S4.

a)

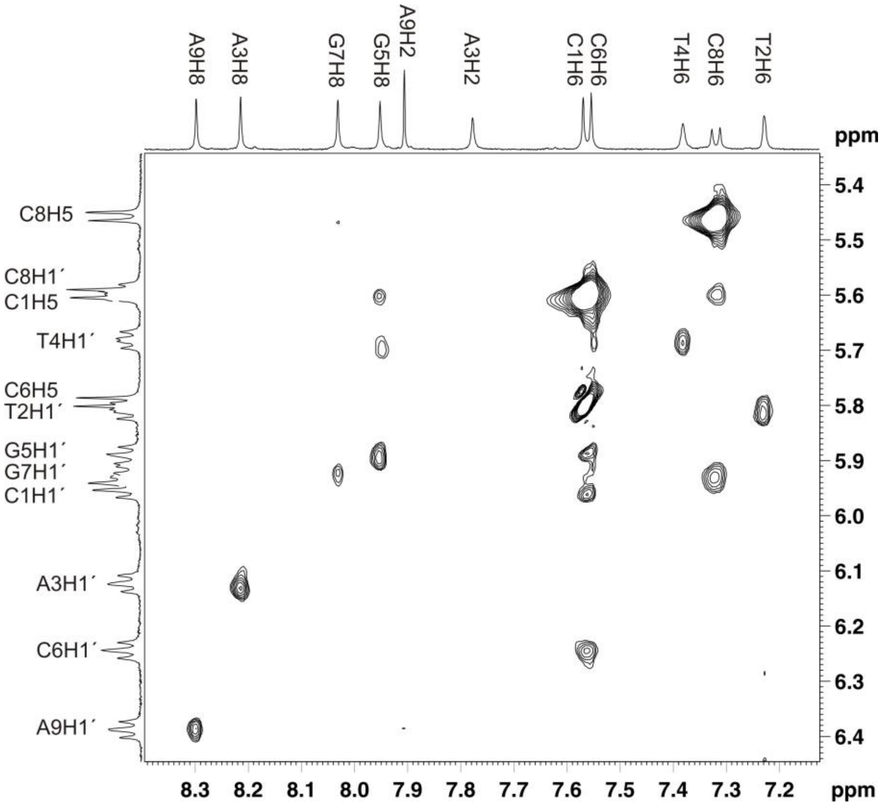

b)

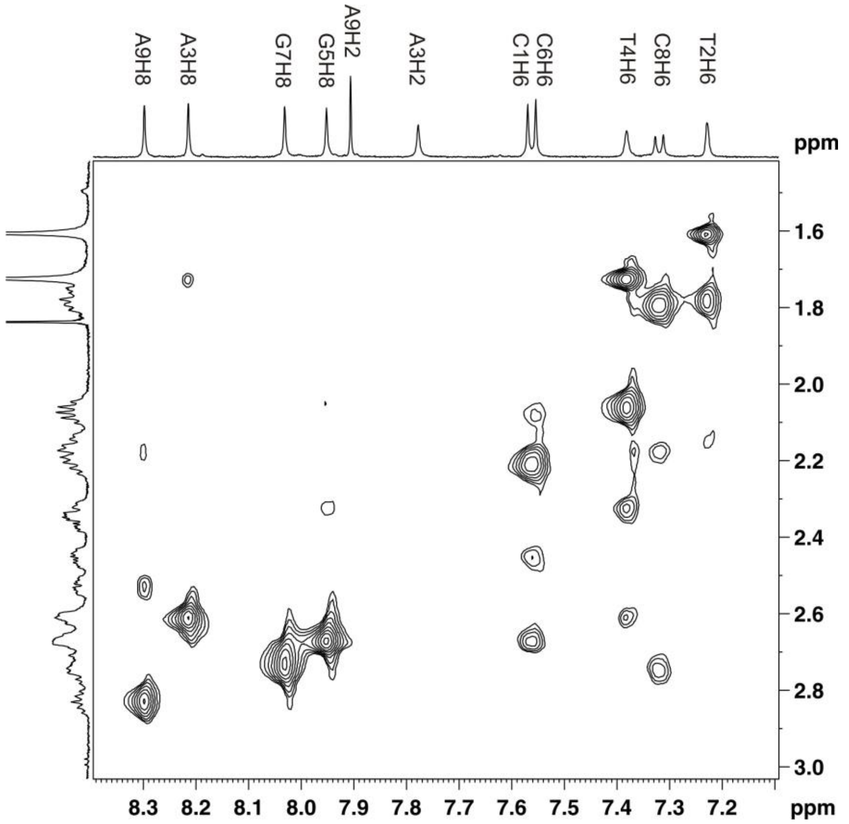

Figure S6. ^{1}H-NOESY spectra (500 MHz, 250 ms mixing time) of hairpin 5'-CT**A**TGC(HEG)GCA (**3a**) (1 mM) in deuterated phosphate buffer (200 mM, pH 8.9, 400 mM NaCl, 80 mM $MgCl_2$). a) Expansion showing cross peaks between nucleobase protons and H1' protons of 2'-deoxyriboses, b) Expansion showing cross peaks of nucleobases protons to H2' or H2'' protons of 2'-deoxyriboses.

a)

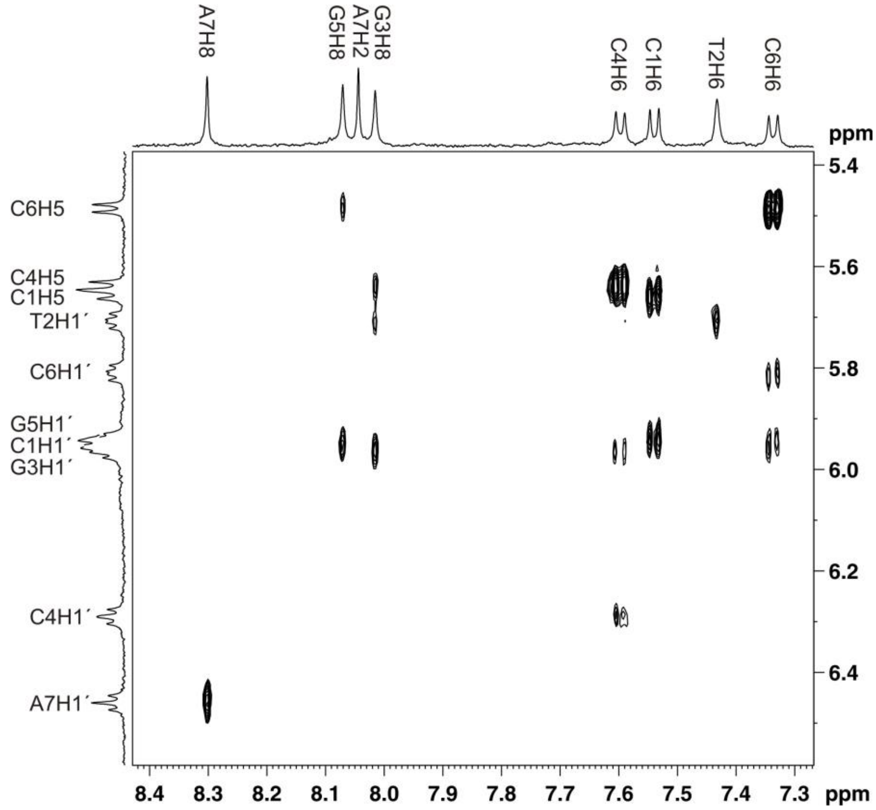

b)

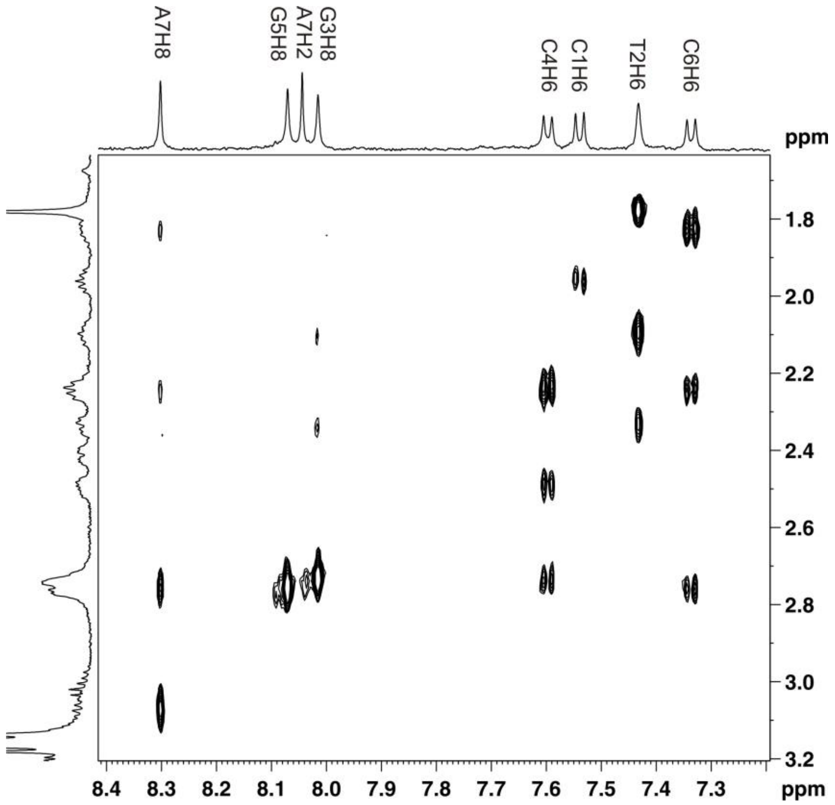

Figure S7. ^{1}H-NOESY spectra (500 MHz, 250 ms mixing time) of hairpin 5'-**C**TGC(HEG)GCA-3'-NH_2 (**2c**) (1 mM) in deuterated phosphate buffer (200 mM, pH 8.9, 400 mM NaCl, 80 mM $MgCl_2$). a) Expansion showing cross peaks between nucleobase protons and H1' protons of 2'-deoxyriboses, b) Expansion showing cross peaks of nucleobase protons to H2' or H2" protons of 2'-deoxyriboses.

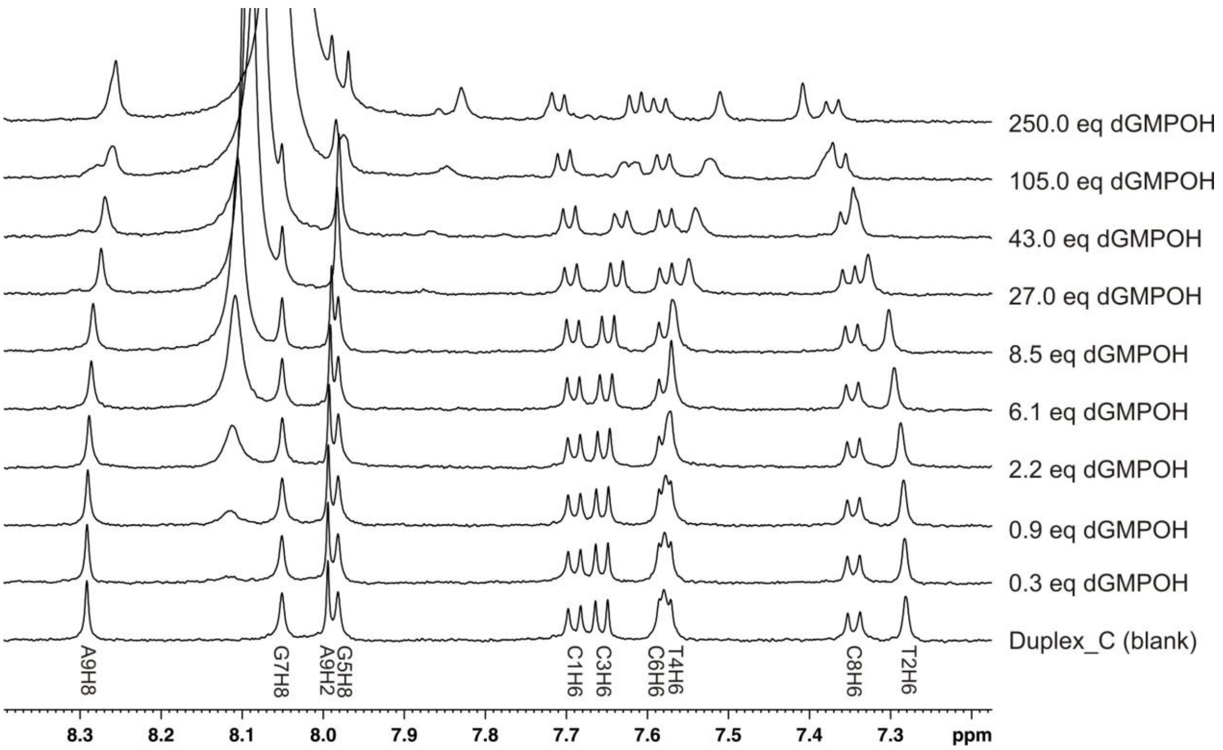

Figure S8. ^{1}H NMR spectra (500 MHz) from titration of 5'-CT**C**TGC(HEG)GCA (**3c**) with increasing equivalents of dGMP in deuterated phosphate buffer (200 mM, pH 8.9, 400 mM NaCl, 80 mM $MgCl_2$).

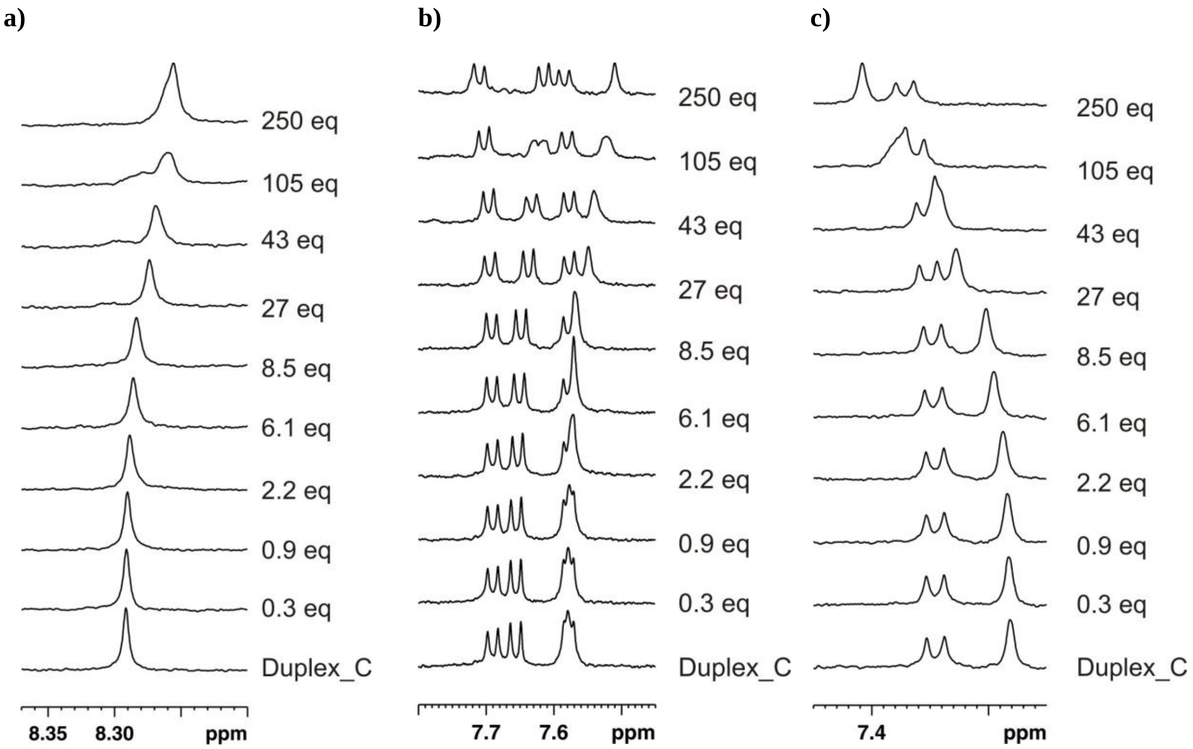

Figure S9. Expansions of selected nucleobase regions of spectra shown in Figure S8; a) H8 of adenine A9, b) H6 of cytosine C3, and c) H6 of thymidine T2.

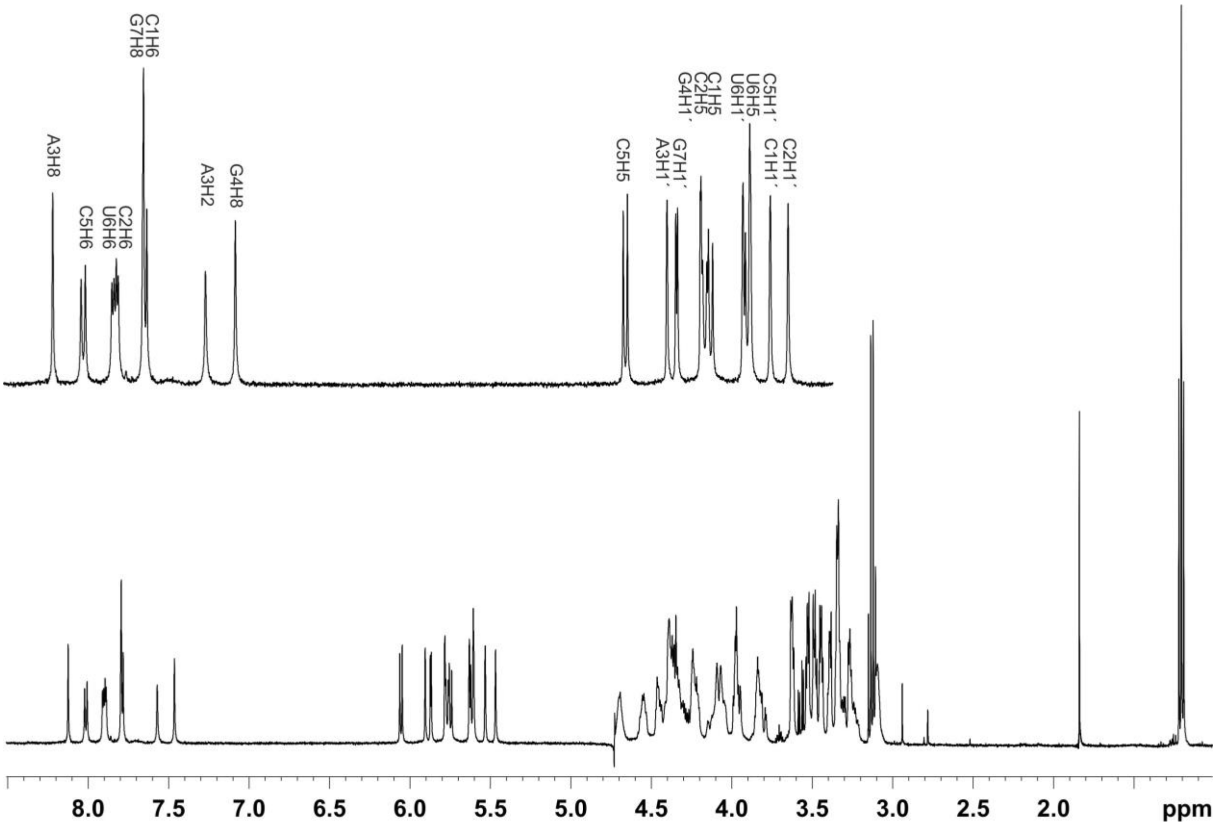

Figure S10. ^{1}H NMR spectrum (500 MHz) of RNA hairpin 5'-**C**CAG(HEG)CUG (**6c**) (2 mM) in deuterated phosphate buffer (200 mM, pH 7.0, 400 mM NaCl, 80 mM $MgCl_2$). The expansion in the upper part of the figure shows to the low-field region with labels for peak assignment.

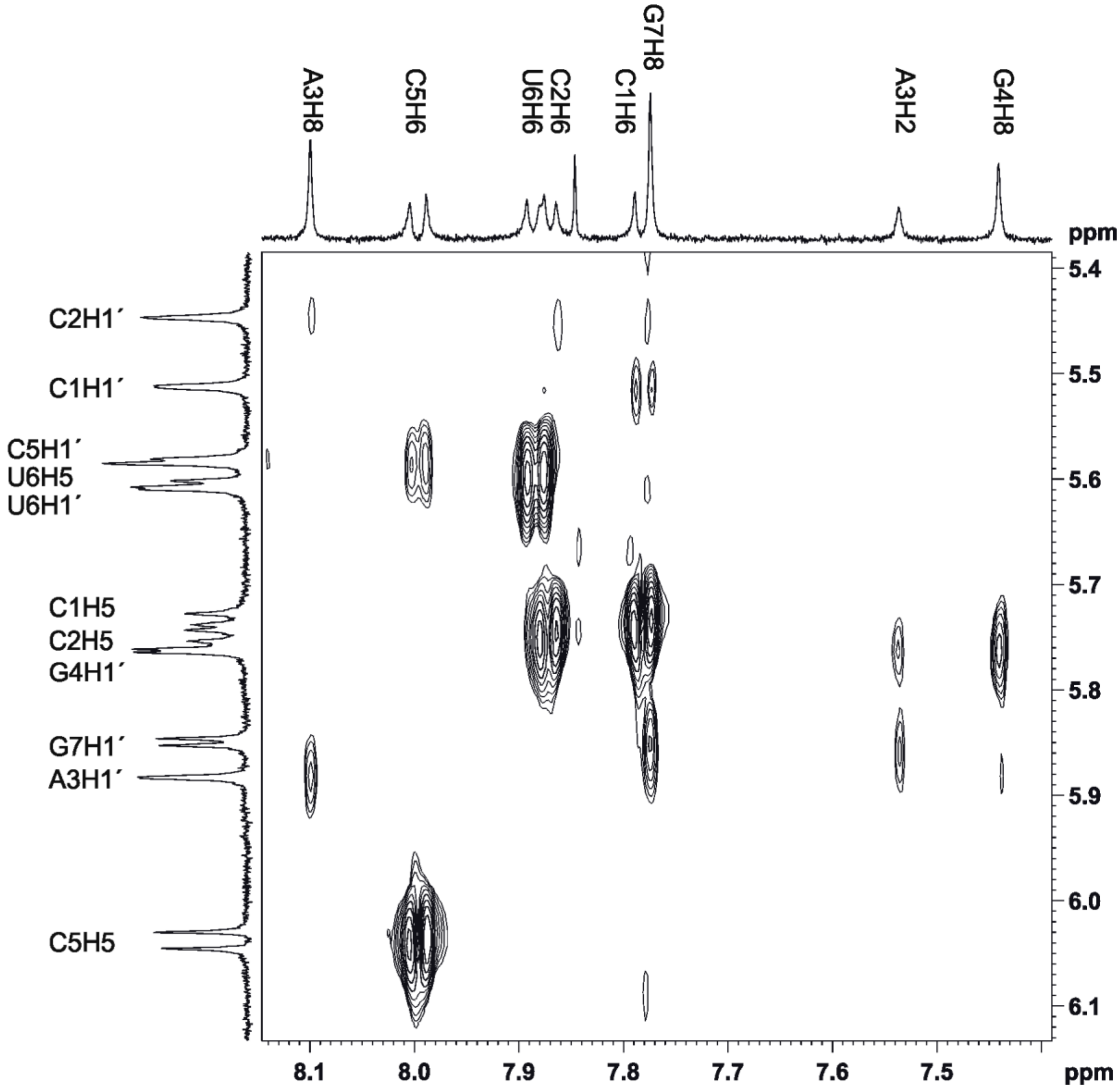

Figure S11. Expansion of NOESY spectrum (500 MHz, mixing time 250 ms) of RNA hairpin 5'-**C**CAG(HEG)CUG (**6c**) (2 mM) in deuterated phosphate buffer (200 mM, pH 7.0, 400 mM NaCl, 80 mM $MgCl_2$). Cross peaks are between nucleobase protons and H1' protons of riboses.

4. Fitting NMR Data

Determination of binding constant from NMR titration experiments. For titration experiments, stock solutions of 2'-deoxynucleosides 5'-monophosphates (**7a-t**) or guanosine 5'-monophosphate (**5g**) were prepared in deuterated NMR buffer, as described above. Solutions of different nucleotide concentrations were prepared to avoid strong dilution effects. Stock solutions were quantified by UV spectroscopy. Typically, titration experiments were performed at 293 K. To the solution of the hairpin oligonucleotide in deuterated phosphate buffer were added small volumes nucleotide solution. The resulting solution was centrifuged (2 min, 3000 rpm), and the ^{1}H NMR spectrum was acquired under the conditions given for ^{1}H-1D NMR experiments, above. Chemical shifts were determined graphically from the centers of peaks. Data analysis used the law of mass action for the binding equilibrium:

$$\mathrm{M} + \mathrm{P}_1 \underset{K_\mathrm{d}}{\overset{K_\mathrm{M}}{\rightleftarrows}} \mathrm{M..P}_1 \qquad \text{(S1)}$$

where the nucleotide is M, the unbound primer-template complex is P_1, and the complex of nucleotide and hairpin is M..P_1. The equilibrium mole fractions X_{P_1} and $X_{\mathrm{M..P_1}}$ of uncomplexed and complexed hairpin are related to the observed chemical shift δ by the equation

$$\delta = X_{\mathrm{P1}}\delta_{P1} + X_{\mathrm{M..P}}\delta_{M..P1} \qquad \text{(S2)}$$

Combining equation (S_2) with the law of mass action, and taking into account that $[M]_0 >> [P]_0$, the Hanna-Ashbaugh[S4] equation for a 1:1 mixture is obtained:

$$\Delta\delta = \frac{[M]_0 \cdot K_\mathrm{M}}{1+[M]_0} \cdot \Delta\delta_{\mathrm{M..P1}} \qquad \text{(S3)}$$

or

$$\Delta\delta = \frac{\Delta\delta_{M..P_1}}{K_\mathrm{d}} \cdot \frac{[M]_0}{1+\frac{[M]_0}{K_\mathrm{d}}} \qquad \text{(S4)}$$

where $\Delta\delta = (\delta_{\mathrm{P_1}} - \delta)$, $\Delta\delta_{\mathrm{M..P_1}} = (\delta_{\mathrm{P_1}} - \delta_{\mathrm{M..P_1}})$, and $[M]_0$ is the known total concentration of nucleotide. Fitting the experimental data points in a $\Delta\delta$ vs. $[M]_0$ diagram (curves are shown in Figures S7, S8, S9), the parameters K_d (listed in Table 1) and $\Delta\delta_{M..P_1}$ (see legend to Figures S8 and S9) were then obtained.

5. Representative Data from NMR Titrations

a)

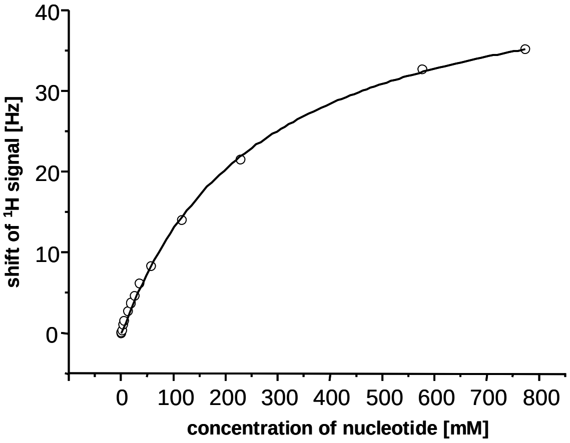

b)

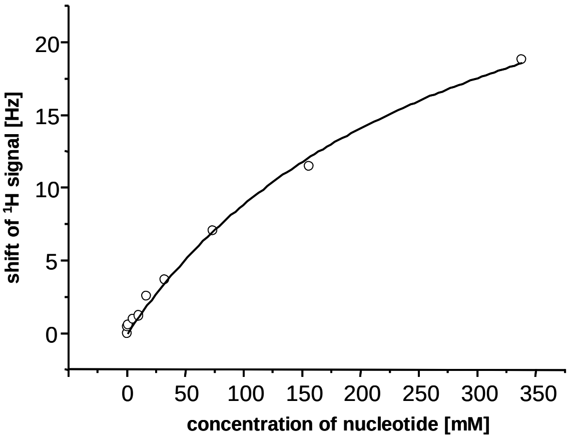

c)

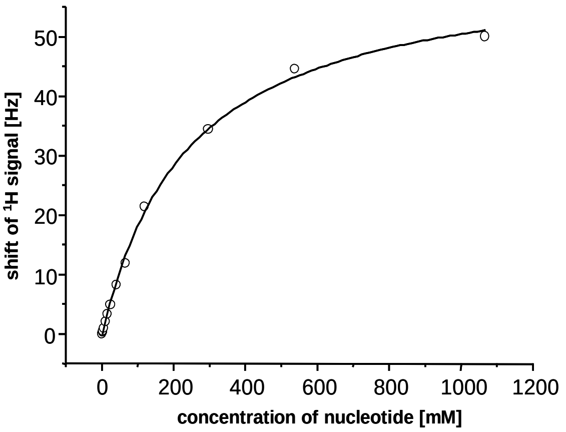

d)

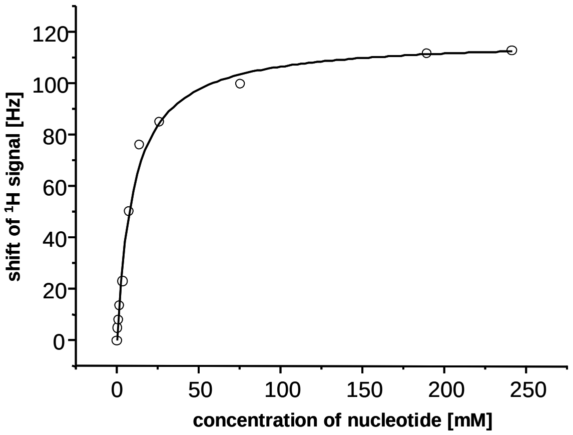

Figure S12. Plots of NMR titration data for:

a) A1H8 signal of sequence 5'-**A**TGC(HEG)GCA (**2a**) versus concentration of TMP (**1t**) (K_d = 261 mM ± 47) ($\Delta\delta$max = 47.1 Hz; r^2 = 0.999);

b) A3H2 signal of sequence 5'-CT**A**TGC(HEG)GCA (**3a**) versus concentration of TMP (**1t**) (K_d = 284 mM ± 44) ($\Delta\delta$max = 34.2 Hz; r^2 = 0.994);

c) A1H2 signal of sequence 5'-**A**CAG(HEG)CTG (**4a**) versus concentration of TMP (**1t**) (K_d = 241 mM ± 12) ($\Delta\delta$max = 62.7 Hz; r^2 = 0.998);

d) C1H6 signal of sequence 5'-**C**TGC(HEG)GCA (**2c**) versus concentration of dGMP (**1g**) (K_d = 9.9 mM ± 0.9) ($\Delta\delta$max = 117.2 Hz; r^2 = 0.991).

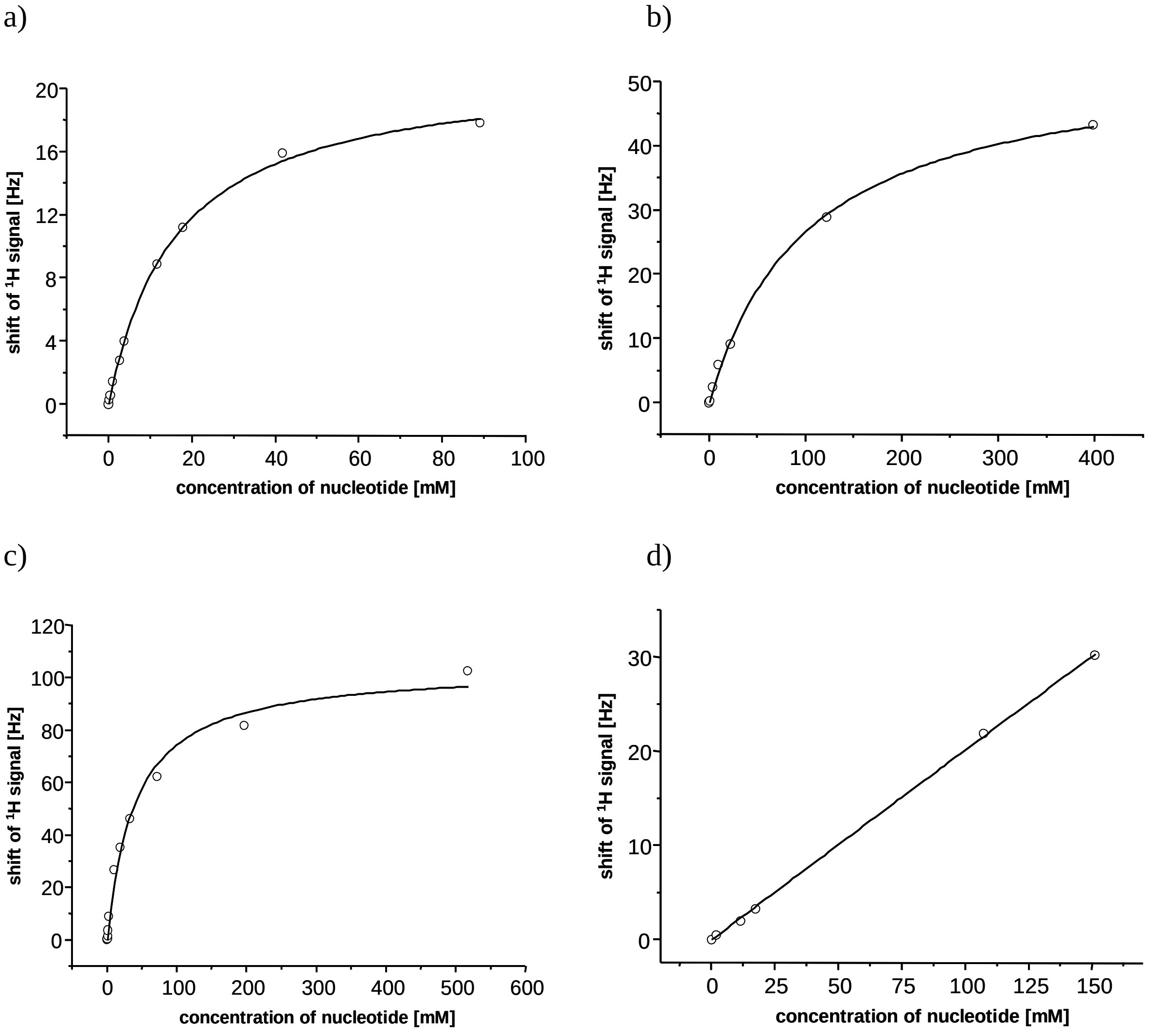

Figure S13. Plots of NMR titration data for:

a) C3H6 signal of sequence 5'-CT**C**TGC(HEG)GCA (**3c**) versus concentration of dGMP (**1g**) (K_d = 16 mM ± 0.8) (Δδmax = 21.4 Hz; r^2 = 0.999);

b) T1H6 signal of sequence 5'- **T**CAG(HEG)CTG (**4t**) versus concentration of dGMP (**1g**) (K_d = 103 mM ± 9) (Δδmax = 54.2 Hz; r^2 = 0.998);

c) G1H8 signal of sequence 5'- **G**TGC(HEG)GCA (**2g**) versus concentration of dCMP (**1c**) (K_d = 40 mM ± 5) (Δδmax = 104.1 Hz; r^2 = 0.988);

d) T1H6 signal of sequence 5'- **T**CAG(HEG)CTG (**4t**) versus concentration of dCMP (**1c**) (K_d > 500 mM) (Δδmax > 2000 Hz; r^2 = 0.999).

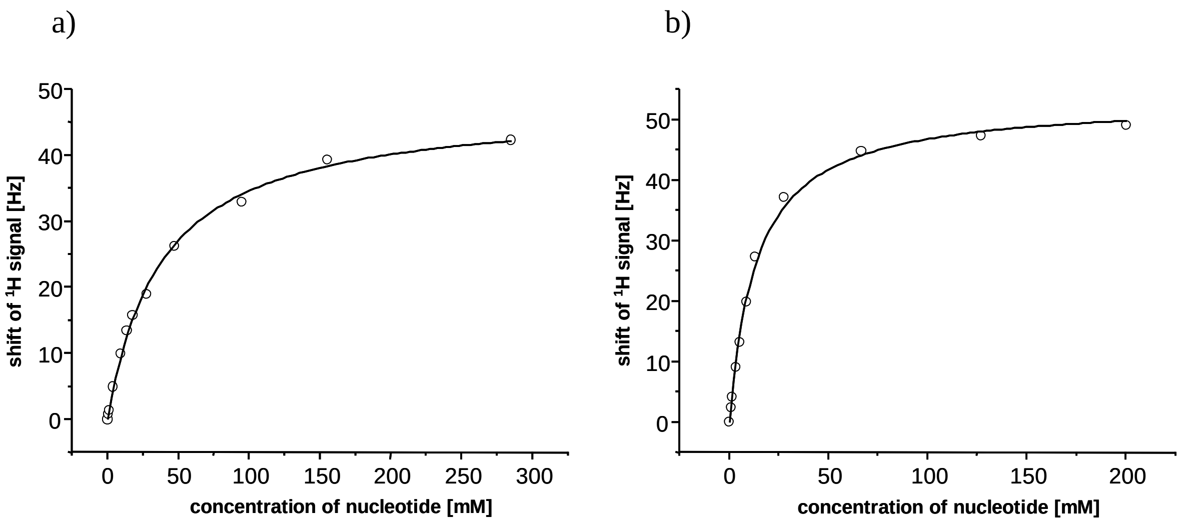

Figure S14. Plots of NMR titration data for:

a) T1H6 signal of sequence 5'- **T**CAG(HEG)CTG (**4t**) versus concentration of dAMP (**1a**) (K_d = 38 mM ± 2) (Δδmax = 47.8 Hz; r^2 = 0.997);

b) C1H6 signal of sequence 5'- **C**CAG(HEG)CUG (**6c**) versus concentration of rGMP (**5g**) (K_d = 13.7 mM ± 0.9) (Δδmax = 53.2 Hz; r^2 = 0.996)

6. Primer Extension Assays

Assays. As detailed in the Experimental Part, primer extension assays were performed on a 10 µL scale, using a slight modification of the protocol reported earlier.[S3]

Kinetic Analysis. A pseudo first order kinetic model was used because the concentration of the activated nucleotides is much higher than the concentration of the primers. The complex of oligonucleotides (primer/template/downstream-binding strand where appropriate) was treated as one reactant, and the activated nucleotides as the other. Monoexponential fits to kinetic data from assays were performed in *Origin Pro 8.0*, using $f(t) = Y_0 (1 - \exp(-kt))$, where Y_0 is a pre-exponential factor, used to determine the maximum extent of primer conversion, and k is the rate constant. Half-life times were calculated from rate constants using $t_{1/2} = t_h = \ln(2) / k$. Second order rate constants (k') were obtained by dividing k through the concentration of the monomer.

Kinetic Data

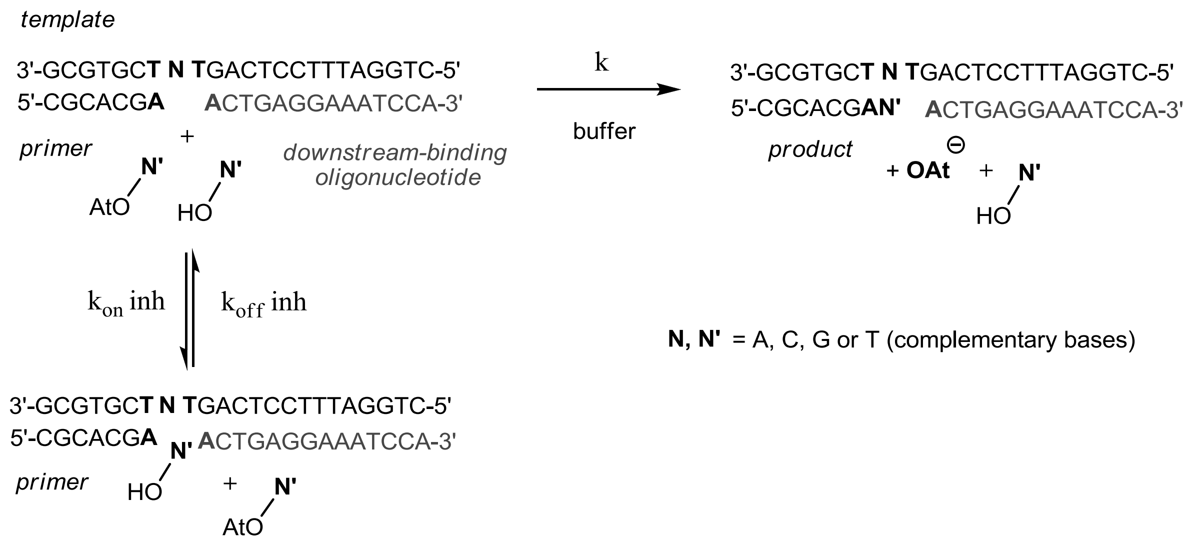

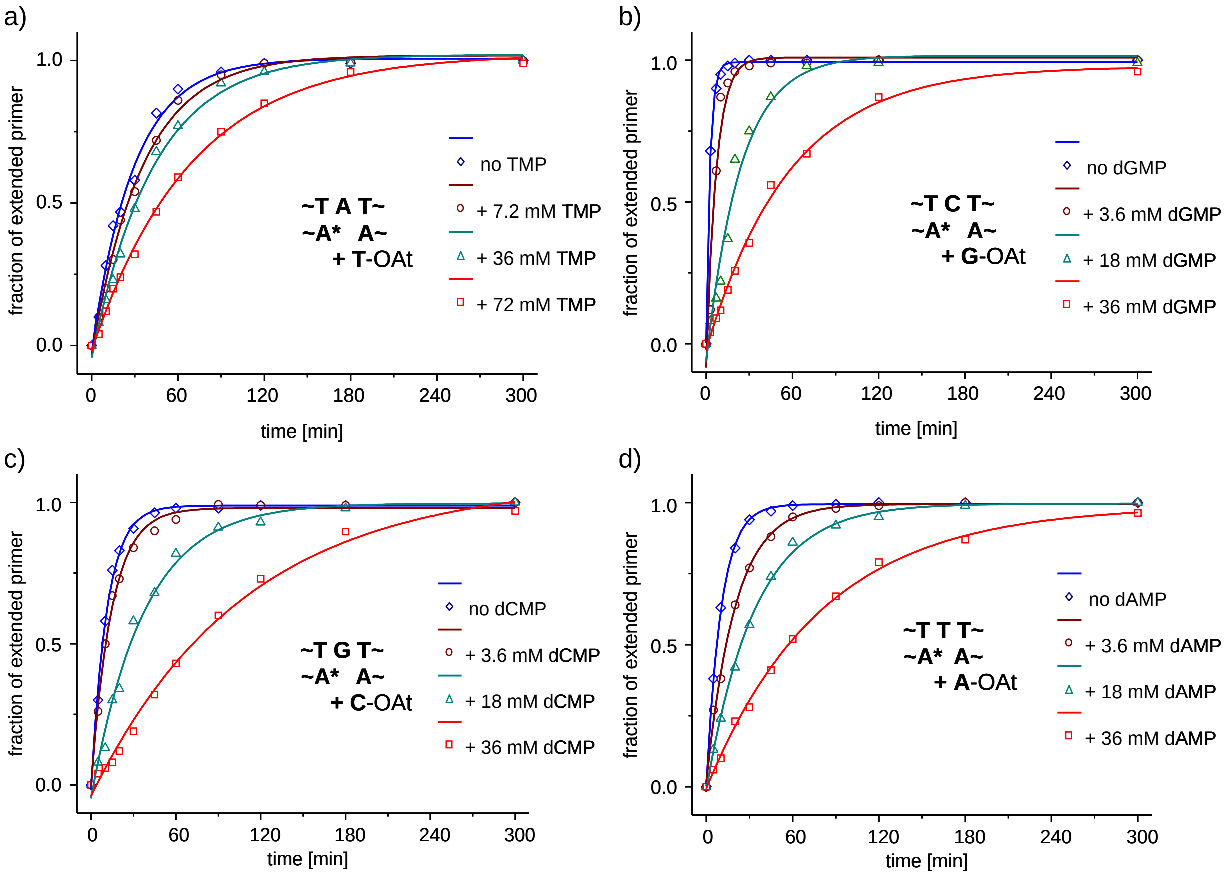

Figure S15. Kinetics of the extension of primer 5'-CGCACGA**A**-3'-NH_2 (**9a**, 36 µM) directed by TNT-type templates (**8tnt**, 54 µM) with downstream-binding oligonucleotide (**10a**, 54 µM) at 20°C, using 3.6 mM dN'MP-OAt (for **7a**, **7c** or **7g**) or 7.2 mM TMP-OAt (**7t**) and various concentrations of corresponding inhibitor dNMP's (**1a**, **1c**, **1g**, or **1t**) in HEPES buffer (200 mM, pH 8.9, 400 mM NaCl, 80 mM $MgCl_2$). Symbols are experimental data, lines are fits from the exponential model.

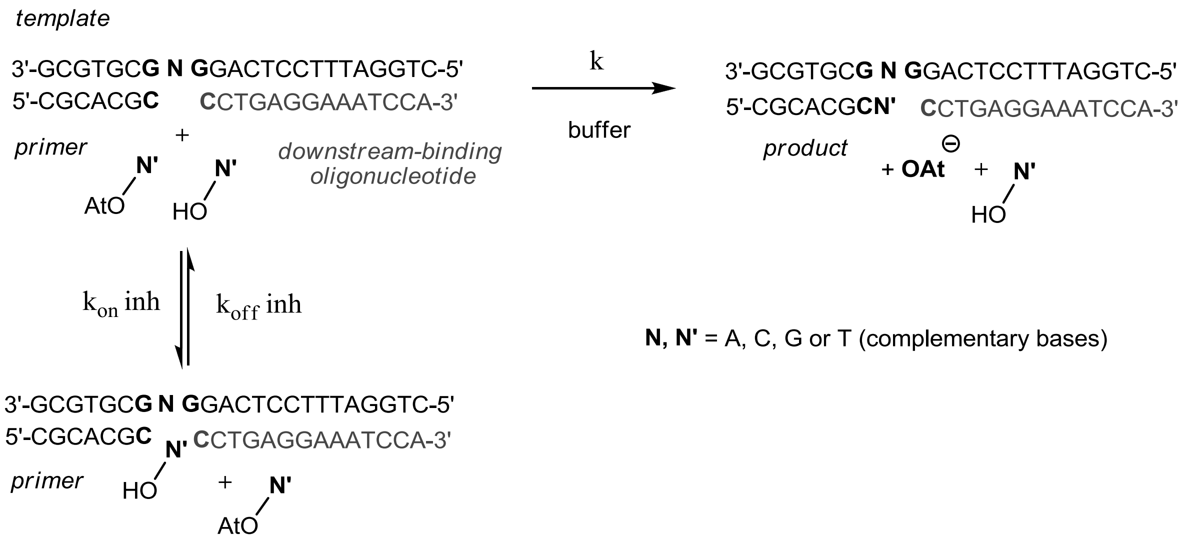

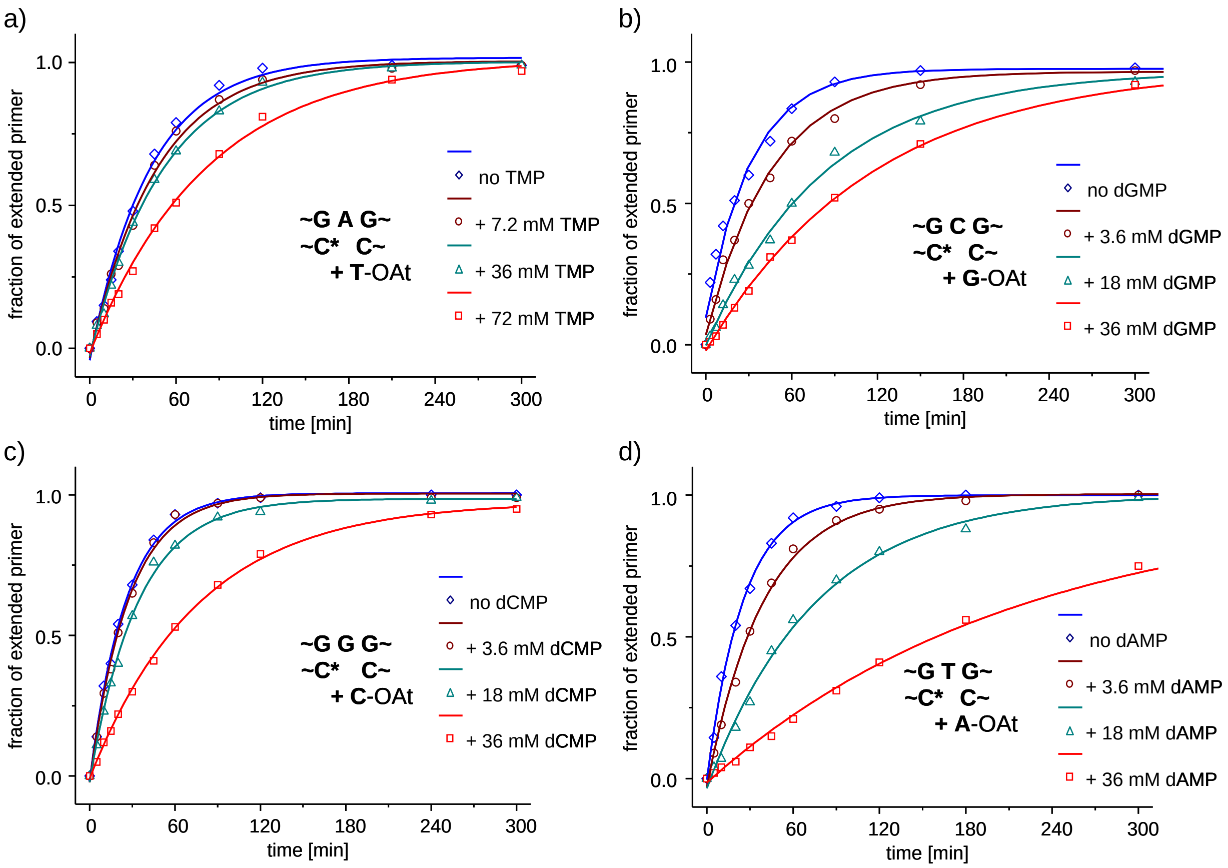

Figure S16. Kinetics of the extension reaction of primer 5'-CGCACG**C**-3'-NH_2 (**9c**, 36 µM) directed by GNG-type templates (**8gng**, 54 µM) with downstream-binding oligonucleotide (**10c**, 54 µM) at 20°C, using 3.6 mM dN'MP-OAt (for **7a**, **7c** or **7g**) or 7.2 mM TMP-OAt (**7t**) and various concentrations of corresponding inhibitor dNMP's (**1a**, **1c**, **1g**, or **1t**) in HEPES buffer (200 mM, pH 8.9, 400 mM NaCl, 80 mM $MgCl_2$). Symbols are experimental data, lines are fits from the exponential model.

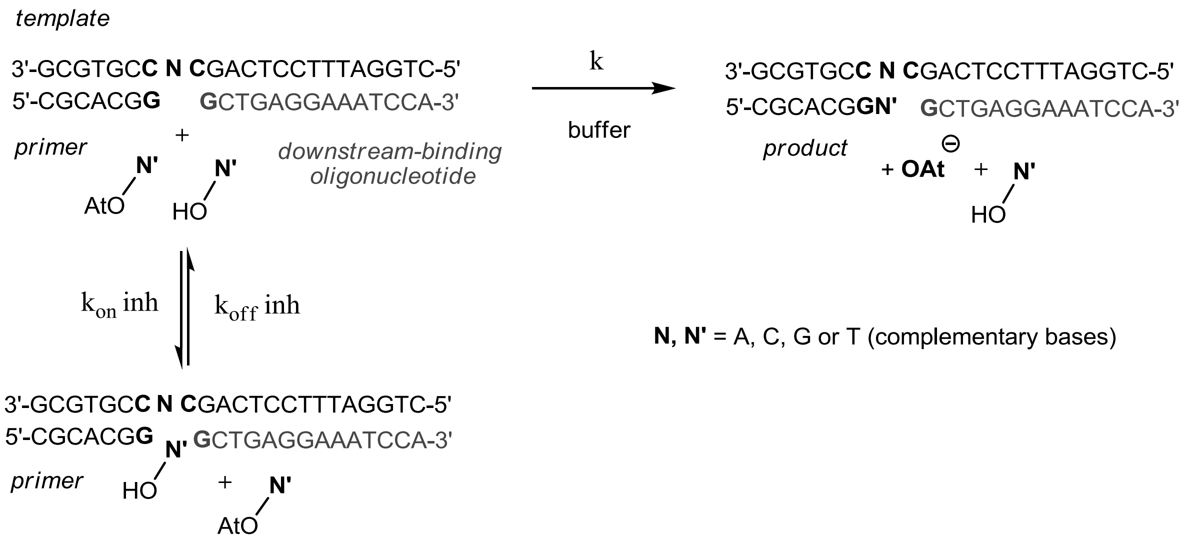

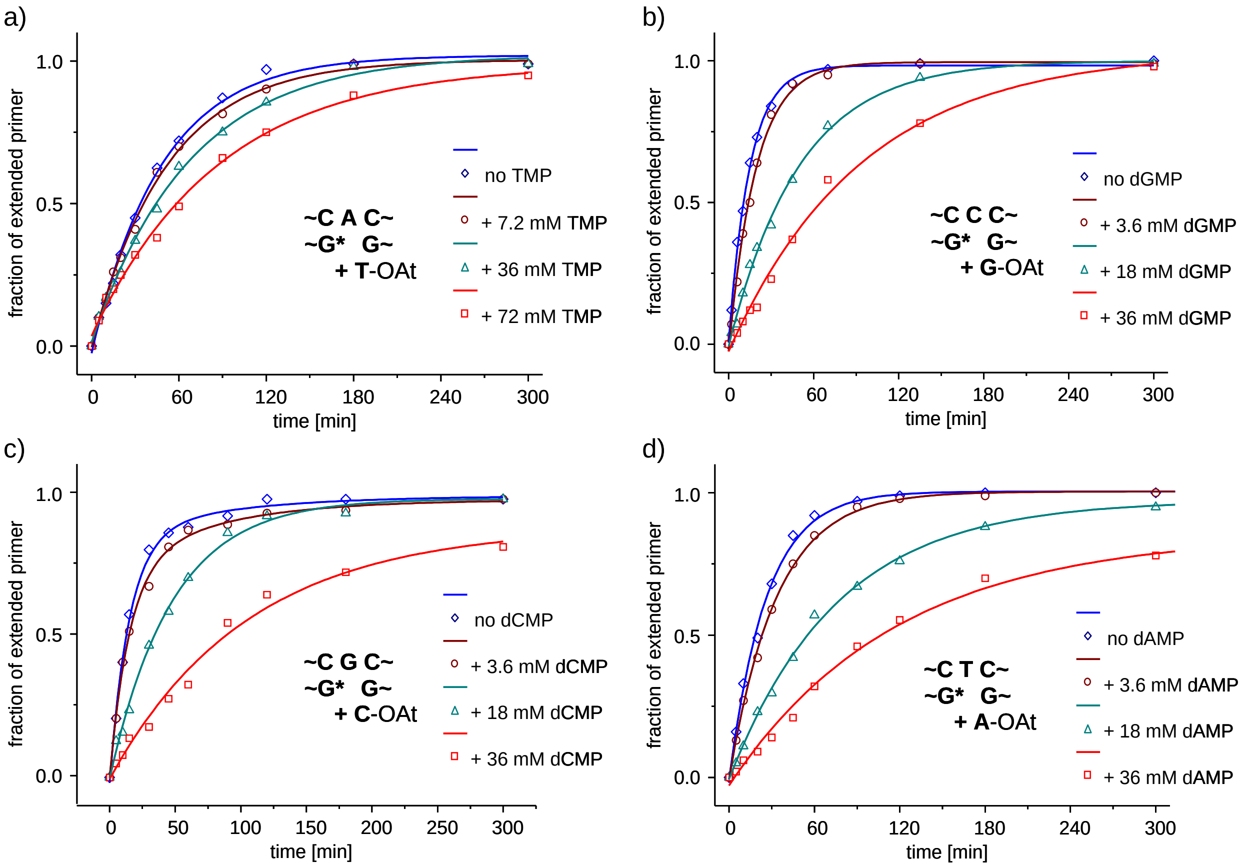

Figure S17. Kinetics of the extension reaction of primer 5'-CGCACG**G**-3'-NH_2 (**9g**, 36 µM) directed by CNC-type templates (**8cnc**, 54 µM) with downstream-binding oligonucleotide (**10g**, 54 µM) at 20°C, using 3.6 mM dN'MP-OAt (for **7a**, **7c** or **7g**) or 7.2 mM TMP-OAt (**7t**) and various concentrations of corresponding inhibitor dNMP's (**1a**, **1c**, **1g**, or **1t**) in HEPES buffer (200 mM, pH 8.9, 400 mM NaCl, 80 mM $MgCl_2$). Symbols are experimental data, lines are fits from the exponential model.

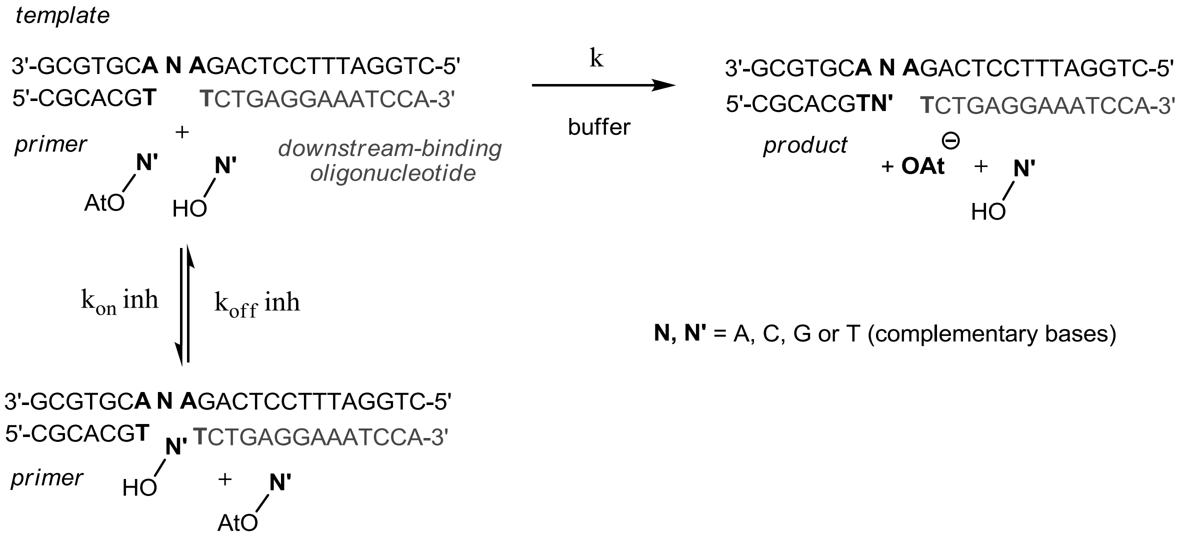

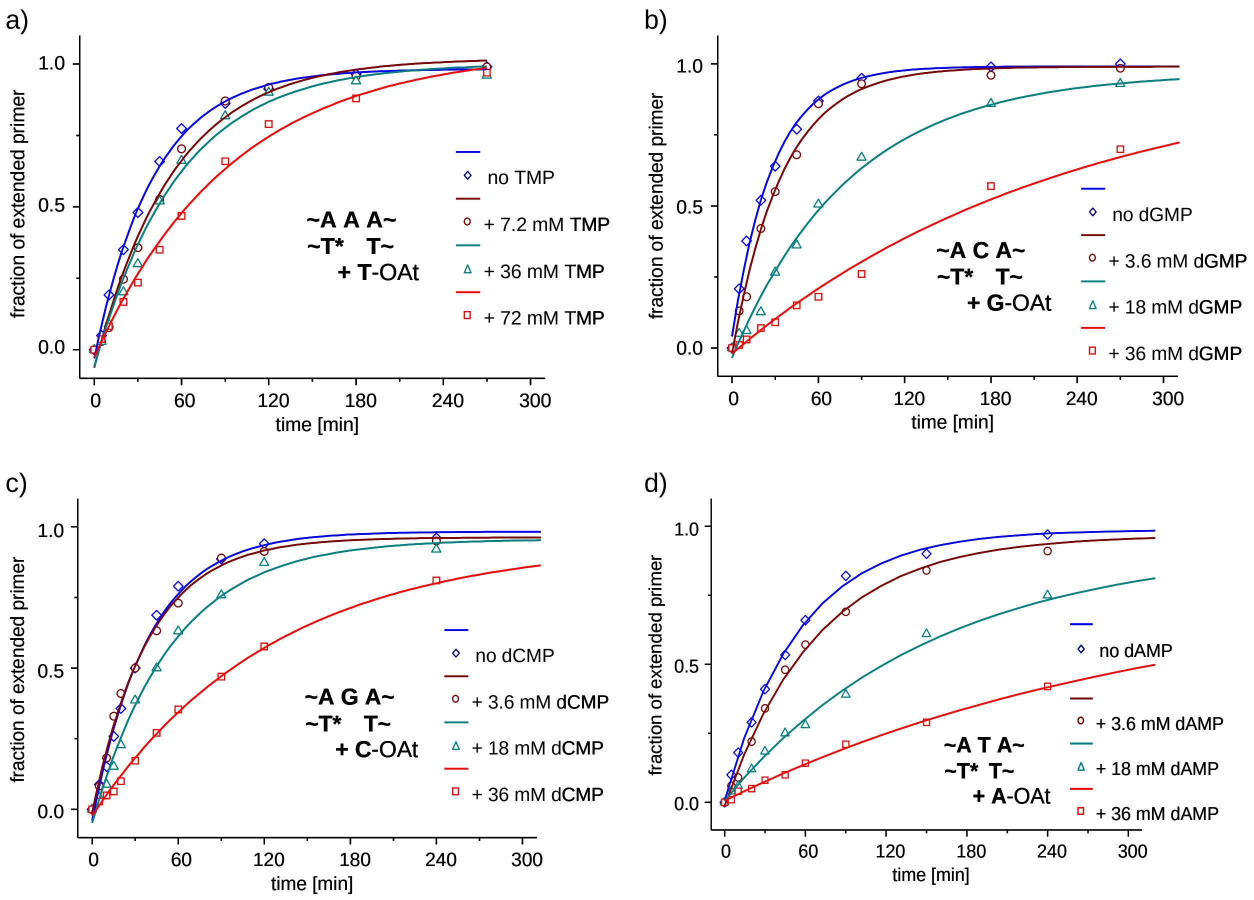

Figure S18. Kinetics of the extension of primer 5'-CGCACGT**T**-3'-NH$_2$ (**9t**, 36 µM) directed by ANA-type templates (**8ana**, 54 µM) with downstream-binding oligonucleotide (**10t**, 54 µM) at 20°C, using 3.6 mM dN'MP-OAt (for **7a**, **7c** or **7g**) or 7.2 mM TMP-OAt (**7t**) and various concentrations of corresponding inhibitor dNMP's (**1a**, **1c**, **1g**, or **1t**) in HEPES buffer (200 mM, pH 8.9, 400 mM NaCl, 80 mM MgCl$_2$). Symbols are experimental data, lines are fits from the exponential model.

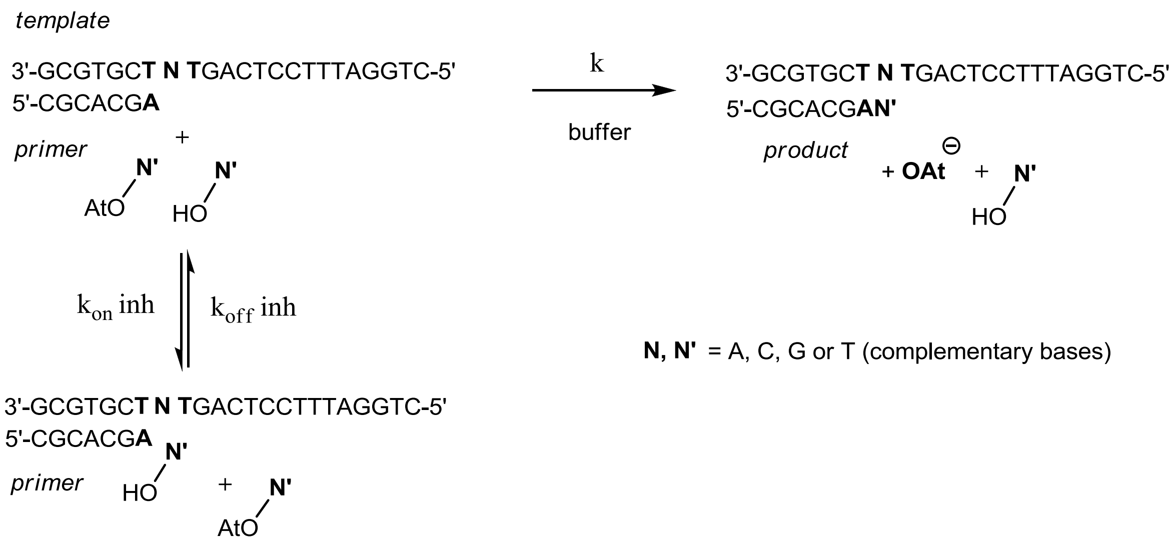

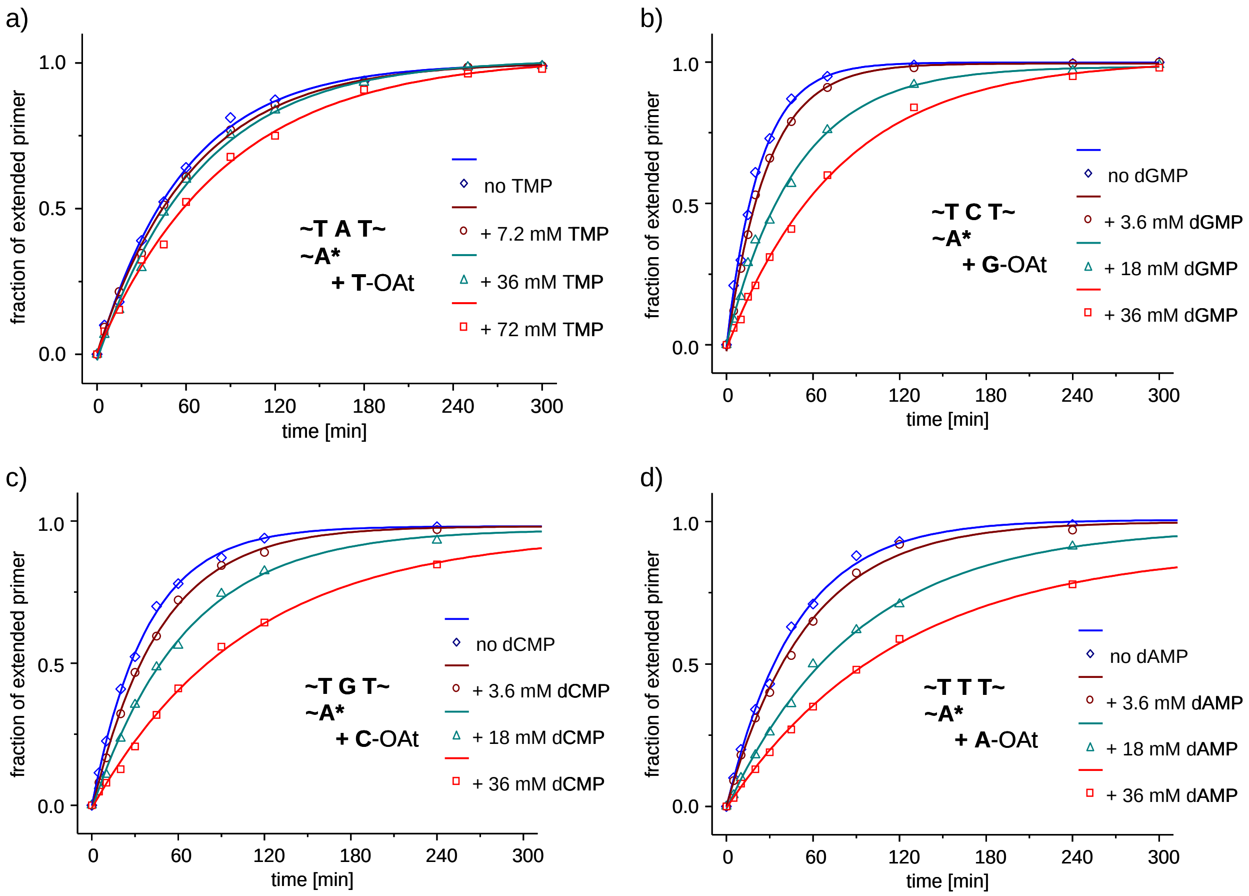

Figure S19. Kinetics of the extension reaction of primer 5'-CGCACG**A**-3'-NH_2 (**9a**, 36 µM) directed by TNT-type templates (**8tnt**, 54 µM) without downstream-binding oligonucleotide at 20°C, using 3.6 mM dN'MP-OAt (for **7a**, **7c** or **7g**) or 7.2 mM TMP-OAt (**7t**) and various concentrations of corresponding inhibitor dNMP's (**1a**, **1c**, **1g**, or **1t**) in HEPES buffer (200 mM, pH 8.9, 400 mM NaCl, 80 mM $MgCl_2$). Symbols are experimental data, lines are fits from the exponential model.

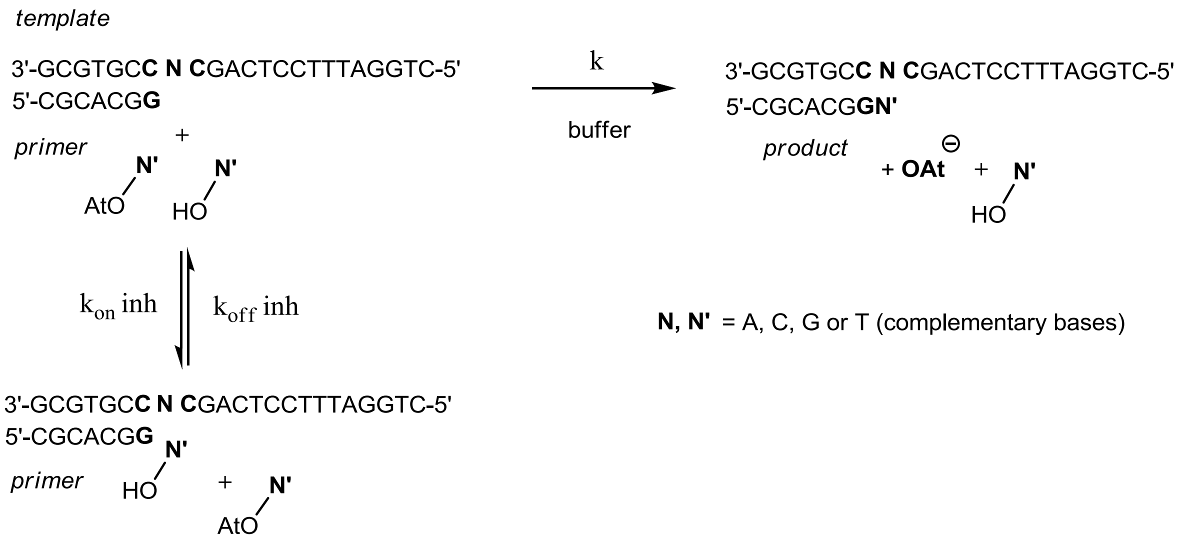

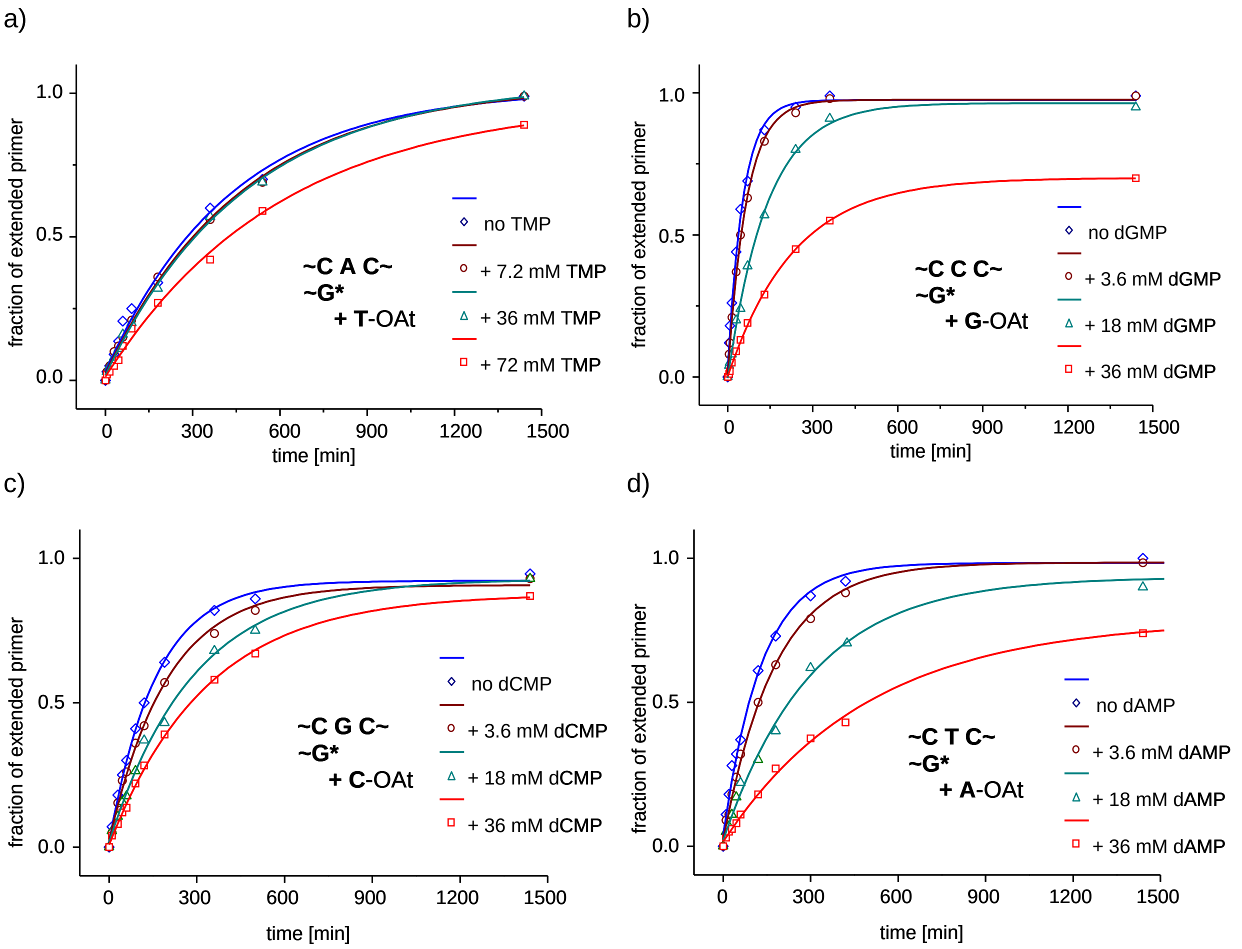

Figure S20. Kinetics of the extension reaction of primer 5'-CGCACGG**G**-3'-NH_2 (**9g**, 36 µM) directed by CNC-type templates (**8cnc**, 54 µM) without downstream-binding oligonucleotide at 10°C, using 3.6 mM dN'MP-OAt (for **7a**, **7c** or **7g**) or 7.2 mM TMP-OAt (**7t**) and various concentrations of corresponding inhibitor dNMP's (**1a**, **1c**, **1g**, or **1t**) in HEPES buffer (200 mM, pH 8.9, 400 mM NaCl, 80 mM $MgCl_2$). Symbols are experimental data, lines are fits from the exponential model.

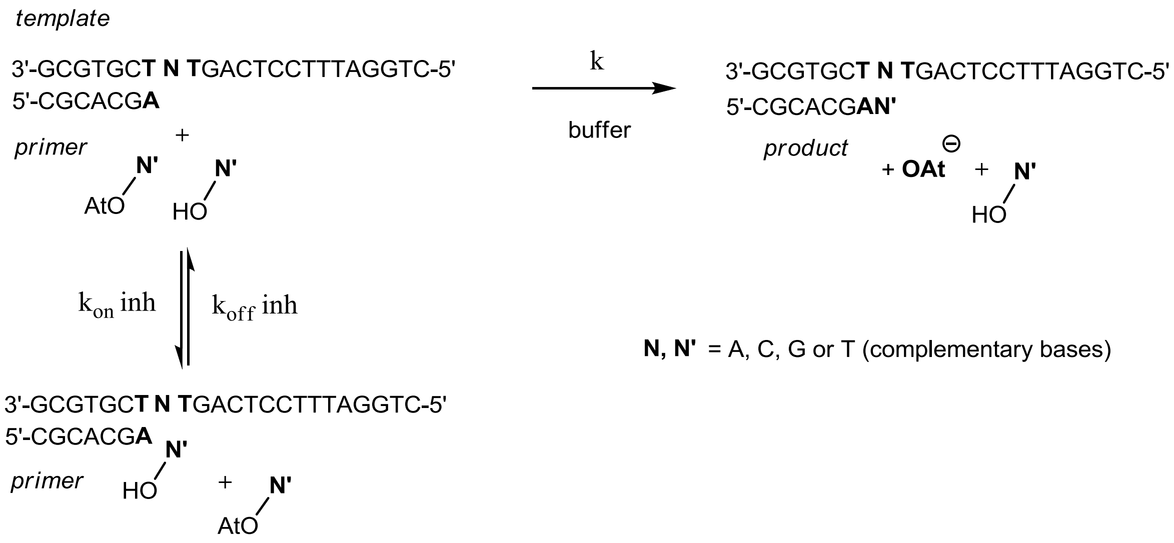

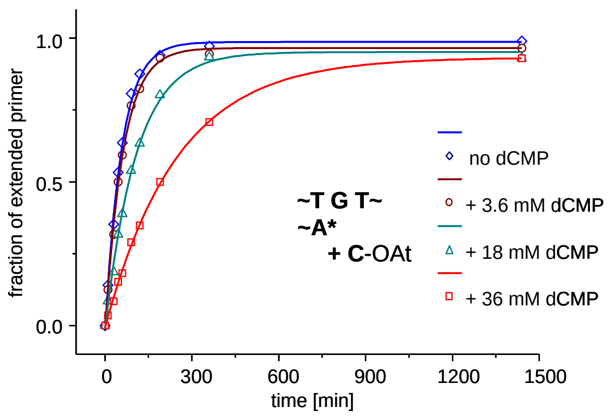

Figure S21. Kinetics of the extension of aminoterminal DNA primer 5'-CGCACG**A**-3'-NH_2 (**9a**, 36 µM), as directed by TGT-type template **8tgt** (54 µM) in the absence of an downstream-binding oligonucleotide at 10°C, using 3.6 mM dCMP-OAt (**7c**) and various concentrations of dCMP (**1c**) as inhibitor in HEPES buffer (200 mM, pH 8.9, 400 mM NaCl, 80 mM $MgCl_2$). Symbols are experimental data, lines are fits from the exponential model.

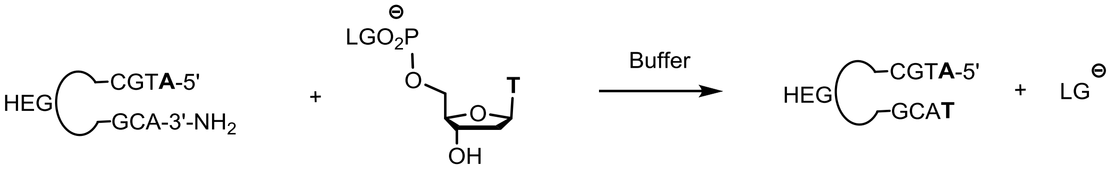

2a **7t** extended primer

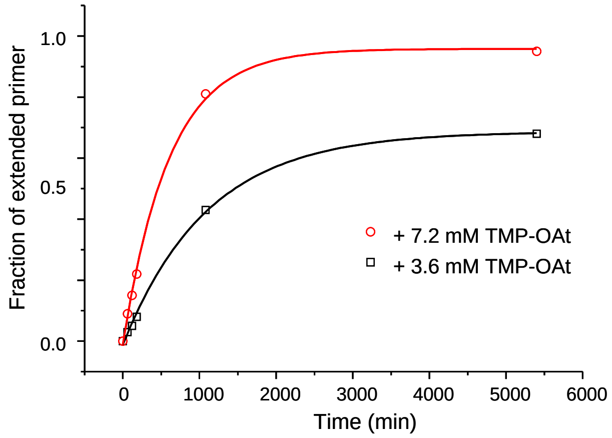

Figure S22. Kinetics of the extension reaction of hairpin 5'-ACG(HEG)CGT**A**-NH_2-3' (**2a**, 36 µM) at 20°C, using 3.6 mM or 7.2 mM TMP-OAt (**7t**) in HEPES buffer (200 mM, pH 8.9, 400 mM NaCl, 80 mM $MgCl_2$). The symbols are experimental data, the lines correspond to the exponential functions fitted.

Rate constants and Additional Data from Fits

Table S1. Inhibitory binding constants, rate constants, and r^2 values for fits to kinetic data.

Monomer[a] **7a-t/ 1a-t**	Template B^1NB^2	**8nnn**	helper[b]	Temp. (°C)	k (0eq.)[c] (min^{-1})	$(r^2)^d$	K_{Inh}[e] (mM^{-1})
**T**	TAT	**8tat**	+	20	0.0333	0.968	0.017
**G**	TCT	**8tct**	+	20	0.3621	0.979	0.625
**C**	TGT	**8tgt**	+	20	0.0856	0.964	0.218
**A**	TTT	**8ttt**	+	20	0.0960	0.962	0.185
**T**	GAG	**8gag**	+	20	0.0233	0.915	0.012
**G**	GCG	**8gcg**	+	20	0.0361	0.967	0.105
**C**	GGG	**8ggg**	+	20	0.0381	0.895	0.057
**A**	GTG	**8gtg**	+	20	0.0390	0.936	0.220
**T**	CAC	**8cac**	+	20	0.0366	0.971	0.009
**G**	CCC	**8ccc**	+	20	0.0656	0.996	0.146
**C**	CGC	**8cgc**	+	20	0.0533	0.947	0.159
**A**	CTC	**8ctc**	+	20	0.0079	0.954	0.145
**T**	AAA	**8aaa**	+	20	0.0225	0.938	0.013
**G**	ACA	**8aca**	+	20	0.0363	0.954	0.220
**C**	AGA	**8aga**	+	20	0.0234	0.922	0.066
**A**	ATA	**8ata**	+	20	0.0177	0.947	0.211
**T**	TAT	**8tat**	-	20	0.0167	0.978	0.005
**G**	TCT	**8tct**	-	20	0.0431	0.999	0.067
**C**	TGT	**8tgt**	-	20	0.0254	0.981	0.054
**A**	TTT	**8ttt**	-	20	0.0211	0.999	0.058
**T**	CAC	**8cac**	-	10	0.0025	0.865	0.007
**G**	CCC	**8ccc**	-	10	0.0187	0.930	0.206
**C**	CGC	**8cgc**	-	10	0.0055	0.986	0.034
**A**	CTC	**8ctc**	-	10	0.0079	0.954	0.145
**C**	TGT	**8tgt**	-	10	0.0167	0.946	0.103

[a] Monomer concentration: 3.6 mM for dAMP-OAt (**7a**), dCMP-OAt (**7c**), or dGMP-OAt (**7g**), and 7.2 mM for TMP-OAt (**7t)** (ensure full conversion of primer); 0, 1, 5 or 10 equivalent of base-matching unactivated monomer (**1a-t**) respectively.
[b] Presence or absence of downstream-binding oligonucleotide.
[d] Correlation (Chi-square coefficient).
[e] Inhibitory constant for matched dNMP (**1a-t**), as calculated from (slope x $k_{(0\ eq.)}/[M]_0$).

Plots for Determining Inhibitor Binding Constants

a)

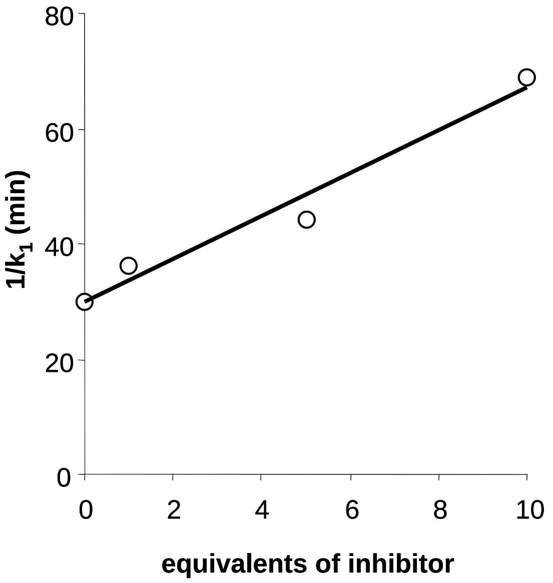

b)

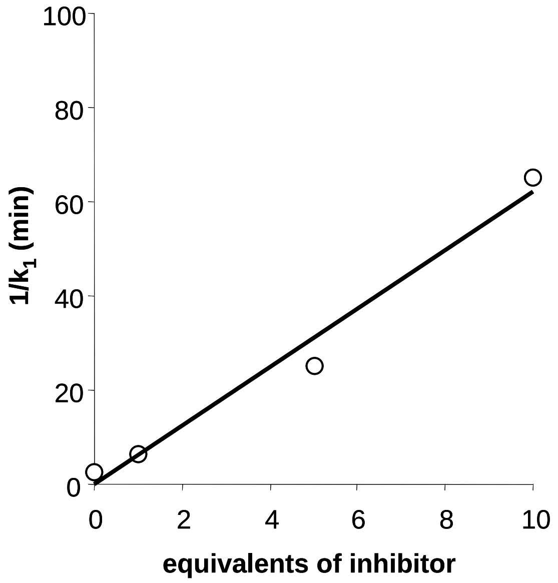

c)

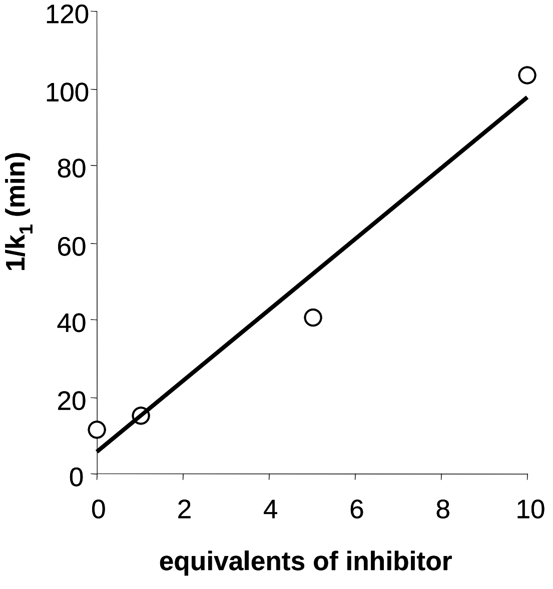

d)

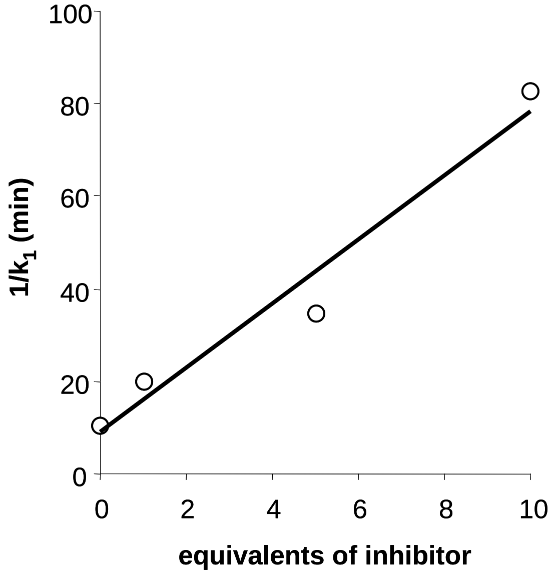

Figure S23. Plots of data used for the determination of K_{inh} values for the extension reaction of primer 5'-CGCACG**A**-3'-NH_2 (**9a**, 36 µM), as directed by T**N**T-type templates (**8tnt**, 54 µM) with downstream-binding oligonucleotide (**10a**, 54 µM) at 20°C, using 3.6 mM dN'MP-OAt (for **7a**, **7c** or **7g**) or 7.2 mM TMP-OAt (**7t**) and various concentrations of dNMP's as inhibitors (**1a**, **1c**, **1g** or **1t**) in HEPES buffer (200 mM, pH 8.9, 400 mM NaCl, 80 mM $MgCl_2$). The circles are experimental data, the lines are from the regression analysis. Fit results:

a) for dTMP **1t**, K_{Inh} = 0.017 mM^{-1}, r^2 = 0.968,
b) for dGMP **1g**, K_{Inh} = 0.625 mM^{-1}, r^2 = 0.979,
c) for dCMP **1c**, K_{Inh} = 0.218 mM^{-1}, r^2 = 0.964,
d) for dAMP **1a**, K_{Inh} = 0.185 mM^{-1}, r^2 = 0.962.

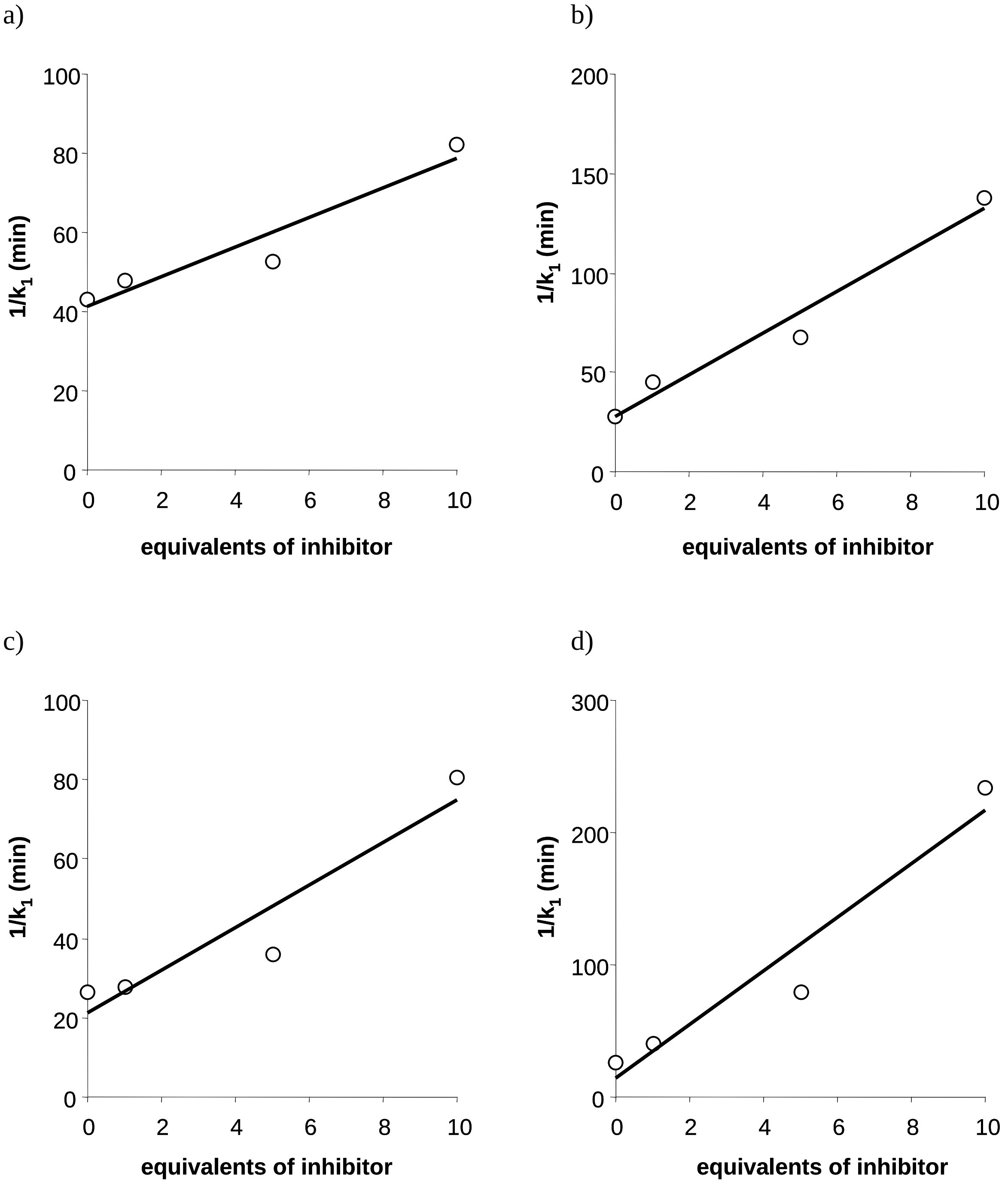

Figure S24. Plot for the determination of K_{inh} values for the extension reaction of primer 5'-CGCACG**C**-3'-NH_2 (**9c**, 36 µM) directed by G**N**G-type templates (**8gng**, 54 µM) with downstream-binding oligonucleotide (**10c**, 54 µM) at 20°C, using 3.6 mM dN'MP-OAt (for **7a**, **7c** or **7g**) or 7.2 mM TMP-OAt (**7t**) and various concentrations of dNMP's as inhibitors (**1a**, **1c**, **1g** or **1t**) in HEPES buffer (200 mM, pH 8.9, 400 mM NaCl, 80 mM $MgCl_2$). The circles are experimental data, the lines are from the regression analysis. Fit results:

a) for dTMP **1t**, $K_{Inh} = 0.012$ mM^{-1}, $r^2 = 0.915$,
b) for dGMP **1g**, $K_{Inh} = 0.105$ mM^{-1}, $r^2 = 0.967$,
c) for dCMP **1c**, $K_{Inh} = 0.057$ mM^{-1}, $r^2 = 0.895$,
d) for dAMP **1a**, $K_{Inh} = 0.220$ mM^{-1}, $r^2 = 0.936$.

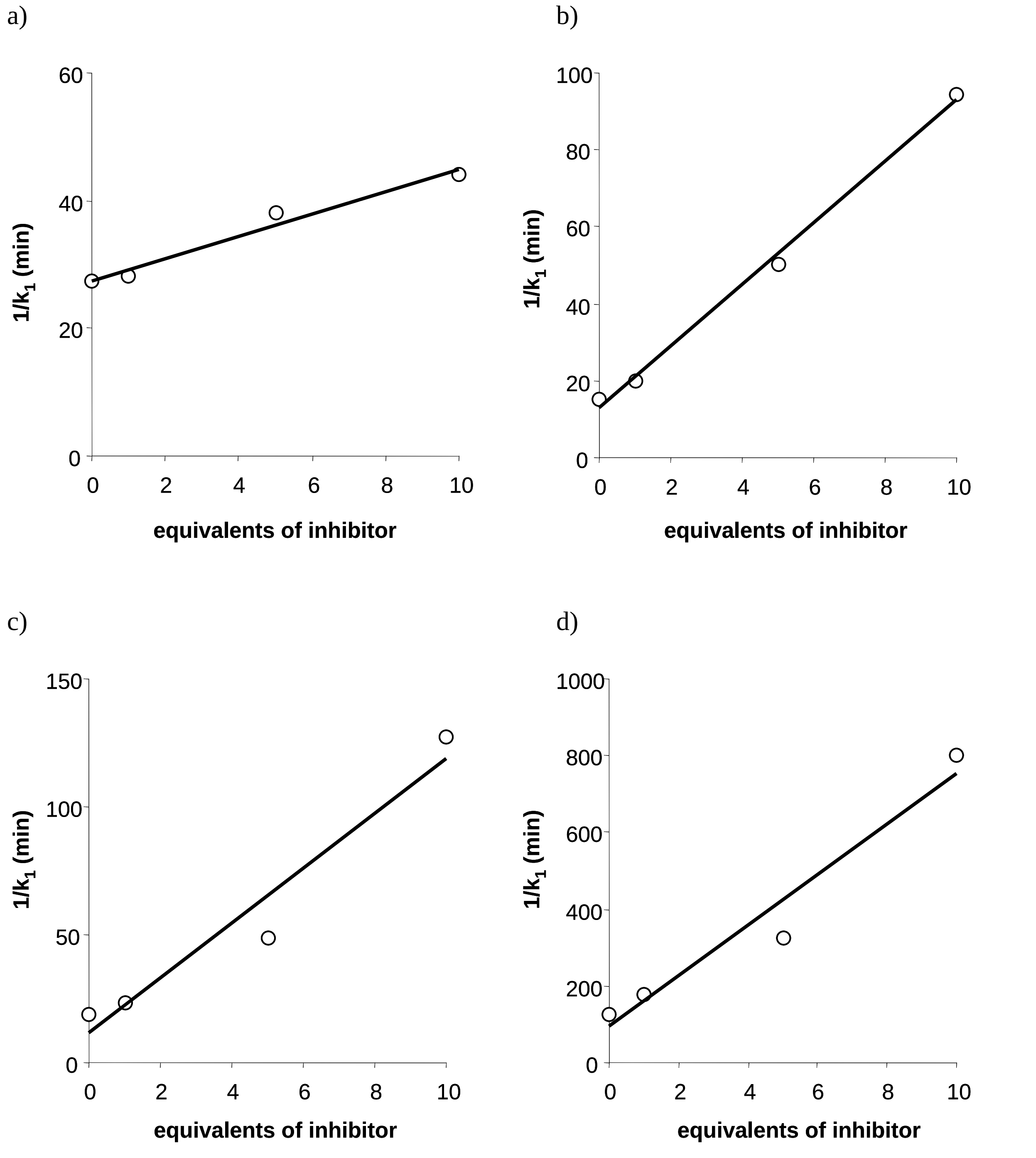

Figure S25. Plot for the determination of K_{inh} values for the extension reaction of primer 5'-CGCACGG**G**-3'-NH_2 (**9g**, 36 µM) directed by C**N**C-type templates (**8gng**, 54 µM) with downstream-binding oligonucleotide (**10g**, 54 µM) at 20°C, using 3.6 mM dN'MP-OAt (for **7a**, **7c** or **7g**) or 7.2 mM TMP-OAt (**7t**) and various concentrations of dNMP's as inhibitors (**1a**, **1c**, **1g** or **1t**) in HEPES buffer (200 mM, pH 8.9, 400 mM NaCl, 80 mM $MgCl_2$). The circles are experimental data, the lines are from the regression analysis. Fit results:

a) for dTMP **1t**, K_{Inh} = 0.009 mM^{-1}, r^2 = 0.971,
b) for dGMP **1g**, K_{Inh} = 0.146 mM^{-1}, r^2 = 0.996,
c) for dCMP **1c**, K_{Inh} = 0.159 mM^{-1}, r^2 = 0.947,
d) for dAMP **1a**, K_{Inh} = 0.145 mM^{-1}, r^2 = 0.954.

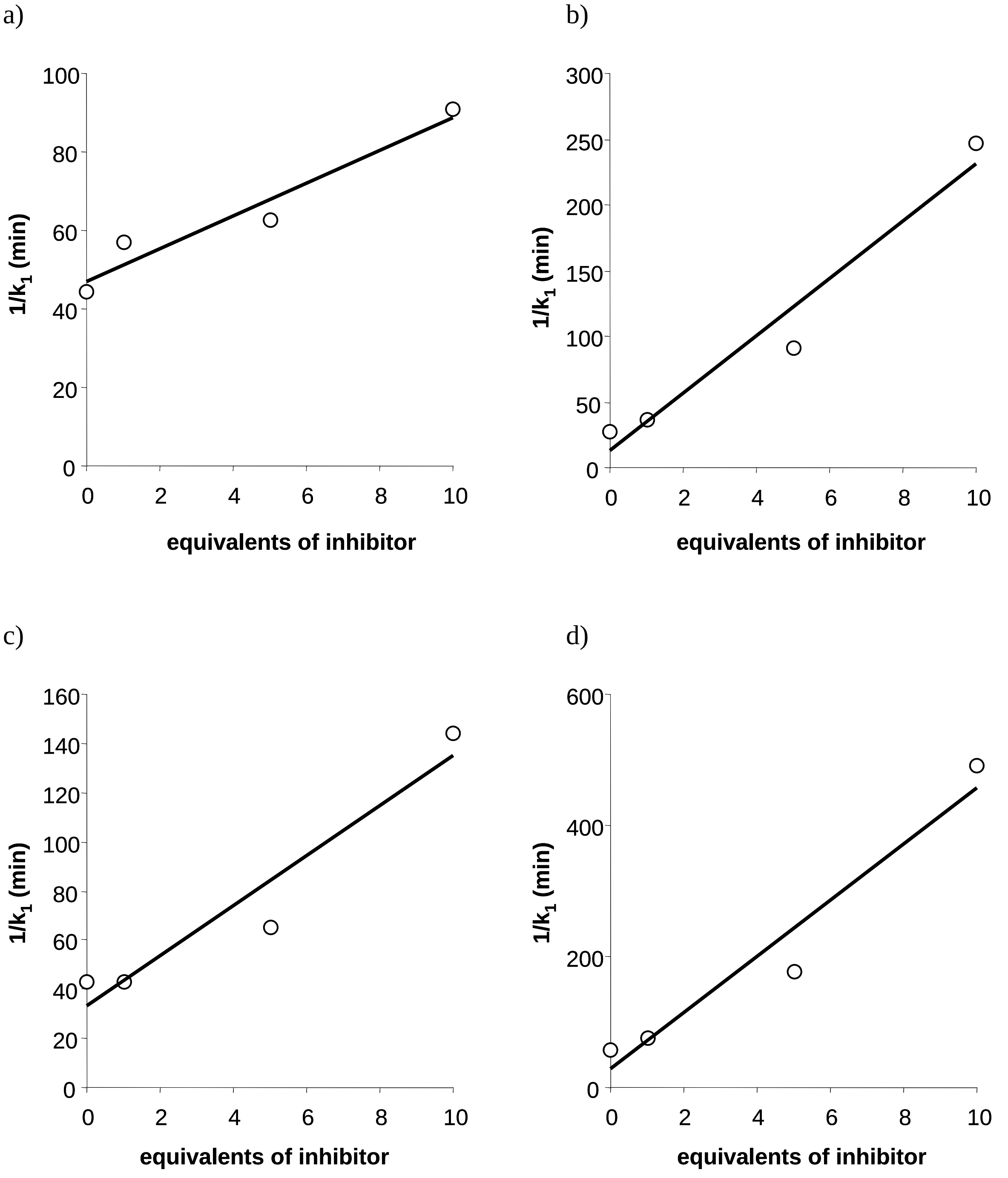

Figure S26. Plot for the determination of K_{inh} values for the extension reaction of primer 5'-CGCACG**T**-3'-NH_2 (**9t**, 36 µM) directed by A**N**A-type templates (**8ana**, 54 µM) with downstream-binding oligonucleotide (**10t**, 54 µM) at 20°C, using 3.6 mM dN'MP-OAt (for **7a**, **7c** or **7g**) or 7.2 mM TMP-OAt (**7t**) and various concentrations of dNMP's as inhibitors (**1a**, **1c**, **1g** or **1t**) in HEPES buffer (200 mM, pH 8.9, 400 mM NaCl, 80 mM $MgCl_2$). The circles are experimental data, the lines are from the regression analysis. Results:

a) for dTMP **1t**, K_{Inh} = 0.013 mM^{-1}, r^2 = 0.938,
b) for dGMP **1g**, K_{Inh} = 0.220 mM^{-1}, r^2 = 0.954,
c) for dCMP **1c**, K_{Inh} = 0.066 mM^{-1}, r^2 = 0.922,
d) for dAMP **1a**, K_{Inh} = 0.211 mM^{-1}, r^2 = 0.947.

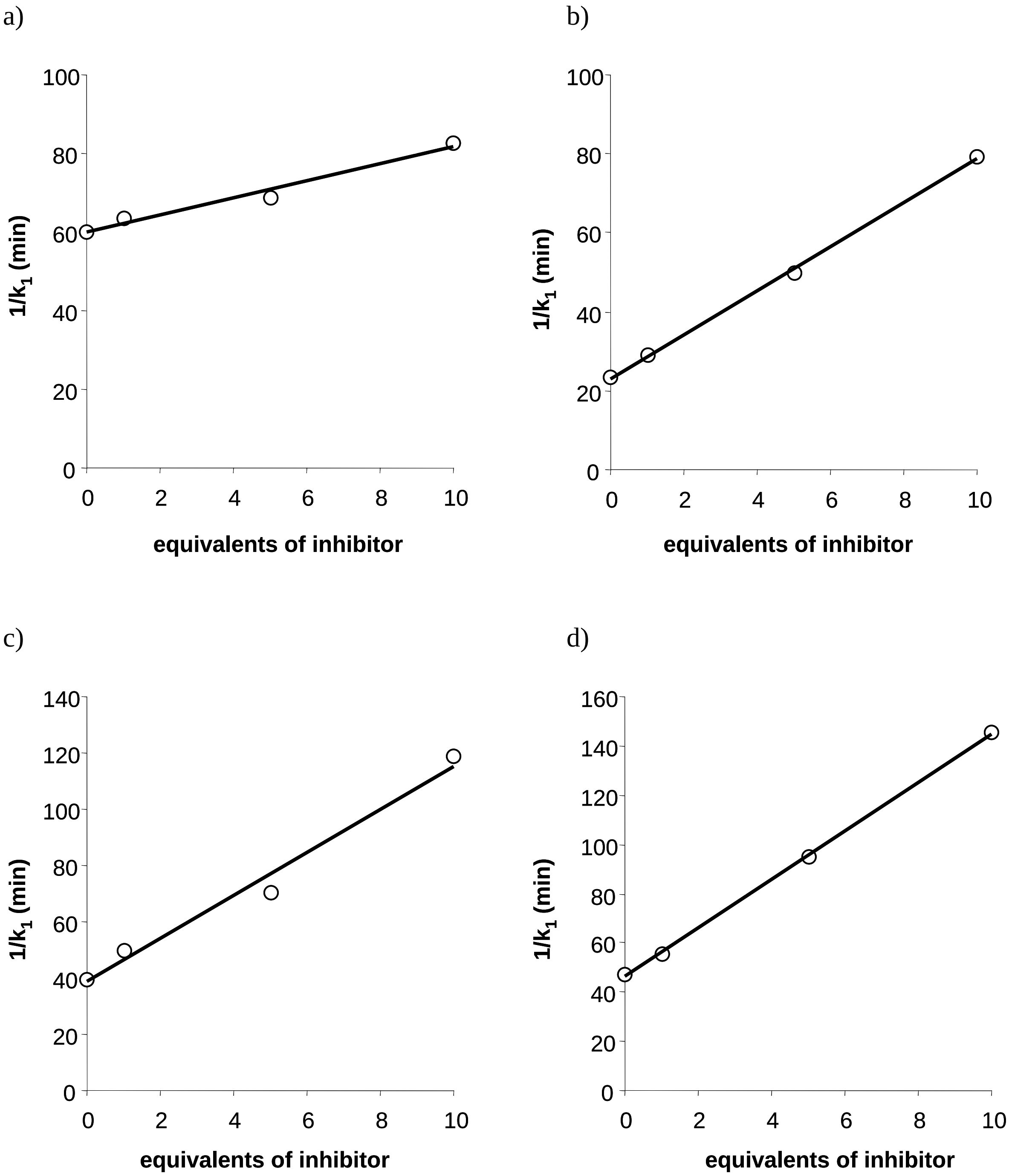

Figure S27. Plot for the determination of K_{inh} values for the extension reaction of primer 5'-CGCACG**A**-3'-NH_2 (**9a**, 36 µM) directed by TNT-type templates (**8tnt**, 54 µM) without downstream-binding oligonucleotide at 20°C, using 3.6 mM dN'MP-OAt (for **7a**, **7c** or **7g**) or 7.2 mM TMP-OAt (**7t**) and various concentrations of dNMP's as inhibitors (**1a**, **1c**, **1g** or **1t**) in HEPES buffer (200 mM, pH 8.9, 400 mM NaCl, 80 mM $MgCl_2$). The circles are experimental data, the lines are from the regression analysis. Fit results:

a) for dTMP **1t**, K_{Inh} = 0.005 mM^{-1}, r^2 = 0.978,
b) for dGMP **1g**, K_{Inh} = 0.067 mM^{-1}, r^2 = 0.999,
c) for dCMP **1c**, K_{Inh} = 0.054 mM^{-1}, r^2 = 0.981,
d) for dAMP **1a**, K_{Inh} = 0.058 mM^{-1}, r^2 = 0.999.

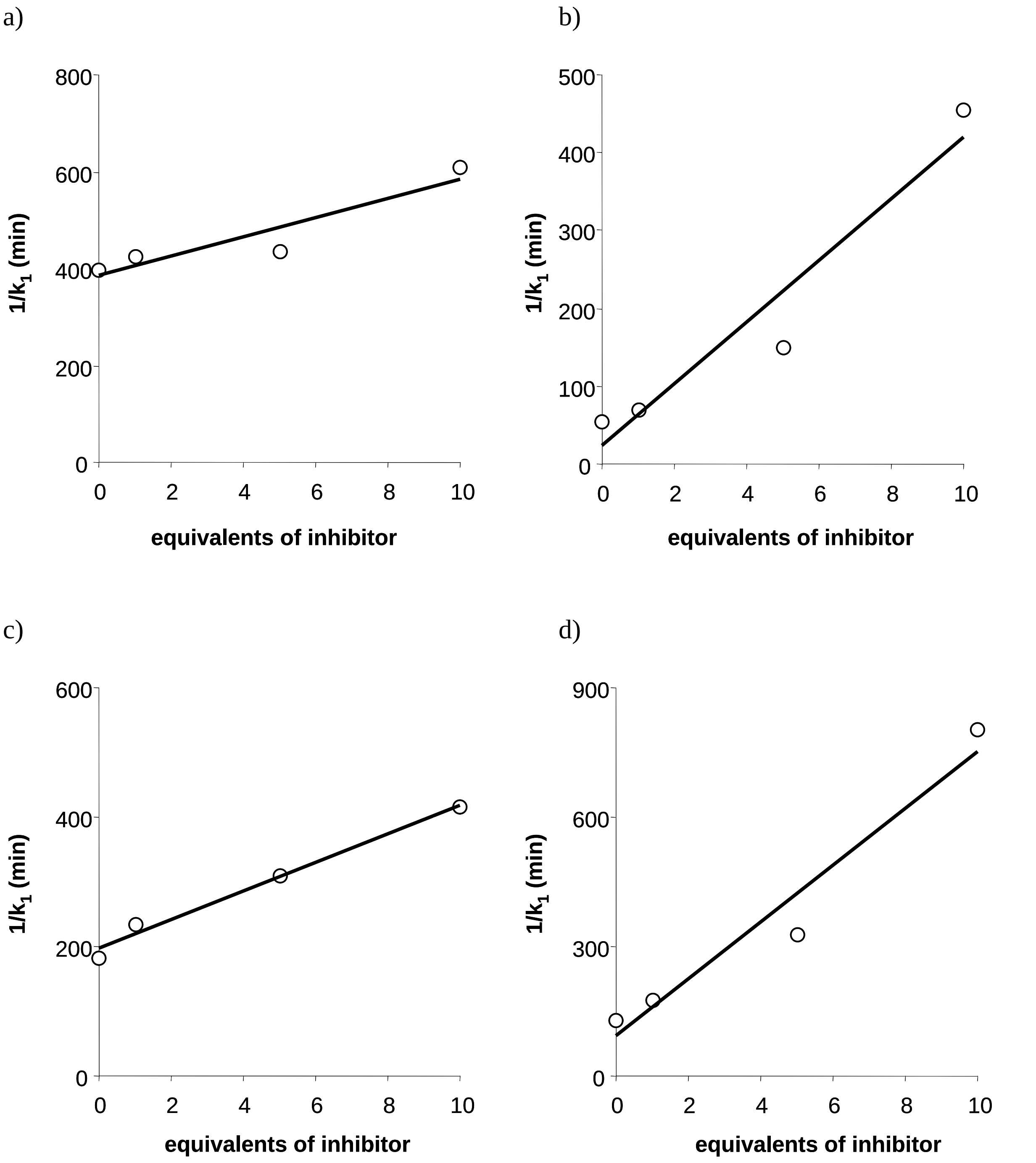

Figure S28. Plot for the determination of K_{inh} values for the extension reaction of primer 5'-CGCACG**G**-3'-NH_2 (**9a**, 36 µM) directed by C**N**C-type templates (**8cnc**, 54 µM) without downstream-binding oligonucleotide at 10°C, using 3.6 mM dN'MP-OAt (for **7a**, **7c** or **7g**) or 7.2 mM TMP-OAt (**7t**) and various concentrations of dNMP's as inhibitors (**1a**, **1c**, **1g** or **1t**) in HEPES buffer (200 mM, pH 8.9, 400 mM NaCl, 80 mM $MgCl_2$). The circles are experimental data, the lines are from the regression analysis. Fit results:

a) for dTMP **1t**, K_{Inh} = 0.007 mM^{-1}, r^2 = 0.865,
b) for dGMP **1g**, K_{Inh} = 0.206 mM^{-1}, r^2 = 0.930,
c) for dCMP **1c**, K_{Inh} = 0.034 mM^{-1}, r^2 = 0.986,
d) for dAMP **1a**, K_{Inh} = 0.145 mM^{-1}, r^2 = 0.954.

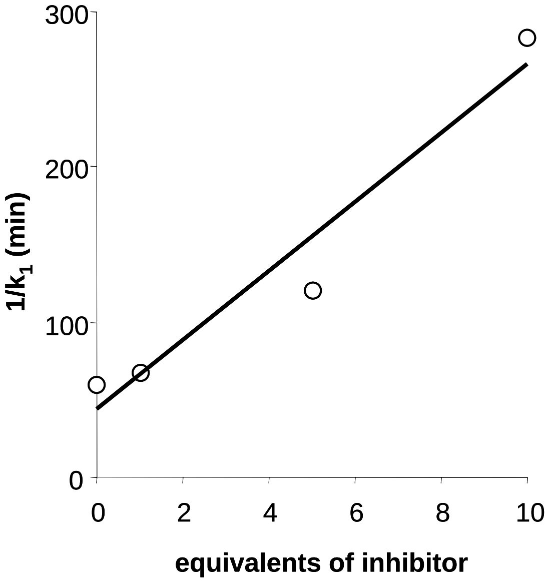

Figure S29. Plot for the determination of the K_{inh} value for the extension of primer 5'-CGCACG**A**-3'-NH_2 (**9a**, 36 µM), as directed by TGT template **8tgt** (54 µM) without downstream-binding oligonucleotide at 10°C, using 3.6 mM dGMP-OAt (**7g**) and various concentrations of dGMP (**1g**) as inhibitor in HEPES buffer (200 mM, pH 8.9, 400 mM NaCl, 80 mM $MgCl_2$). The circles are experimental data, the lines are from the regression analysis. Fit result (dGMP **1g**) K_{Inh} = 0.103 mM^{-1}, r^2 = 0.946.

Supplementary data.

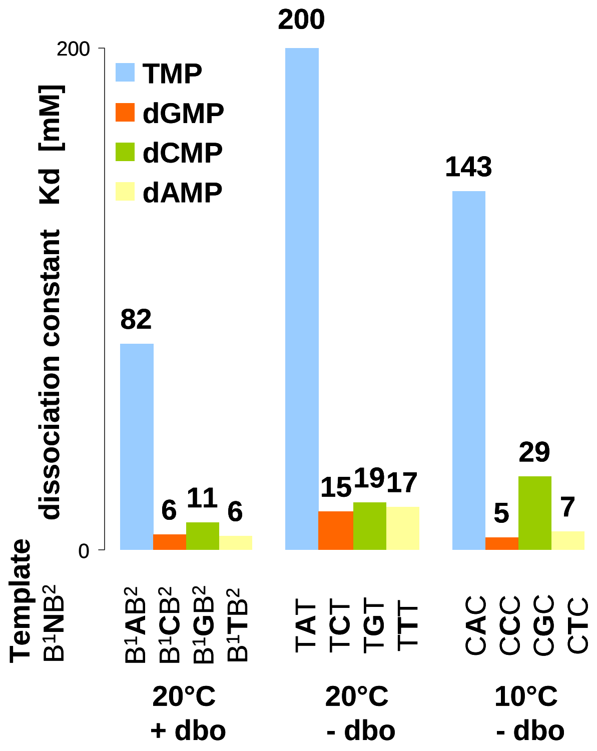

Figure S30. Association constants for dNMPs **1a-g** at primer extension sites for different templating sequence contexts and dNMP's. The effect of the presence of a downstream-binding oligonucleotide (dbo) and of the temperature are shown. See Table 2 for conditions. The K_d values for the B^1**N**B^2 template motifs on the left are average values (entries 1-16 of Table 2).

7. Model for More Elaborate Treatment of Inhibitor Kinetics

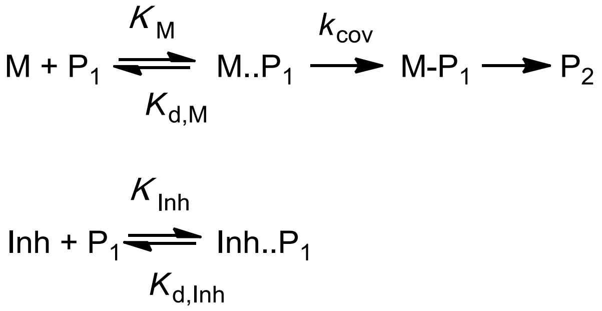

Scheme S1. Reaction scheme treating the pre-association of reacting monomer with primer-template complex explicitly.

In Scheme S1, reactive monomer M and inhibitor Inh are treated on the same footing in so far as both are explicitly considered as competitors in association equilibria with the extension site of the primer-template complex P_1. In case of the reactive monomer, the associate may lead to the formation of a covalent adduct M-P_1 with a rate constant k_{cov}. This reaction is considered to be irreversible, a scenario that was successfully applied in our earlier work.[S3] According to Scheme S1 the equilibrium concentration of the reactive intermediate M..P_1 is given by

$$[\mathrm{M..P_1}] = \frac{K_\mathrm{M}[\mathrm{M}]_0[\mathrm{P}]_\mathrm{tot}}{1 + K_\mathrm{M}[\mathrm{M}]_0 + K_\mathrm{Inh}[\mathrm{Inh}]} \qquad \text{(S5)}$$

and hence the effective first order rate constant for the disappearance of P_1

$$k_\mathrm{eff} = \frac{k_\mathrm{cov} K_\mathrm{M}[\mathrm{M}]_0}{1 + K_\mathrm{M}[\mathrm{M}]_0 + K_\mathrm{Inh}[\mathrm{Inh}]} \qquad \text{(S6)}$$

Equation (S6) was used for direct curve fitting of the observed k_{eff} vs. [Inh] data, while the assumption $K_\mathrm{M} = K_\mathrm{Inh}$ was used. The fits are shown in Figures S31 and S32, and the parameter values are compiled in Table S2. In the case of the TCT template reacting with the G monomer, the assumption of $K_\mathrm{M} = K_\mathrm{Inh}$ did not yield a satisfactory fit because the inhibition effect was stronger than implied in this condition. The parameters given for this case in Table S2 and in Figures S31 and S32 are subject to the assumption of $K_\mathrm{Inh} = 2.5\ K_\mathrm{M}$. Comparing the K_d values obtained by the simple and the more elaborate model shows that the differences are small.

Table S2. Parameters for curve fitting of kinetic model according to Scheme S1.

	Monomer	Template	Helper	T,°C	c_0, mM	k_{cov}, min^{-1}	K_d,mM	K_d, mM[(a)]
1	T	TAT	+	20	7.2	0.297	59	59
2	G	TCT	+	20	3.6	0.642[(b)]	3[(b)]	2
3	C	TGT	+	20	3.6	0.190	4	5
4	A	TTT	+	20	3.6	0.137	2	5
5	T	GAG	+	20	7.2	0.324	93	83
6	G	GCG	+	20	3.6	0.093	6	10
7	C	GGG	+	20	3.6	0.296	23	18
8	A	GTG	+	20	3.6	0.073	3	5
9	T	CAC	+	20	7.2	0.538	98	111
10	G	CCC	+	20	3.6	0.157	5	7
11	C	CGC	+	20	3.6	0.200	7	6
12	A	CTC	+	20	3.6	0.021	6	7
13	T	AAA	+	20	7.2	0.246	76	77
14	G	ACA	+	20	3.6	0.079	4	5
15	C	AGA	+	20	3.6	0.154	19	15
16	A	ATA	+	20	3.6	0.04	4	5
17	T	TAT	-	20	7.2	0.480	202	200
18	G	TCT	-	20	3.6	0.184	12	15
19	C	TGT	-	20	3.6	0.141	17	19
20	A	TTT	-	20	3.6	0.105	14	17
21	T	CAC	-	10	7.2	0.059	162	143
22	G	CCC	-	10	3.6	0.048	5	5
23	C	CGC	-	10	3.6	0.041	25	29
24	A	CTC	-	10	3.6	0.021	6	7
25	C	TGT	-	10	3.6	0.070	11	10

[(a)] For comparison, the K_d values from the simple model (Scheme S1) are shown.
[(b)] For the TCT template the fit required to choose $K_{Inh} > K_M$. The entry refers to a parameter value of $K_{d,Inh}$ = 1/0.90 mM = 1.1 mM.

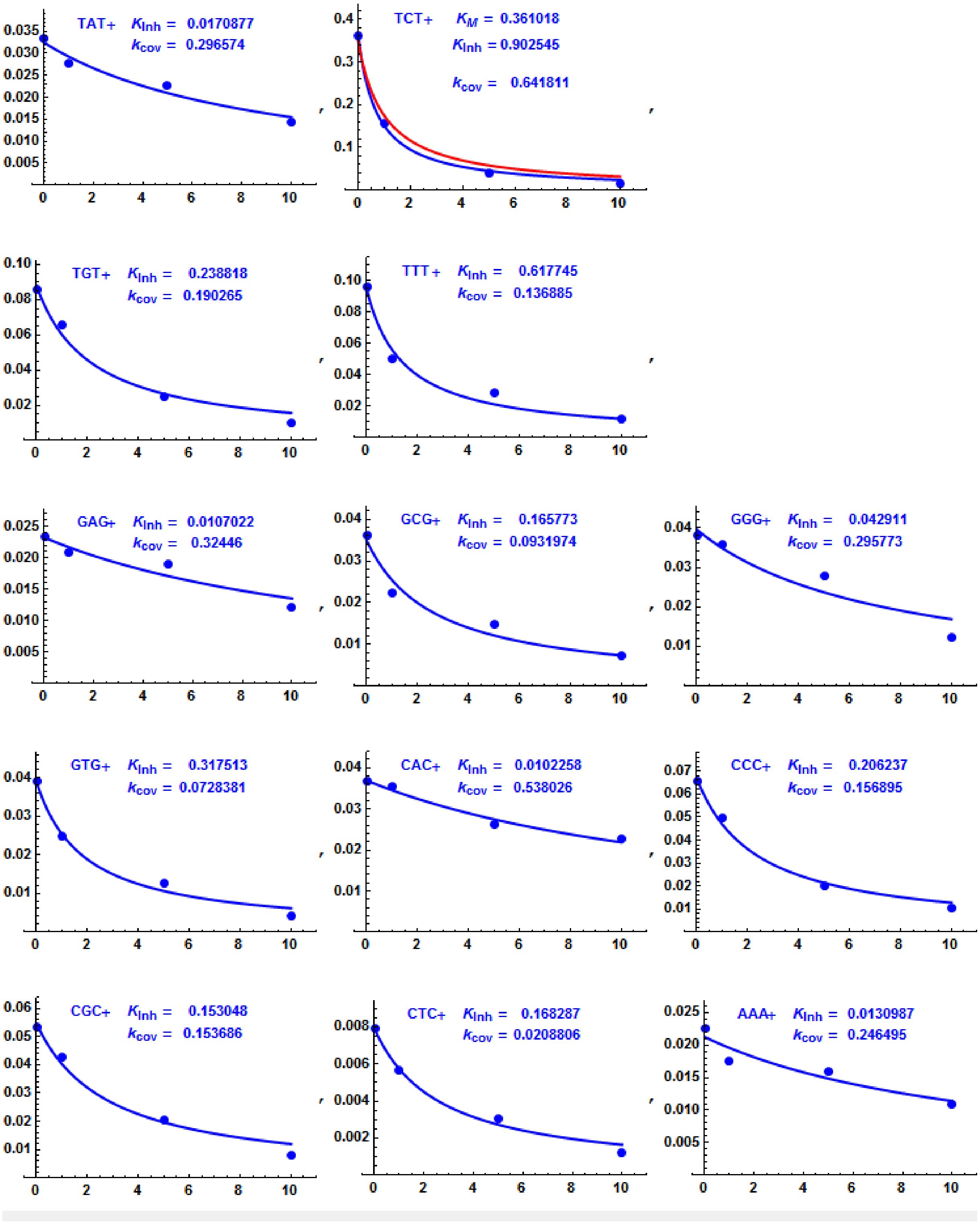

Figure S31. Plots of k, min^{-1}, vs. [Inh], in units of equivalents of $[M]_0$, for determining $K_{Inh} = K_M$ by direct curve fits according to Scheme S1, equation (S6). For specification of samples cf. captions to Figures S15-S21.

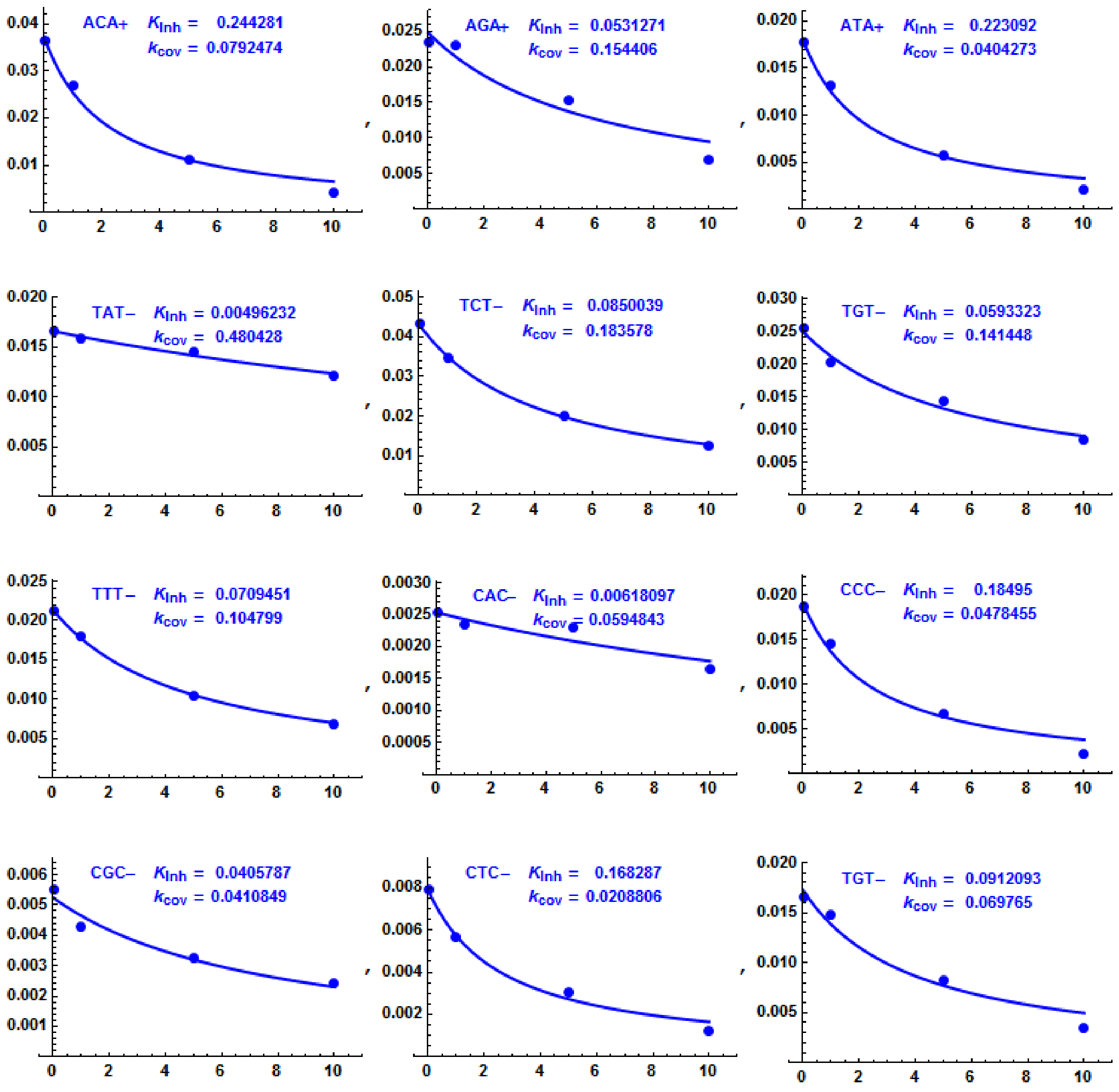

Figure S32. Plots of k, min^{-1}, vs. [Inh], in units of equivalents of $[M]_0$, for determining $K_{Inh} = K_M$ by direct curve fits according to Scheme S1, equation (S6). This figure is a continuation of Figure S31. In the case of the TCT template reacting with the G-monomer K_{Inh} and K_M had to be fitted separately.

8. Kinetics of Hydrolysis of OAt-Activated Monomers

The kinetics of the hydrolysis of OAt-activated monomers (**7a-t**) were measured by NMR from solutions containing the monomer (130 mM) in HEPES assay buffer (200 mM, containing 400 mM NaCl, and 80 mM $MgCl_2$) in D_2O:H_2O (1:9, *v*/*v*), pH 8.9, at 20°C. After stated intervalls, the reaction mixtures were analyzed by ^{31}P NMR at 121.5 MHz. Rate constants were calculated using a first order kinetic model and the fits were performed in Origin Pro 8.0 applying $f(t) = Y_0 + A_0.exp^{(-k.t)}$, where k is the rate constant, t is time, and Y_0 is the pre-exponential factor.

a) b)

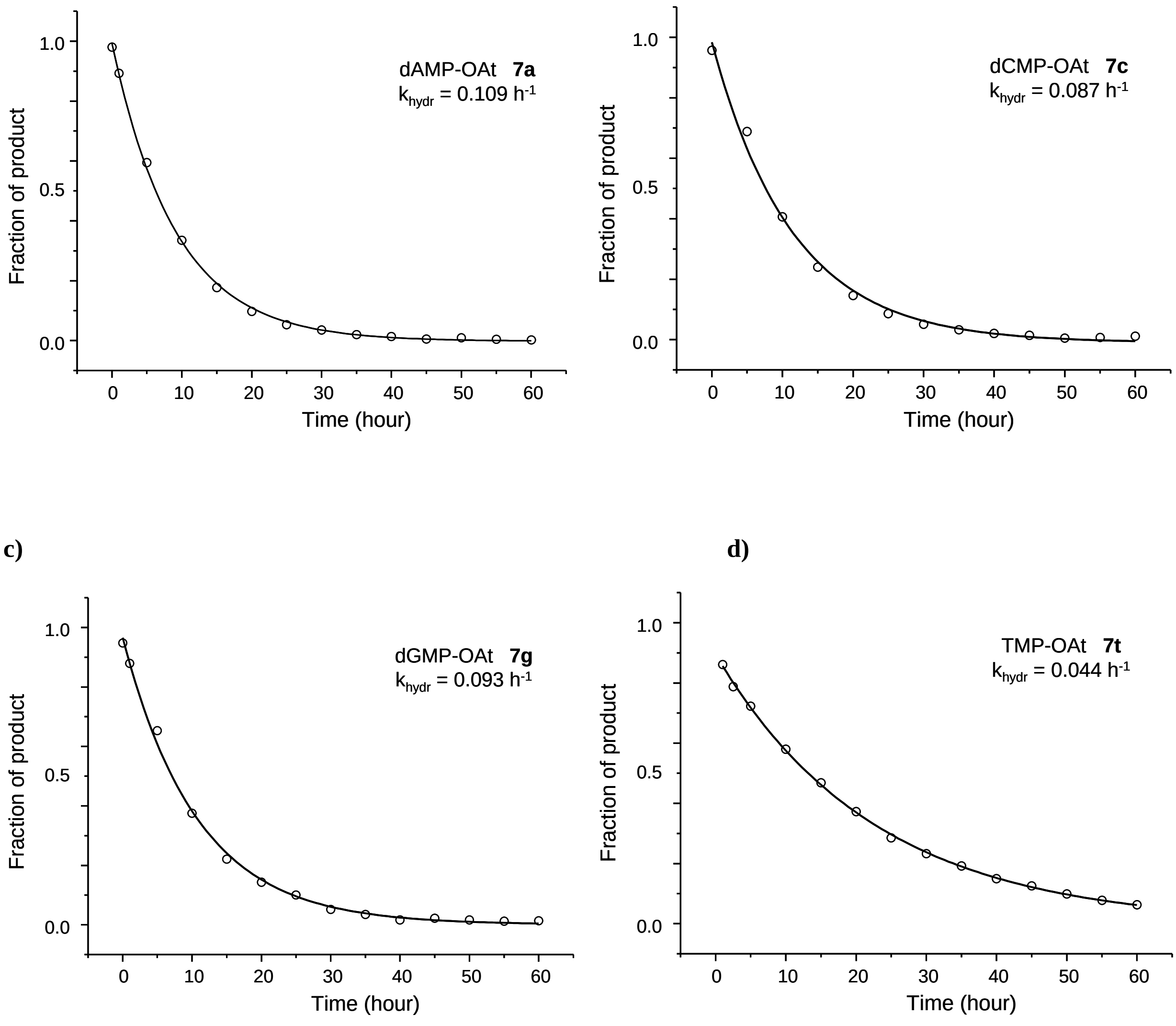

Figure S33. Kinetics of hydrolysis of OAt-esters of deoxynucleosides monophosphate. a) dAMP-OAt (**7a**), b) dCMP-OAt (**7c**), c) dGMP-OAt (**7g**), and d) TMP-OAt (**7t**).

9. Model for Primer Extension Including Monomer Hydrolysis and Inhibition

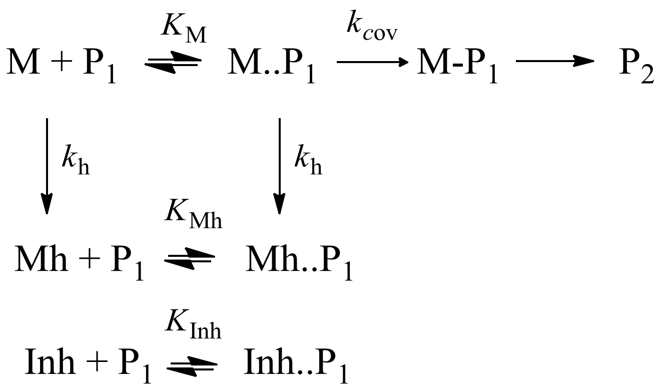

Scheme S2. Extended reaction scheme including inhibition and hydrolysis of reactive monomer

In Scheme 2, hydrolysis of the reactive monomer, described by first order kinetics with rate constant k_h, is included, as well as the inhibitory action of the hydrolyzed monomer Mh. The kinetic equations based on this scheme can be solved analytically, if it is assumed that the concentrations of monomer M and inhibitor Inh (if present as a separately added component) are in large excess over the concentration of primer-template complex P_1. The sum of the concentrations of reactive monomer M and hydrolyzed monomer Mh is constant:

$$[\mathrm{M}]+[\mathrm{Mh}]=[\mathrm{M}]_0 \qquad \text{(S7)}$$

First, it is assumed that all the equilibrium constants of associations with the primer-template complex are equal, viz.

$$K_{\mathrm{M}}=K_{\mathrm{Mh}}=K_{\mathrm{Inh}}\equiv 1/K_d \qquad \text{(S8)}$$

It will be further assumed that reaching the equilibria is much faster than the irreversible reactions of hydrolysis (rate constant k_h) and product formation (rate constant k_{cov}).

Denoting the sum $[P_1]$+ $[M..P_1]$ +$[Mh..P_1]$+ $[Inh..P_1]$ by $[P_1]_{tot}$, we can express the equilibrium concentration of the complex $M..P_1$ by

$$[\mathrm{M..P_1}]=\frac{K_M[\mathrm{M}][\mathrm{P_1}]_{\mathrm{tot}}}{1+K_M([\mathrm{M}]_0+[\mathrm{Inh}])}=\frac{[\mathrm{M}][\mathrm{P_1}]_{\mathrm{tot}}}{K_d+[\mathrm{M}]_0+[\mathrm{Inh}]} \qquad \text{(S9)}$$

The rate of primer extension is

$$\frac{d}{dt}[\mathrm{P_1}]_{\mathrm{tot}} = -k_{\mathrm{cov}}[\mathrm{M..P_1}] \tag{S10}$$

Combining this with equation (S_{10}) leads to

$$\frac{d}{dt}[\mathrm{P_1}]_{\mathrm{tot}} = -\frac{k_{\mathrm{cov}}[\mathrm{M}][\mathrm{P_1}]_{\mathrm{tot}}}{K_d + [\mathrm{M}]_0 + [\mathrm{Inh}]} \tag{S11}$$

The time dependence of the concentration of reactive monomer is given by the first order rate law

$$[\mathrm{M}] = [\mathrm{M}]_0 \exp\left(-k_h t\right) \tag{S12}$$

Substituting this function in equation (S11) and separating the variables t and $[\mathrm{P_1}]_{\mathrm{tot}}$, we can integrate both sides of the resulting differential equation separately. Solving the result for $[\mathrm{P_1}]_{\mathrm{tot}} / [\mathrm{P_1}]_{\mathrm{tot,0}}$ finally yields:

$$\frac{[\mathrm{P_1}]_{\mathrm{tot}}}{[\mathrm{P_1}]_{\mathrm{tot,0}}} = \exp\left[\frac{k_{\mathrm{cov}}[\mathrm{M}]_0(\exp[-k_{\mathrm{h}}\mathrm{t}] - 1)}{k_{\mathrm{h}}(K_d + [\mathrm{M}]_0 + [\mathrm{Inh}]\ )}\right] \tag{S13}$$

From this result, the yield of product can be obtained as

$$\mathrm{Y}_{\mathrm{P_2}}(t) = 1 - \frac{[\mathrm{P_1}]_{\mathrm{tot}}}{[\mathrm{P_1}]_{\mathrm{tot,0}}} = 1 - \exp\left[\frac{k_{\mathrm{cov}}[\mathrm{M}]_0(\exp[-k_{\mathrm{h}}\mathrm{t}] - 1)}{k_{\mathrm{h}}(K_d + [\mathrm{M}]_0 + [\mathrm{Inh}])}\right] \tag{S14}$$

At the limit of infinite time, this expression converges to

$$\mathrm{Y}_{\mathrm{P_2}}(\infty) = 1 - \exp\left[\frac{-k_{\mathrm{cov}}[\mathrm{M}]_0}{k_{\mathrm{h}}(\mathrm{K}_d + [\mathrm{M}]_0 + [\mathrm{Inh}])}\right] \tag{S15}$$

This expression can be rationalized by noting that the fraction

$$\frac{k_{\mathrm{cov}}[\mathrm{M}]_0}{\mathrm{K}_d + [\mathrm{M}]_0 + [\mathrm{Inh}]} = k \tag{S16}$$

represents the pseudo first order rate constant of primer reaction before any hydrolysis of the reactive monomer takes place. Thus the final yield can be cast into the simple form

$$\mathrm{Y}_{\mathrm{P_2}}(\infty) = 1 - \exp\left[\frac{-k}{k_{\mathrm{h}}}\right] = 1 - e^{-x} \tag{S17}$$

where x represents the ratio of first order rate constants of primer extension and monomer hydrolysis at the beginning when no hydrolysis has occurred yet. It is instructive to demonstrate by a few values how the final yield depends on this parameter x:

x	1	2	3	5	10
$Y_{P_2}(\infty)\,\%$	63	86	95	99,3	99,99..

Finally, we present the general solution to the kinetic scheme for the case where the three equilibrium constants are not equal. The result is

$$Y_{P_2}(t) = 1 - \frac{[P_1]_{tot}}{[P_1]_{tot,0}} = 1 - \exp\left[\frac{-k_{cov}K_M[M]_0}{k_h B}\left(k_h t - \ln\left(\frac{B + Ae^{k_h t}}{B + A}\right)\right)\right] \qquad (S18)$$

with the definitions:

$$A = 1 + K_{Mh}[M]_0 + K_{Inh}[Inh] \qquad (S19)$$

$$B = \left(K_M - K_{Mh}\right)[M]_0 + \varepsilon \to 0 \qquad (S20)$$

These relations have been used to calculate the curves in Figure 13b of the main text, where the inhibitor or hydrolyzed monomer are assumed to bind with a different binding constant than the reactive monomer.

Equation (S_{19}) comprises the special case of equal equilibrium constants for reactive and hydrolyzed monomer. For B = 0, the right hand side of equation (S_{19}) is not defined, but the limiting value for $B \to 0$ exists. For this reason, a small quantity ε has been added in equation (S_{21}). Reaching this limit analytically yields

$$Y_{P_2}(t) = 1 - \exp\left(\frac{k_{cov}K_M[M]_0\left\{\exp(-k_h t) - 1\right\}}{k_h A}\right) \qquad (S21)$$

10. References for Supporting Information

S1. Eisenhuth, R.; Richert, C. (2009) Convenient syntheses of 3'-amino-2',3'-dideoxynucleosides, their 5'-monophosphates, and 3'-aminoterminal oligodeoxynucleotide primers. *J. Org. Chem.,* **74**, 26-37.

S2. Hagenbuch, P.; Kervio, E.; Hochgesand, A.; Plutowski, U.; Richert, C. (2005) Chemical primer extension: efficiently determining single nucleotides in DNA. *Angew. Chem. Int. Ed.,* **40**, 6588–6592.

S3. Kervio, E.; Hochgesand, A.; Steiner, U.; Richert, C. (2010) Templating efficiency of naked DNA. *Proc. Natl. Acad. Sci. U. S. A.,* **107**, 12074–12079.
